# Transcriptome Atlases of Mouse Brain Reveals Differential Expression Across Brain Regions and Genetic Backgrounds

**DOI:** 10.1534/g3.111.001602

**Published:** 2012-02-01

**Authors:** Wei Sun, Seunggeun Lee, Vasyl Zhabotynsky, Fei Zou, Fred A. Wright, James J. Crowley, Zaining Yun, Ryan J. Buus, Darla R. Miller, Jeremy Wang, Leonard McMillan, Fernando Pardo-Manuel de Villena, Patrick F. Sullivan

**Affiliations:** * Department of Genetics; † Department of Biostatistics; ‡ Department of Computer Sciences; § Lineberger Comprehensive Cancer Center, University of North Carolina at Chapel Hill, North Carolina 27599

**Keywords:** Mouse Genetic Resource, Mouse Collaborative Cross, mouse, gene expression, whole brain, forebrain, hindbrain, sequence variation

## Abstract

Mouse models play a crucial role in the study of human behavioral traits and diseases. Variation of gene expression in brain may play a critical role in behavioral phenotypes, and thus it is of great importance to understand regulation of transcription in mouse brain. In this study, we analyzed the role of two important factors influencing steady-state transcriptional variation in mouse brain. First we considered the effect of assessing whole brain *vs.* discrete regions of the brain. Second, we investigated the genetic basis of strain effects on gene expression. We examined the transcriptome of three brain regions using Affymetrix expression arrays: whole brain, forebrain, and hindbrain in adult mice from two common inbred strains (C57BL/6J *vs.* NOD/ShiLtJ) with eight replicates for each brain region and strain combination. We observed significant differences between the transcriptomes of forebrain and hindbrain. In contrast, the transcriptomes of whole brain and forebrain were very similar. Using 4.3 million single-nucleotide polymorphisms identified through whole-genome sequencing of C57BL/6J and NOD/ShiLtJ strains, we investigated the relationship between strain effect in gene expression and DNA sequence similarity. We found that *cis*-regulatory effects play an important role in gene expression differences between strains and that the *cis*-regulatory elements are more often located in 5′ and/or 3′ transcript boundaries, with no apparent preference on either 5′ or 3′ ends.

The mouse is widely used as a model organism for human behavior studies because of the ability to manipulate its genome, to access brain tissues, and to effectively measure mouse behavior phenotypes (Bucan and Abel 2002). Among the many approaches to the dissection of the genetic/molecular basis of behavior phenotypes, the study of transcriptome in mouse brain has been commonly used and effective (Fernandes *et al.* 2004; Nadler *et al.* 2006). Here, we aimed to assess the role of two important factors on variation of gene expression: the effects of strain and brain region. In a few studies authors have assessed the effects of strain and/or brain region using microarray gene expression data. In an early study on six brain regions of mouse strain 129SvEv and C57BL/6 investigators found that only 1% to 2% of genes are differentially expressed across brain regions or strains (Pavlidis and Noble 2001), but this result can be partly attributed to small sample size and conservative thresholding. A study of hippocampal gene expression in eight mouse strains reported >200 genes showing strain differences (Fernandes *et al.* 2004). Su and coworkers reported a gene expression atlas across multiple human and mouse tissues including several mouse brain regions (Su *et al.* 2004). Regional differences in gene expression have been shown to reflect the embryologic imprint (Zapala *et al.* 2005). A larger study of gene expression differences across seven brain regions and 10 strains identified more than 9000 transcripts differentially expressed across brain regions and more than 6000 transcripts showing strain effects (Nadler *et al.* 2006). In addition, an anatomically comprehensive digital atlas by *in situ* hybridization has been reported (Lein *et al.* 2007).

In this article, we studied strain and brain region effects from different perspectives. First, assuming that previous knowledge is limited and resources are insufficient to assess gene expression in a large number of discrete brain regions, we tested the effect of assessing steady-state transcription in whole brain *vs.* in forebrain. Second, to determine the effect of genetic diversity on differential gene expression we evaluated two common inbred strains, C57BL/6J *vs.* NOD/ShiLtJ. We selected these strains because they are among the founders of new genetic resource populations known as the Collaborative Cross and the Diversity Outcross (Collaborative Cross Consortium 2012; Svenson *et al.* 2012). The level of sequence similarity varies widely across the genome ranging from complete identity by descent in some regions to regions with haplotypes derived from different house mouse subspecies (Yang *et al.* 2011). Furthermore, C57BL/6J has been sequenced and used to assemble the mouse reference genome (Waterston *et al.* 2002), and NOD/ShiLtJ is among the 17 inbred strains recently sequenced by the Wellcome Trust/Sanger Institute (Keane *et al.* 2011).

We found that although there are significant differences between transcriptomes of forebrain and hindbrain, whole brain and forebrain are very similar. Transcripts with stronger strain effects were significantly more likely to be located in genomic regions of high genetic diversity between strains. Among approximately 4100 transcripts with variable levels of sequence similarity between C57BL/6J and NOD/ShiLtJ, those with high level of sequence similarity at the 5′ and/or 3′ region of the transcripts were less likely to be differentially expressed. These findings strongly suggest that *cis*-regulatory effects play an important role in gene expression difference between strains, and the *cis*-regulatory elements are more often located in 5′ and/or 3′ transcript boundaries. Our data also suggest that there is no apparent preference for *cis*-regulatory elements to be located on either 5′ or 3′ ends.

## Materials and Methods

### Experimental design

The design of the study is summarized in Table 1. We studied brain samples contrasting the effects of strain (C57BL/6J *vs.* NOD/ShiLtJ adult male mice) and an acute drug treatment with a prototypical second-generation antipsychotic (clozapine *vs.* vehicle) with four mice per cell (16 mice in total). Three brain regions were studied: one hemisphere was used intact (whole brain) and the other hemisphere was separated into two portions, forebrain and hindbrain, as dictated by randomization. Total RNA samples were thus available from 48 samples (2 strains × 2 drug treatments × 3 brain regions × 4 mice). Because drug treatments showed little effect on gene expression variation, essentially we consider our design as (2 strains × 3 brain regions × 8 mice). Gene expression was measured by both Affymetrix standard cartridge format (1.0ST arrays, each sample hybridized separately) and the newer peg format (1.1ST arrays, samples assayed in two batches of 24 samples).

**Table 1:** Brain tissues analyzed by gene expression microarrays

Strain	Treatment	Mice	Whole Brain	Forebrain	Hindbrain
NOD/ShiLtJ	Vehicle	4	4 (2R + 2L)	4 (2R + 2L)	4 (2R + 2L)
NOD/ShiLtJ	Clozapine	4	4 (2R + 2L)	4 (2R + 2L)	4 (2R + 2L)
C57BL/6J	Vehicle	4	4 (2R + 2L)	4 (2R + 2L)	4 (2R + 2L)
C57BL/6J	Clozapine	4	4 (2R + 2L)	4 (2R + 2L)	4 (2R + 2L)
	Totals	16	16 (8R + 8L)	16 (8R + 8L)	16 (8R + 8L)

R, right hemisphere; L, left hemisphere, determined at random.

### Mice

All procedures were conducted in strict compliance with US guidelines (National Research Council 1996) and approved by the Institutional Animal Care and Use Committee of the University of North Carolina. We used eight C57BL/6J and eight NOD/ShiLtJ male mice aged 14 weeks. Male mice were studied to minimize estrous effects. All mice were bred at UNC from a colony used to derive the U.S. lines of the Collaborative Cross (Chesler *et al.* 2008). Mice have been bred for four and six generations at the University of North Caroline and have been separated for 6 years from the production colony at The Jackson Laboratory. Animals were weaned at 3 weeks of age and singly housed until the completion of the study. The housing room was on a 12-hr light:12-hr dark schedule with lights on at 7:00 AM and temperature maintained at 24° with 40% to 50% relative humidity. Mice were housed in standard 20 cm × 30 cm ventilated polycarbonate cages with laboratory grade Bed-O-Cob bedding. Water and Purina ProLab IsoPro 3000 were available *ad libitum*.

### Acute clozapine exposure

Animals were transferred from the animal facility to the laboratory and given a 2-hour period to adapt to the new environment before commencing drug treatment. Beginning at 10:00 AM, in randomized order, one mouse every 10 min was administered either 4 mg/kg clozapine or vehicle (0.9% saline) via intraperitoneal injection. The clozapine dose was chosen based on the behavioral phenotyping literature (Duncan *et al.* 2006; Grauer *et al.* 2009; Porter and Prus 2009) and because it had already been shown significantly to alter the cortical transcriptome of C57BL/6J mice in a pilot study. Four milligrams of clozapine powder (Sigma-Aldrich) was dissolved in 100 μl of 20% glacial acetic acid, diluted with 9.875 ml of 0.9% saline, neutralized with 25 μl of 10M NaOH, and injected at a volume of 10 ml/kg. Mice were killed 3 hours after injection by cervical dislocation without anesthesia and brains (with olfactory bulbs attached) were immediately removed.

### Brain dissection

Brains were chilled for 30 sec in RNase-free 1× phosphate-buffered saline and placed upright on a chilled aluminum block for dissection. First, the cerebellum was removed and discarded by peeling back the cerebellar hemispheres and cutting the cerebellar peduncles with microscissors (supporting information, Figure S1). Second, the remaining brain was divided into left and right hemispheres with a fine razor blade. Third, the hindbrain was removed by placing the tissue on its side with the midline facing up, and wedging one end of curved forceps (Fine Science Tools, part number FST 11370-31) into the natural division between these brain regions (Figure S2). The forceps were then closed, which acted to scoop out the hindbrain while leaving the underlying cortex intact. For each mouse, the forebrain and hindbrain from one hemisphere and the intact other hemisphere were collected, weighed, snap frozen in liquid nitrogen and stored at −80° until RNA preparation. Whether the hemisphere was used intact or dissected into forebrain and hindbrain was determined at random. These dissections yielded a total of 48 tissues for microarray analysis.

### RNA extraction

Total RNA was isolated from frozen brains using a Biopulverizer (Biospec). Pulverized samples were placed in tubes containing 1 ml of Trizol reagent (Invitrogen) and a stainless-steel bead. Samples were shaken for 5 min on a Tissuelyzer apparatus (Mixer Mill 300; QIAGEN) and incubated at room temperature for 5 min. We added 200 μl of chloroform (Fisher) and then samples were sequentially shaken for 15 sec on a Tissuelyzer, incubated at room temperature for 3 min, and centrifuged at 10,000 rpm for 15 min at 4°. The supernatant was transferred to a gDNA Eliminator spin column (RNeasy plus mini kit; QIAGEN) and processed according to the manufacturer instructions. RNA was suspended in RNase-free water. The concentration and purity of each sample was determined by spectrophotometer (ND1000; Nanodrop) and confirmed by Microchip Gel Electrophoresis (Agilent), using Agilent 2100 Bioanalyzer and RNA 6000 Nano Chip according to the manufactures’ instruction. RNA integrity numbers of all samples were > 7. RNA samples were stored at -80° until use.

Transcriptional profiling was performed using Affymetrix Gene expression microarrays. Gene expression profiles were generated twice for each sample using standard cartridge format (mouse gene 1.0ST arrays, samples are hybridized separately over 2 days) and using the newer peg format arrays (mouse gene 1.1S T arrays, samples processed on two arrays of 24 samples each). Cartridge arrays were processed at the UNC Functional Genomics Core and peg arrays at the Microarray facility at the University of Pennsylvania. All samples were processed with the GeneChip WT terminal labeling and hybridization protocol in conjunction with the Ambion WT expression kit according the manufacturers instructions. We verified that the probe content was essentially identical between the 1.0ST and 1.1ST arrays.

### Statistical analysis

Normalized expression levels of 35,556 transcripts were estimated using robust multiarray average method (Irizarry *et al.* 2003) implemented in Affymetrix gene expression console with default settings (median polish and sketch-quantile normalization). A total of 6546 positive or negative control transcripts, which do not have mRNA annotation, and additional 700 transcripts without mRNA annotation were dropped. The remaining 28,310 transcripts were used in the following analysis.

We used hierarchical clustering and principal component analysis (PCA) to evaluate overall array performance. The 48 samples were clustered using the R function *hclust* with the average link function. The distance function used in clustering is defined as follows. Let *s*_1_ and *s*_2_ be the expression profiles (across 28,310 transcripts) of two samples. The distance between *s*_1_ and *s*_2_ is defined as 1 − |corr($s_{1}$, $s_{2}$)|. PCA was conducted using the approach of Price *et al.* (2006).

A linear mixed effect model was used to jointly analyze the 48 samples, while accounting for the correlations between three brain regions of each mouse. Let **y** be the expression of one gene in *n* samples, $y={\left(y_{1},\;y_{2},\ldots ,y_{n}\right)}^{T}$. The linear mixed effect can be written as:

(1) y=Xβ+Zu+ε

where ***X***β and **Z*u*** indicate the contributions from fixed effect **X** and random effect **Z**, and ε indicates the residual vector. Fixed effects include brain region (forebrain, hindbrain, and whole brain), strain (C57BL/6J *vs.* NOD/ShiLtJ), acute antipsychotic drug exposure (vehicle *vs.* clozapine), array processing batch (two levels), and two-way interactions between brain region, strain, and drug exposure. The random effect includes a random intercept to account for the dependencies among three brain regions of one mouse, which is equivalent to assuming a compound symmetric structure of the 3 × 3 covariance matrix for each mouse (*i.e.,* the expression of one gene has the same variance for the three brain regions and the same covariance for any pair of brain regions). One may wish to relax these assumptions to more general covariance structures; however, both simulations and inclusion of spurious variable in real data show inflation of type I error for any more complicated model (results not shown).

Initial inspection of the results from the linear mixed effect model revealed conservative *P*-value distribution for some factors, implying the existence of unaccounted confounding variables (Figure S3). Surrogate variable analysis (Leek and Storey 2007) was used to address this problem. First, we applied PCA to the residuals of the initial linear mixed effect models. The first five PCs for each array platform were chosen as initial surrogate variables since they either explained a large proportion of residual variance or explained a moderate amount of variance and were correlated between platforms (Figure S4). Next, the top 1000 genes that were most highly correlated with each initial surrogate variable (in terms of the absolute value of correlation) were selected and the refined surrogate variable was calculated as the first PC of these 1000 genes. Finally, the five refined surrogate variables were included into the linear mixed effect models. The surrogate variables removed the conservative pattern of *P*-value distributions. The models with surrogate variables showed that the interactions between drug and brain regions, and between drug and strain were not significant and hence these terms were dropped. For either array platform, the final model is:

(2) $$\begin{array}{l} y=\beta_{0}+\beta_{1}\text{batch}+\beta_{2}\text{drug}+\beta_{3}\text{strain}+\beta_{4}\text{wb}+\beta_{5}\text{hb}\\ \;\;+\beta_{6}\text{wb}\times \text{strain}+\beta_{7}\text{hb}\times \text{strain}+\sum_{j=1}^{5}\gamma_{j}sv_{j}+Zu+\varepsilon \end{array}$$

where “batch,” “drug,” and “strain” are indicators of second batch, acute antipsychotic exposure, and strain NOD/ShiLtJ, respectively. wb and hb are indicators of whole brain and hindbrain, and sv_*i*_ is the *j*-th surrogate variable.

Using on the aforementioned linear mixed effect model, *P*-values were calculated for each factor across all the 28,310 transcripts. We had also fitted linear fixed effect models for each transcript in each brain region. In general, linear fixed effect models provided consistent differential expression evidence as linear mixed effect models but were less powerful since only 16 samples were analyzed each time in contrast to 48 samples used in linear mixed effect models (see Figure S7b for an example).

Multiple testing across multiple transcripts were corrected to control the false discovery rate (FDR) (Storey and Tibshirani 2003). The proportion of equivalently expressed transcripts (π_0_) was calculated as twice of the proportion of transcripts with *P*-values > 0.5. For a *P*-value cutoff *p*, the expected number of false discoveries (FD) was calculated as π_0_*Np* (N is the total number of transcripts). The number of discoveries (D) was the number of transcripts with *P*-values < *p*. Finally, FDR was calculated as FD/D.

### Functional category analysis

We used SAFE (Barry *et al.* 2005) for functional category_analysis of 2284 Gene Ontology (GO) terms (including 1581 biological process, 269 cellular component, and 434 molecular function categories) and 157 KEGG pathways. GO and pathway information were obtained from R/bioconductor packages mogene10sttranscriptcluster.db. For each factor of interest (*i.e.,* brain region, strain, and drug), we sought to identify the functional category enriched with the differentially expressed transcripts. The association between each functional category and a factor of interest was assessed by Wilcoxon rank-sum test, and a permutation *P*-value was calculated by comparing the Wilcoxon test statistic in the original data *vs.* the Wilcoxon test statistics from the permuted data. Permutation *P*-value cutoff across multiple functional categories was chosen by controlling FDR, and FDR was calculated similarly as the case for differential expression.

Next we briefly describe the calculation of Wilcoxon test statistics for original data and permuted data. A large number of permutations are needed to assess the statistical significance of enrichment (Barry *et al.* 2005), which renders linear mixed effect models computationally infeasible. Therefore, we employed linear fixed effect models. To assess the significance of drug or strain effects, we separately fit a linear fixed effect model for each of the three brain regions:

(3) $$y=a_{0}+a_{1}\times \text{batch+}a_{2}\times \text{drug+}a_{3}\times \text{strain}+\sum_{j\text{=1}}^{\text{5}}b_{j}\times {\text{sv}}_{j}+\varepsilon$$

The significance of drug and strain was measured by the *P*-value for hypothesis tests $a_{1}=0$ and $a_{2}=0$, respectively. Wilcoxon test statistics were then calculated on the basis of the ranks of *P*-values of the 28,310 transcripts. Next, we calculated Wilcoxon test statistics for permuted data. Specifically, we shuffled the labels of drug so that four of eight mice treated with clozapine/vehicle were assigned the label of vehicle/clozapine, and thus obtained $C_{4}^{8}\times C_{4}^{8}=4900$ permutations. Wilcoxon test statistics were then calculated for each permutation. Similarly, Wilcoxon test statistics of strain effect were calculated for 4900 permutations.

Let (*u*, *v*) be any pair of brain regions, and let the variables with subscript *u* or *v* be subsets of the original variables in particular brain region. We used the following model to test brain region effect.

(4) $$y_{u}-y_{v}=a_{0}\text{+}a_{1}\times ({\text{batch}}_{u}{\text{-batch}}_{v}\text{)+}a_{2}\times \text{strain}+\sum_{j=1}^{5}b_{j}{\text{(sv}}_{ju}{\text{-sv}}_{jv}\text{)}\text{+}\varepsilon$$

Note that the main effects of strain and drug were canceled out in the aforementioned equation. The strain effect left in the model actually captured the interaction between strain and brain region. The significance of the hypothesis test *a*_0_ = 0 reflects the brain region effect. We calculated Wilcoxon test statistic using the ranks of the p-values testing hypothesis *a*_0_ = 0 across 28,310 transcripts. Permuted data were generated by randomly flipping *u* and *v* 5000 times and Wilcoxon test-statistics were calculated for each permutation.

### Local DNA sequence similarity between C57BL/6J and NOD/ShiLtJ

**Figure 1:**
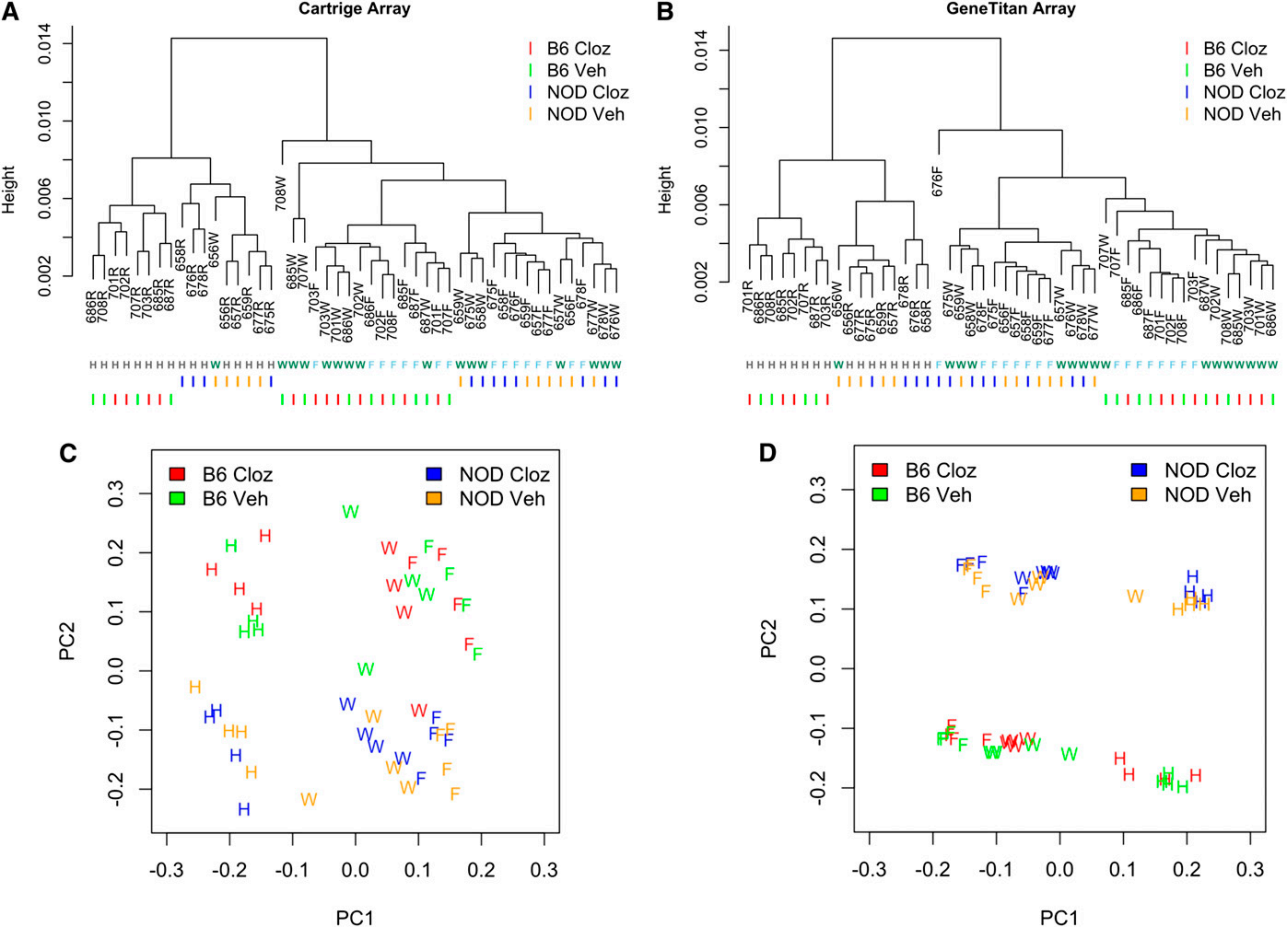
Array-level, unsupervised descriptive summaries of gene expression using hierarchical clustering (A and B) and PCA (C and D) via two Affymetrix assay formats, standard 1.0ST cartridges (A and C) and 1.1ST peg/geneTitan format (B and D). NOD, NOD/ShiLtJ; B6, C57BL/6J mouse strains. Cloz, acute clozapine exposure; Veh, exposure to vehicle without clozapine. Individual mice are indicated by a three-letter code followed by a letter indicating the brain region: W, whole brain; F, forebrain; H, hindbrain.

To determine the level of local sequence similarity between the two inbred strains we used two complementary resources. Initially, we used the local level of sequence similarity estimated based on the analysis of high-density genotype data (Yang *et al.* 2011). This study portioned the genome into 43,285 intervals on the basis of the 4-gamete rule (Collaborative Cross Consortium 2012; Wang *et al.* 2012). Using the whole genome sequence for NOD/ShiLtJ (Keane *et al.* 2011), we re-estimated the sequence similarity within these 43,285 intervals, calculated as the fraction of the base pairs where the two strains have identical genotypes. The lengths of these 43,285 intervals and the distributions of the sequence similarity estimates are illustrated in Figure 1.

## Results

### Global view of gene expression reveals strong brain region and strain effects

**Figure 2:**
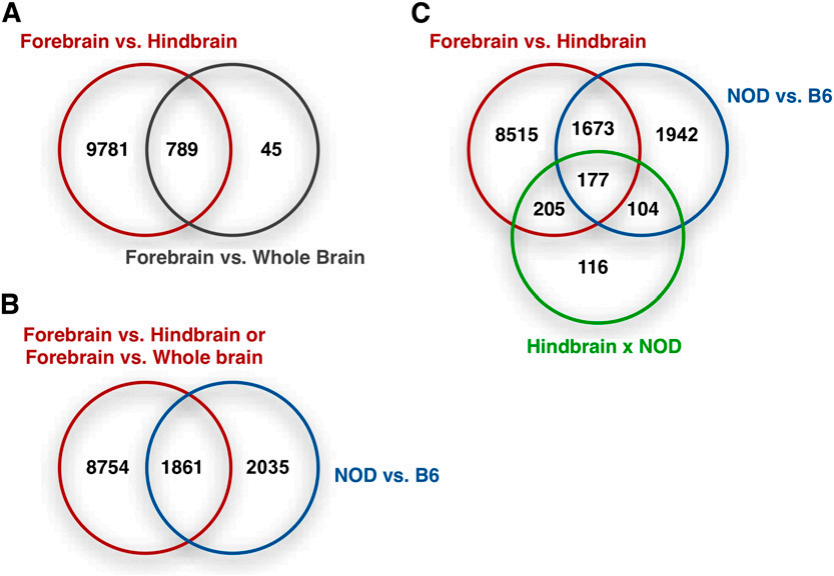
Overlap of the differentially expressed genes (FDR < 0.05) with respect to several experimental factors for 1.1ST arrays. (A) Overlap of the genes differentially expressed between brain regions. (B) Overlap of the genes with brain region effect of hindbrain brain vs. forebrain, strain effect, and/or their interaction. (C) Overlap of genes with any brain region effect and/or strain effect.

Table 1 summarizes the experimental design. All samples were analyzed using the standard Affymetrix 1.0ST cartridge format (one sample per cartridge) and the newer 1.1ST peg format (two runs of 24 samples). High-level descriptions of transcript expression using hierarchical clustering, and PCA clearly indicated large expression differences by strain and brain region (Figure 2, Figure S5, and Figure S6). Overall, expression in forebrain and whole brain were similar and were both different from hindbrain (see patterns of clustering in Figure 2). The first PC, which accounted for approximately 90% of the expression variance, was strongly associated with brain regions. There were also substantial strain differences between C57BL/6J and NOD/ShiLtJ. The effects caused by acute exposure to the antipsychotic clozapine were considerably lesser. The average expression level and standard deviation of the 28,310 transcripts were highly correlated across the two platforms (Figure S8 and Figure S9). To simplify the discussion, we only present the results on the basis of the gene expression data from 1.1ST array. The conclusions based on 1.0ST array, which are similar, are presented in the supplementary materials.

### Differential expression

**Figure 3:**
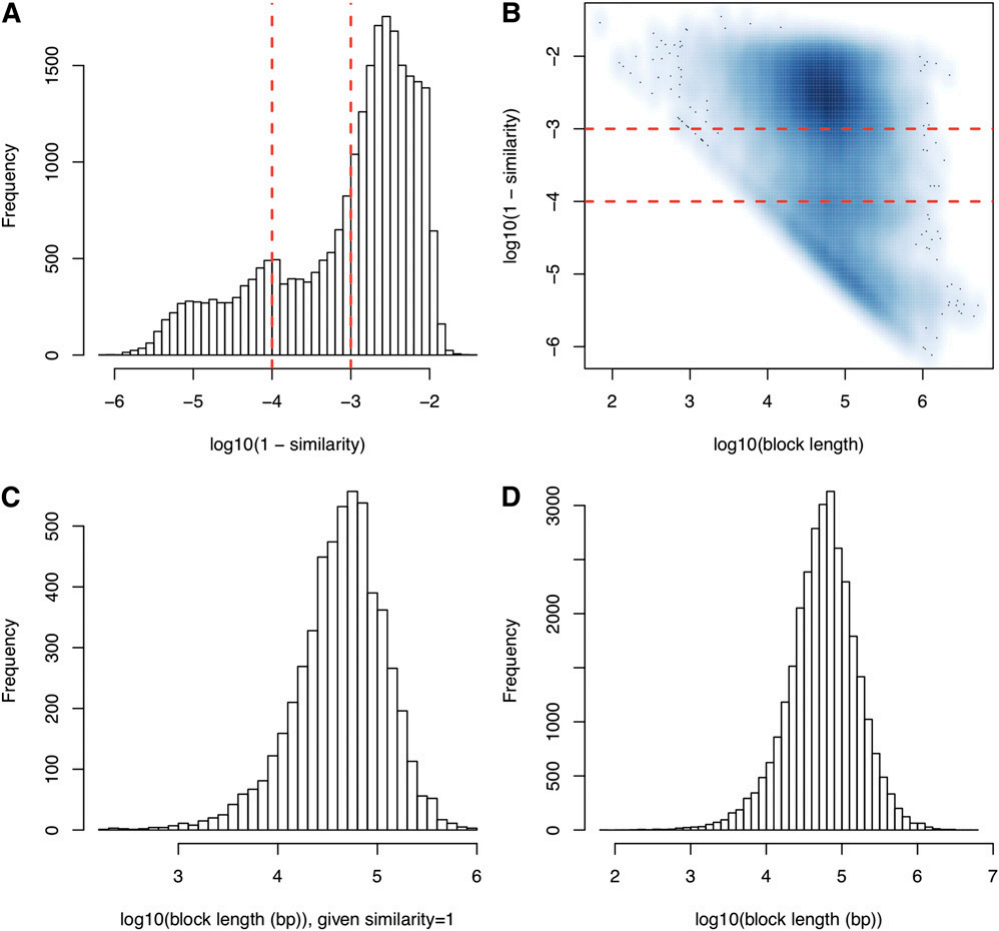
Sequence similarities across blocks. (A) Distribution of sequence similarities given the similarities are smaller than 1. The two vertical lines correspond to sequence similarity of 0.999 and 0.9999, respectively. (B) A smooth scatter plot of log10(block length) and log10(1-similarity). In log10 scale, those blocks with sequence similarity 1 are not shown, which are summarized in (C). (D) Distribution of block lengths.

We applied the linear mixed effect model (equation 2) to each of the 28,310 transcripts. FDRs were calculated along a series of *P*-value cutoffs (Figure 3 and Table S1). For approximately 0.05 FDR, there were 10,570 differentially expressed transcripts for forebrain *vs.* hindbrain, 3896 transcripts for C57BL/6J *vs.* NOD/ShiLtJ, 834 transcripts for whole brain *vs.* forebrain, and none for clozapine *vs.* vehicle. These results were qualitatively similar to Figure 2D, where brain region dominated PC1 and strain dominated PC2. Consistent with the results of hierarchical clustering and PCA, there was considerable similarity between whole brain and forebrain. Furthermore, 94.6% of transcripts (789 of 834) differentially expressed in forebrain *vs.* whole brain were also differentially expressed in forebrain *vs.* hindbrain (Figure 3A), as expected given that whole brain consists of forebrain and hindbrain. Compared with a previous transcriptional atlas (Su *et al.* 2004), differentially expressed genes tended to have higher expression levels in different brain tissues (Figure S10).

When there are large numbers of differentially expressed transcripts, functional category analysis can assist the interpretation of results by identifying larger-scale processes with expression alterations in aggregate. Among the 2284 GO terms (FDR < 0.01), we identified 717 GO terms associated with brain region effect, 41 GO terms for strain effect, and none for drug effect. No GO term was significantly associated with the contrast of forebrain *vs.* whole brain. The 717 brain region−related GO terms arose from the contrast of forebrain *vs.* hindbrain (394 GO terms: 275 BPs, 76 MFs, and 43 CCs) or whole brain *vs.* hindbrain [488 GO terms: 317 Biological Processes (BPs), 88 Molecular Functions (MFs), and 83 Cellular Components (CCs)], with an overlap of 165 GO terms. This overlap is much larger than that expected by chance (Fisher's exact test *P*-value < 2e-16), which again supports the similarity between whole brain and forebrain transcriptomes. The most significant GO terms differentially expressed between forebrain and hindbrain included BP (membrane lipid metabolic process, gliogenesis, nerve−nerve synaptic transmission, and neuron development); MF (transmembrane receptor protein kinase activity and lipid binding); and CC (plasma membrane and postsynaptic membrane). The top 122 GO terms associated with brain regions (*P*-value < 2e-4 in either forebrain *vs.* hindbrain or whole brain *vs.* hindbrain) are listed in Table S3. Strain effects were assessed in forebrain, hindbrain, and whole brain separately for the functional category analysis. Although genetic background altered the expression of thousands of transcripts, only 41 GO terms (20 CCs + 21 MFs, Table S4) were enriched with such transcripts, which suggested the strain signature transcripts were relatively evenly distributed across GO terms. All the 41 GO terms were identified in hindbrain. No GO term was associated with strain effect in either forebrain or whole brain.

**Figure 4:**
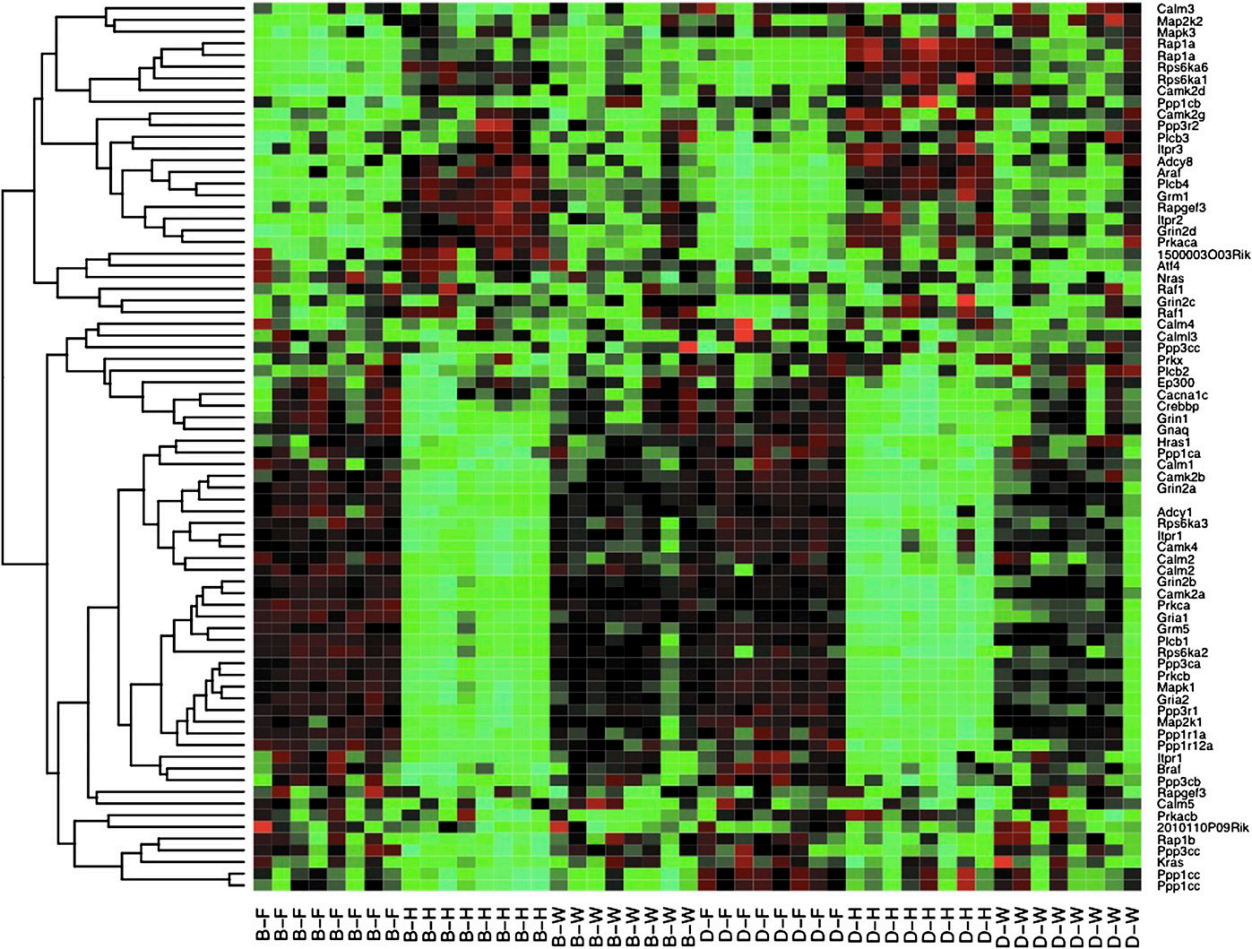
Heat map of the expression of the 77 probesets belonging to pathway “Long-term potentiation.” Each column of the heat map corresponds to a sample and each row corresponds to a probeset. In the column labels, B and D indicates strain of B6 and NOD, respectively, and F, H, and W indicate forebrain, hindbrain, and whole brain, respectively.

Functional category analysis of the 157 KEGG pathways identified no pathway associated whole brain *vs.* forebrain comparison, strain effect, or drug effect. As shown in Table S5, there were 35 pathways significantly associated (FDR < 0.01) with the contrast of whole brain *vs.* hindbrain (21 pathways) and/or forebrain *vs.* hindbrain (21 pathways), with 7 pathways overlap (expect 3, Fisher's exact test *P*-value = 0.01). Interestingly, we identified not only pathways related to crucial behavioral processes (*e.g.* long-term potentiation; Figure 4) and long-term depression (Figure S12), but also cancer-related pathways such as melanoma (Figure S13), and thyroid cancer (Figure S14). A careful examination of genes of melanoma pathway that were differentially expressed between brain regions revealed several growth factors, such as platelet-derived growth factor (*Pdgfd*) and fibroblast growth factors (*Fgf1*, *Fgf16*, *Fgf18*, and *Fgf22*), which suggested that these growth factors are involved in both brain function and tumor growth, although further studies are needed to illuminate the details. In addition, the hindbrain is known to differ from forebrain/whole brain in that it contains nuclei producing melanin (*i.e.,* substantia nigra) (Kim *et al.* 2006) and thyroid-releasing hormone (hypothalamus) (Alkemade *et al.* 2008), though the links to melanoma and thyroid cancer pathways remain unclear.

### The impact of genetic background

A total of 3896 transcripts were differentially expressed between C57BL/6J and NOD/ShiLtJ (in forebrain) using a *P*-value cutoff of 0.01 (FDR 0.048), and 1861 of these transcripts were also differentially expressed between brain regions (Figure 3B). The strain effect was different between forebrain and hindbrain for 778 transcripts (*P*-value 0.002 and FDR 0.057; Figure 3C) but was remarkably similar between whole brain and forebrain for the vast majority of transcripts. This conclusion was confirmed by comparing *P*-values of strain effects assessed in forebrain and whole brain separately using linear fixed effect models (Figure S7a).

The wide variation in local levels of genetic diversity between C57BL/6J and NOD/ShiLtJ provides us with an opportunity to test whether the bulk of strain effects are attributable to local eQTL (gene expression quantitative trait loci), which is commonly referred to as *cis*-eQTL. To test this hypothesis, we grouped transcripts on the basis of the local level of genomic sequence similarity with the expectation that transcripts located in regions with very high sequence similarity between C57BL/6J and NOD/ShiLtJ were less likely to be differentially expressed between two strains. Among the 28,310 transcripts that we considered, 27,081 transcripts had non-missing genomic sequence similarity information (Wang *et al.* 2012; Yang *et al.* 2011). One transcript might have multiple genomic sequence similarity levels (in different regions of this transcript) that were not consistent. We define “consistency” as cases in which there is only one unique sequence similarity measurement or the standard deviation of all sequence similarity measurements is smaller than 0.001.

**Figure 5:**
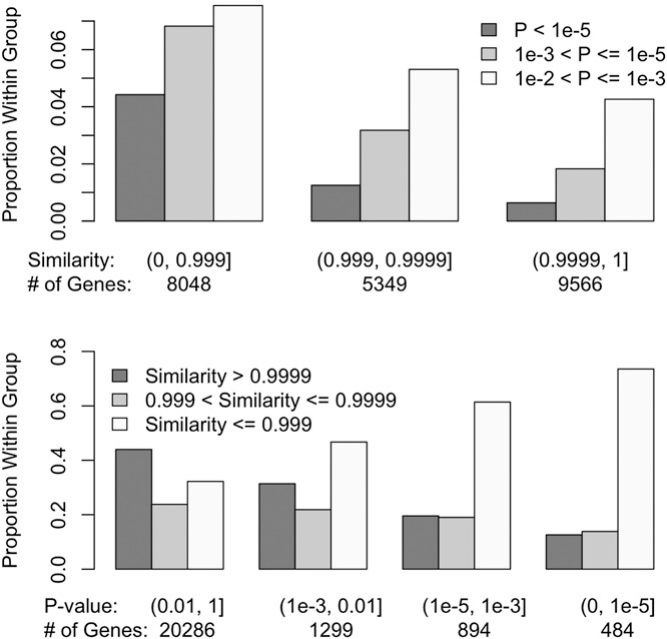
Comparisons of the categories of transcripts on the basis of strain effect *P*-value at forebrain and DNA similarity, for the 22,963 transcripts with consistent genomic sequence similarity estimates between NOD/ShiLtJ and C57BL/6J along the gene body and 10-kb flanking regions. In the upper panel, the transcripts were grouped on the basis of DNA similarity and within each group we compared proportion of transcripts with different *P*-values. In the lower panel, the transcripts were grouped on the basis of the strain effect *P*-values and within each group we compared the proportion of transcripts with different sequence similarities.

**Figure 6:**
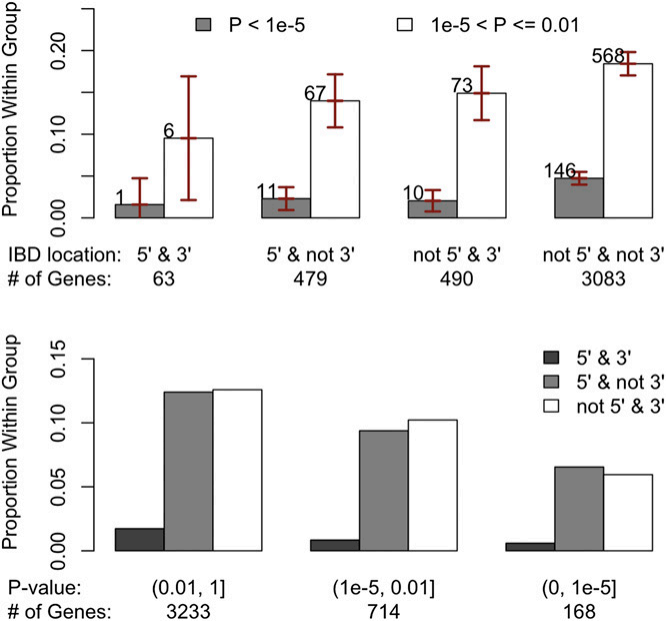
Comparisons of the categories of transcripts based on strain effect *P*-value at forebrain and IBD status at 5′ and 3′ regions for the 4118 transcripts with variable genomic sequence similarity estimates between NOD/ShiLtJ and C57BL/6J in the gene body. Here IBD is defined as genotypic identity > 0.999 using Sanger sequencing data. In the upper panel, the transcripts were grouped on the basis of IBD location and within each group we compared proportion of transcripts within different *P*-value ranges. The vertical bars indicated the 95% confidence intervals. In the lower panel, the transcripts were grouped on the basis of strain effect *P*-values and within each group, we compared the proportion of transcripts with different IBD location statuses.

Among the 27,081 total transcripts analyzed, 22,963 transcripts had consistent sequence similarity measurements along the gene body and 10-kb flanking regions. For these transcripts, we calculated the average genomic sequence similarity over the gene body and flanking regions. The other group includes the remaining 4118 transcripts with inconsistent sequence similarity along the gene body and flanking regions. Transcripts with consistent sequence similarities were significantly less likely to show strain effects in forebrain (Figure 5, *P* < 2.2e^−16^). The effect of variable levels of sequence similarity along chromosomes is shown in Figure S15. We further divided the 4118 transcripts with variable DNA similarity measurements into four groups on the basis of on the identity-by-descent (IBD) status at 5′ and 3′ of the transcript (*i.e.,* as genotype identity >0.999 based on sequencing data). This level of similarity (*i.e.*, 1 SNP per 10 kb) is consistent with previous estimates for recent IBD from a recent ancestor and the error rate of the whole genome sequencing effort (Keane *et al.* 2011). We observed a general trend in which transcripts that were IBD at 5′ and/or 3′ end were significantly less likely to exhibit strain effects in forebrain gene expression than those without IBD (Figure 6, *P* = 1.5e^−4^). Moreover, another group of genes evidenced an interaction between strain and hindbrain effects. Transcripts with uniformly higher DNA similarities were significantly less likely to have strain × hindbrain interaction effects (*P* = 1.3e^−7^, Figure S16). Among transcripts with variable genomic sequence similarity, those IBD at 5′ and/or 3′ end were less likely to exhibit strain × hindbrain interaction effects (Figure S17).

## Discussion

The experiment reported here had two major goals. First, we assessed steady-state transcription in whole brain and two more specific brain regions. Second, we studied the impact of strain genetic background on expression levels. In addition, we also studied the effects of acute clozapine exposure on gene expression in mouse brain.

A critical issue in neuroscience is the region of the brain to study. For many developmental processes and diseases, detailed neuropathological studies have localized the brain region in which to survey the transcriptome. For most neuropsychiatric disorders, however, detailed knowledge of the appropriate brain region to study is lacking or, as with the many psychotropic medications including antipsychotics, the effects might be general across many brain regions. Thus, without more precise knowledge, an investigator might wish to assess transcription in a relatively large region of brain. In mouse, forebrain is often chosen for behavioral and psychopharmacology research given its superficial similarity to human prefrontal cortex. Alternatively, if localization is imprecise, a transcriptional survey might target one hemisphere. We were unable to identify studies that directly addressed this choice.

In direct and careful comparisons, we found considerable similarities between transcription in forebrain and whole brain. This is partly attributable to the fact that “whole brain” consisted of forebrain and hindbrain. Indeed, 94.6% of transcripts differentially expressed in forebrain *vs.* whole brain were also differentially expressed in forebrain *vs.* hindbrain, and no functional categories emerged in comparisons of whole brain *vs.* forebrain.

There were major effects of strain. The strains we studied (C57BL/6J and NOD/ShiLtJ) are superficially quite similar. Both are commonly used classical inbred strains, and over 93% of each genome is of *Mus musculus domesticus* origin (Yang *et al.* 2007). However, this overall comparison is misleading because there are considerable differences between these strains at a regional level (Yang *et al.* 2011). Supporting this contention, approximately 14% of all transcripts were differentially expressed between C57BL/6J and NOD/ShiLtJ in forebrain. Genes with differential expression between strains did not form interpretable pathways, which suggests that strain effects do not result from larger scale processes (*e.g.,* selection) operating on the level of biological processes. We hypothesized that the bulk of strain effects might be attributable to *cis*-eQTL, and then the genes differentially expressed between two strains tend to have lower level of DNA sequence similarity between the two strains. Therefore a functional category is associated with strain effect only if a significant proportion of the genes in this category have lower level of DNA sequence similarity between the two strains, which was not observed in C57BL/6J and NOD/ShiLtJ.

Our results confirmed the association between the DNA sequence similarity between C57BL/6J and NOD/ShiLtJ and differential expression between these two strains. Transcripts with higher DNA similarities between C57BL/6J and NOD/ShiLtJ were much less likely to show strain effects. Moreover, among the 4118 transcripts with varying similarities between strains across their lengths, transcripts that were IBD at 5′ and/or 3′ ends were much less likely to exhibit strain effects. The similar effect of sequence diversity in the 5′ and 3′ of the genes suggest that polymorphisms in both the promoter regions and in the 3′UTRs have similar potential to explain gene expression variation, an important consideration when ascribing SNPs associated with biomedical traits to nearby gene. These analyses also indicate that number and strength of *cis*-eQTL should be higher in populations with high levels of genetic diversity such as the CC and DO (Bottomly *et al.* 2012; Collaborative Cross Consortium 2012; Svenson *et al.* 2012). This result easily explains the high number of *cis*-eQTL identified in experiments using incipient CC lines (Aylor *et al.* 2011; Durrant *et al.* 2011) and suggests that gene expression could be under genetic control in most genes in that population.

Finally, we assessed the impact of acute clozapine exposure on gene expression. In contrast to the robust strain and brain region effects, no transcript or functional categories is significantly associated with acute clozapine exposure. The lack of significance could have been attributable to insufficiently large sample size or technical aspect of clozapine dosing (*e.g.,* insufficient dose or duration of treatment). Intriguingly, four top-ranked biological processes from functional category analyses (hemopoiesis, immune system development, hemopoietic or lymphoid organ development, and myeloid cell differentiation) immediately suggest a connection to clozapine-induced agranulocytosis: a potentially lethal adverse effect of clozapine treatment in humans. Moreover, clozapine-induced weight gain is an additional adverse effect, and multiple relevant biological processes were altered in hindbrain, *e.g.,* regulation of generation of precursor metabolites and energy.

In summary, we provide gene expression data and analytical results of comparing mouse gene expression in three brain regions and two strains. We conclude that gene expression measured in whole brain is highly similar to gene expression measured in forebrain and DNA variations around the gene, especially at 5′ or 3′ regions of the gene have large impact on gene expression.

## Supplementary Material

Supporting Information
