# Supplementary material for: Transcriptome Atlases of Mouse Brain Reveals Differential Expression Across Brain Regions and Genetic Backgrounds
Source: G3 (Bethesda). 2012 Feb 1;2(2):203–11. doi: 10.1534/g3.111.001602 (PMC3284328; doi:10.1534/g3.111.001602)
Supplement: Supporting Information [file supp_2_2_203__index.html]

Supporting Information 

# Transcriptome Atlases of Mouse Brain Reveals Differential Expression Across Brain Regions and Genetic Backgrounds

## Supporting Information for Sun *et al.*, 2012

**Files in this Data Supplement:**

- Supporting Information - Figures S1-S17 and Tables S1-S5 (PDF, 6.1 MB)
- Figure S1 - Removal of cerebellum (PDF, 1 MB)
- Figure S2 - Separation of forebrain from hindbrain (PDF, 338 KB)
- Figure S3 - Conservative p-values from the initial analysis (PDF, 338 KB)
- Figure S4 - Surrogate Variable Analysis (PDF, 587 KB)
- Figure S5 - PCA for gene expression from 1.0ST cartridge arrays (PDF, 545 KB)
- Figure S6 - PCA for gene expression from 1.1ST peg arrays (PDF, 533 KB)
- Figure S7 - Comparison of brain-region-specific analysis and joint analysis (PDF, 320 KB)
- Figure S8 - Comparisons of two platforms (I) (PDF, 435 KB)
- Figure S9 - Comparisons of two platforms (II) (PDF, 294 KB)
- Figure S10 - Comparison with GNF Atlas data (PDF, 533 KB)
- Figure S11 - Comparison of differentially expressed genes in 1.0ST cartridge array (PDF, 533 KB)
- Figure S12 - Heatmap of the expression of pathway "Long-term depression" (PDF, 185 KB)
- Figure S13 - Heatmap of the expression of pathway "Melanoma" (PDF, 185 KB)
- Figure S14 - Heatmap of the expression of pathway "Thyroid cancer" (PDF, 845 KB)
- Figure S15 - Sequence similarity vs. strain effect in forebrain along chromosomes (PDF, 370 KB)
- Figure S16 - Comparison strain x hindbrain interaction effects vs. DNA similarity (PDF, 178 KB)
- Figure S17 - Comparison strain x hindbrain interaction effects vs. DNA similarity at 5' and 3' regions (PDF, 170 KB)
- Table S1 - FDR at different p-value cutoffs for 1.1ST array (PDF, 74 KB)
- Table S2 - FDR at different p-value cutoffs for 1.0ST array (PDF, 67 KB)
- Table S3 - The 122 GO terms associated with brain region effects (PDF, 74 KB)
- Table S4 - The 41 GO terms associated with strain effect in hindbrain (PDF, 58 KB)
- Table S5 - The 35 pathways associated with brain region effect (PDF, 58 KB)
