# Supplementary material for: Transcriptome Atlases of Mouse Brain Reveals Differential Expression Across Brain Regions and Genetic Backgrounds
Source: G3 (Bethesda). 2012 Feb 1;2(2):203–11. doi: 10.1534/g3.111.001602 (PMC3284328; doi:10.1534/g3.111.001602)
Supplement: Supporting Information [file supp_2.2.203_001602SI.pdf]

Cerebellum Dissection

Important Note: The following dissections are performed using the aid of a dissecting microscope, and on filter paper moistened with cold RNAse-free 1X PBS, on top of a petri dish filled with ice. All surgical instruments are cleaned with RNAseZap in between each animal and brain region.

Method:
Cut one side at the cerebellar peduncle (fig.1, black line) with surgical scissors, or by pinching with forceps.

Note: Inferior colliculi have been removed for better visualization of the peduncles.
Removing the inferior colliculi is not necessary for the cerebellum dissection.

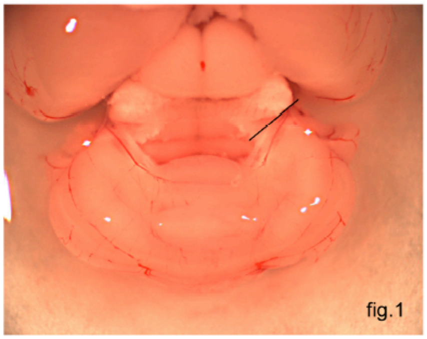

Gently push cerebellum to the side (fig.2).

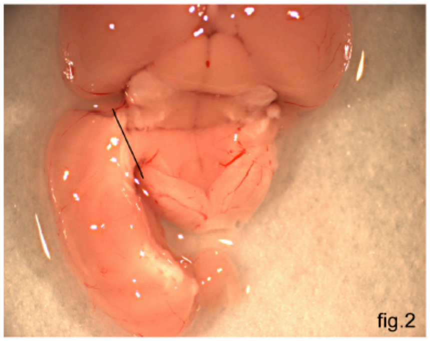

Snip the other cerebellar peduncle (fig.2, black line) to remove the cerebellum (cerebellum is shown removed in fig.3).
Immediately place tissue in the bottom of a 15ml Falcon tube, and immediately snap freeze on dry ice.

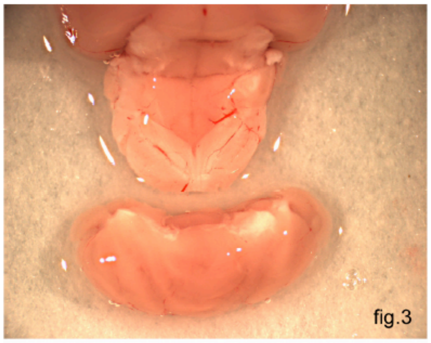

Figure S1 Removal of cerebellum.

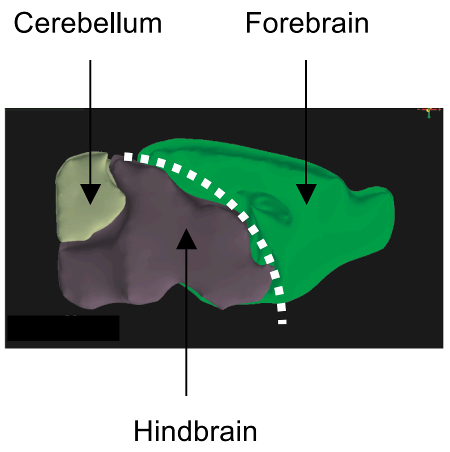

Figure S2 Separation of forebrain from hindbrain. One end of fine, curved forceps was wedged into the natural division between forebrain and hindbrain (dashed white line). The forceps were then closed, which acted to scoop out the hindbrain while leaving the underlying cortex intact.

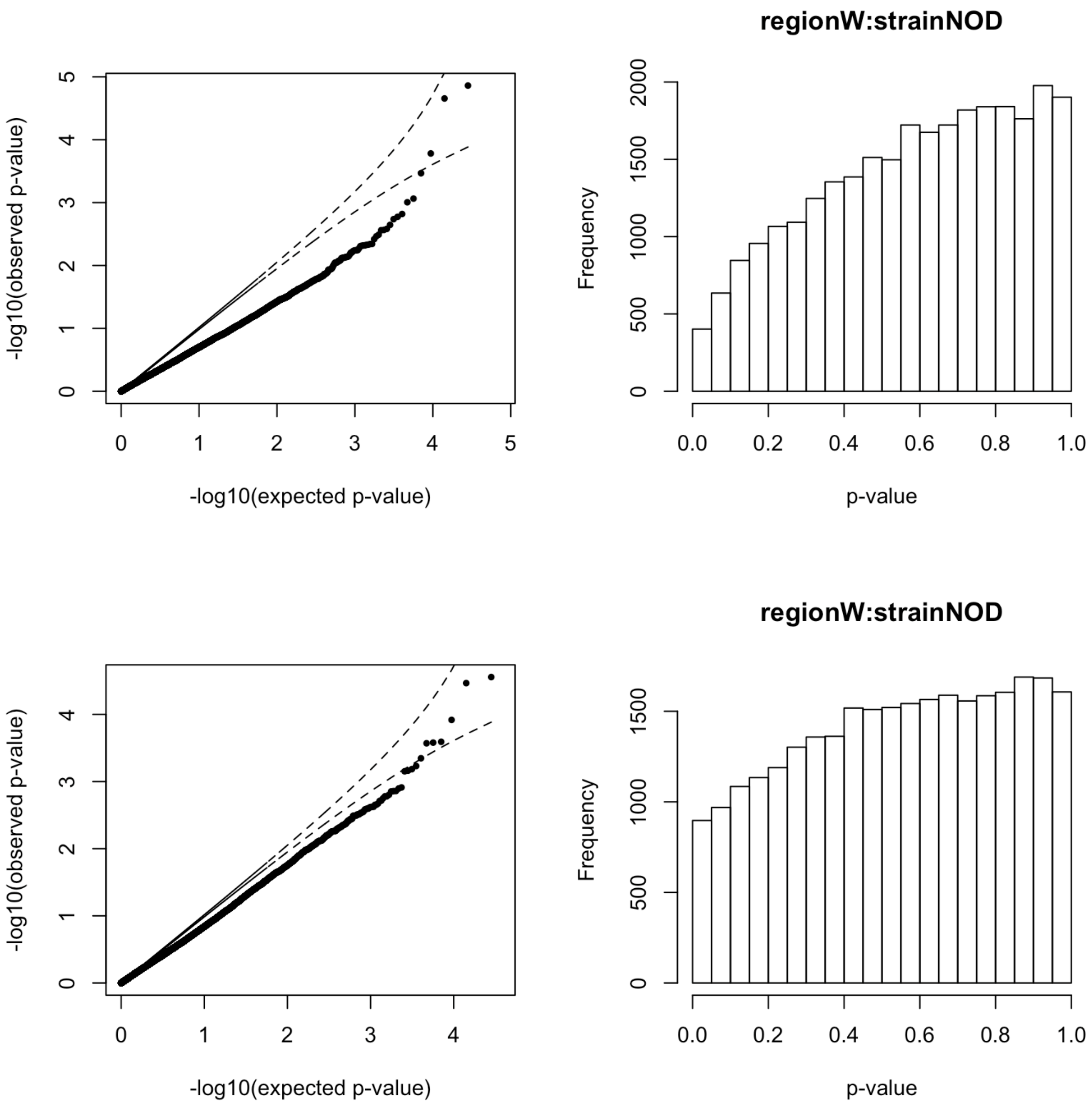

Figure S3 Conservative p-values from the initial analysis. In the initial analysis, we ran a linear mixed effect model for each gene. These figures illustrate a conservative p-value distribution for the interaction between whole brain region indicator and NOD strain indicator for 1.0ST cartridge array (upper panel) and 1.1ST GeneTitan array (lower panel). The left panel shows the QQ-plot of the p-values for the 35,556 genes. The broken lines indicate 95% confidence intervals if p-values follow a uniform distribution. The right panel is the histogram of the p-values. These figures clearly demonstrate conservative patterns, which imply the effects of unaccounted confounding variables.

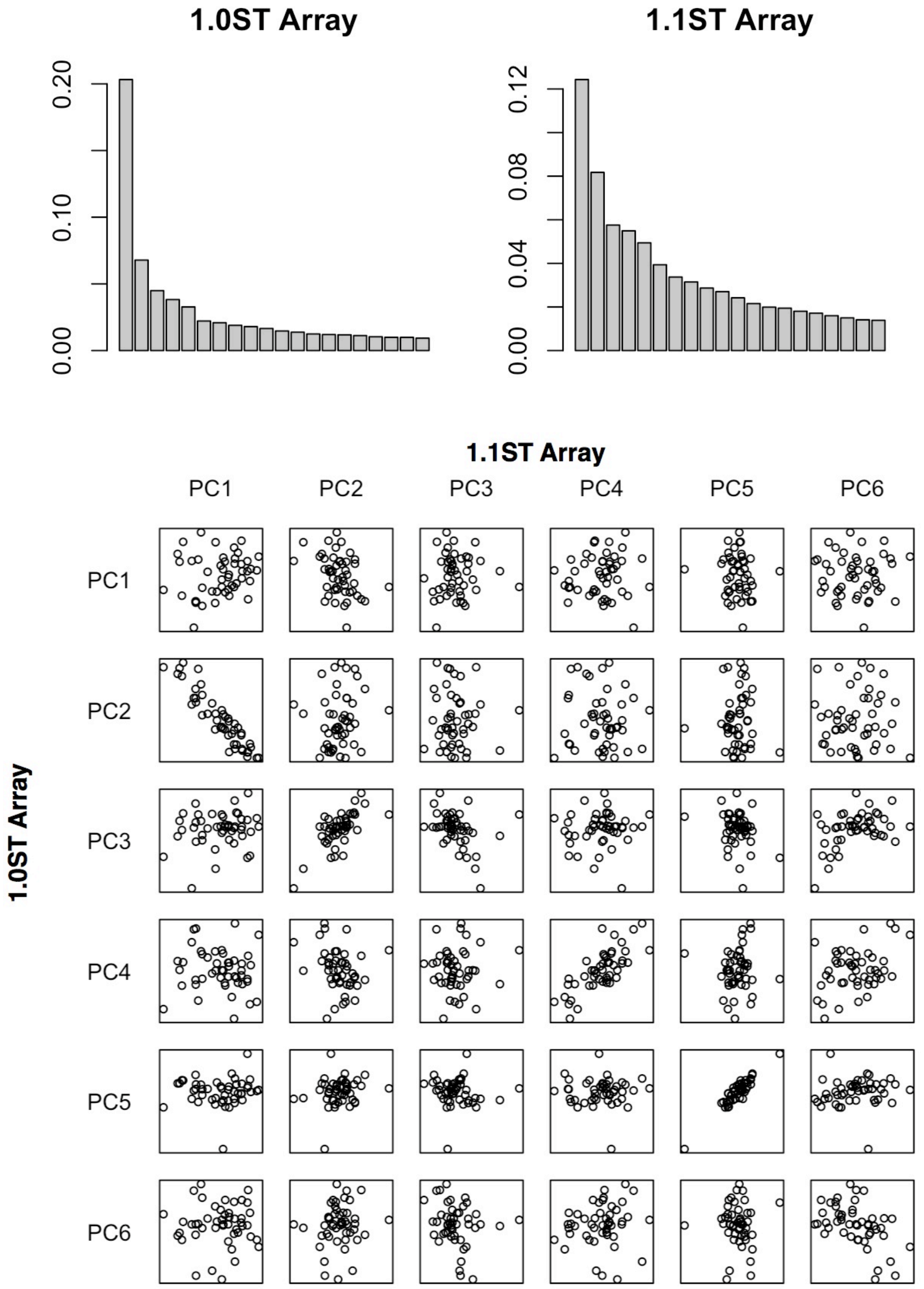

Figure S4 **Surrogate Variable Analysis. (a) The first 20 eigenvalues of the residuals from linear mixed effect model specified in equation (1) in main text Methods section for 1.0ST cartridge data. (b) The first 20 eigenvalues of the residuals of 1.1ST GeneTitan data. (c) Scatter plots of PC1-5 from expression data of the two platforms. GeneTitan PC1 is correlated with cartridge PC2, and PC3 and PC4 of the two platforms are correlated. We chose the first four PCs as surrogate variables since they either have large eigenvalues or are correlated with other PCs in the other platform.**

(a) Eigenvalues and projection plots for the first three PCs.

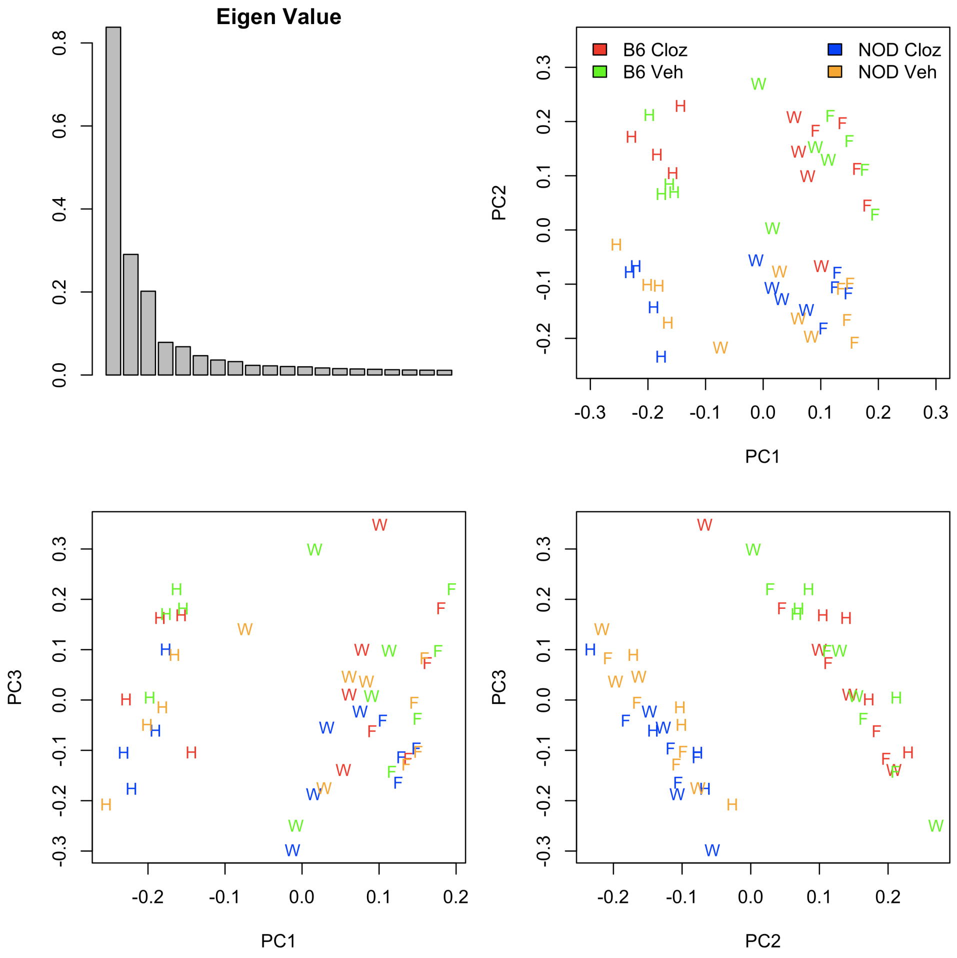

(B) R2 between the five covariates and the first 9 PCs. The covariate left/right indicate the sample is from left or right hemispheres.

	PC 1	PC 2	PC 3	PC 4	PC 5	PC 6	PC 7	PC 8	PC 9
left/right	0	0	0	0.01	0.05	0	0.02	0	0.02
drug	0	0	0.03	0.11	0	0.12	0.12	0.18	0.01
strain	0.01	0.79	0.19	0.01	0	0	0	0	0
region	0.94	0	0.02	0.04	0.07	0.01	0.39	0.02	0.01
day	0.04	0.06	0.02	0	0.42	0	0.02	0.01	0.03

Figure S5 PCA for gene expression from 1.0ST cartridge arrays.

(a) Eigenvalues and projection plots for the first three PCs.

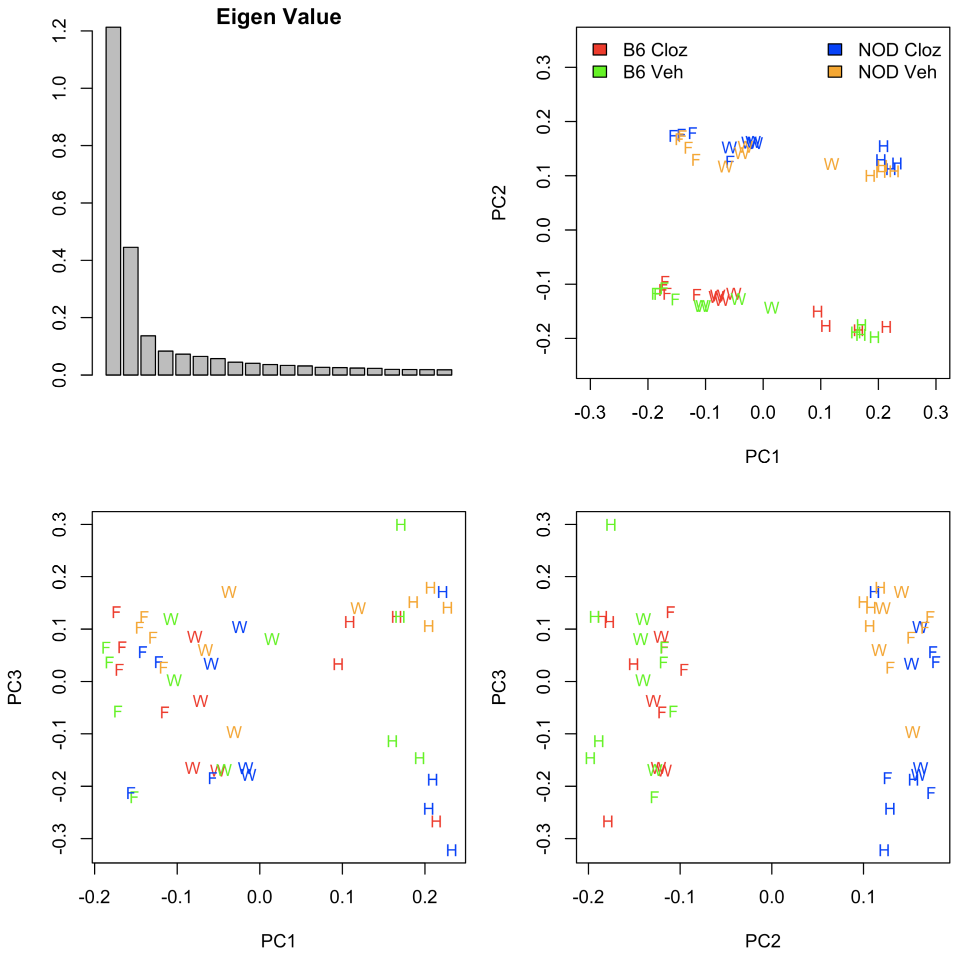

(b) R2 between the five covariates and the first 9 PCs.

	PC 1	PC 2	PC 3	PC 4	PC 5	PC 6	PC 7	PC 8	PC 9
left/right	0	0	0.01	0	0.01	0.03	0	0	0.01
drug	0	0	0.12	0.02	0.06	0.09	0.22	0.08	0.01
strain	0.03	0.96	0	0	0	0	0	0	0
region	0.92	0.02	0	0.13	0.07	0.08	0.1	0.1	0.08
day	0.03	0.04	0.01	0.13	0	0.08	0.02	0.05	0

Figure S6 PCA for gene expression from 1.1ST peg arrays.

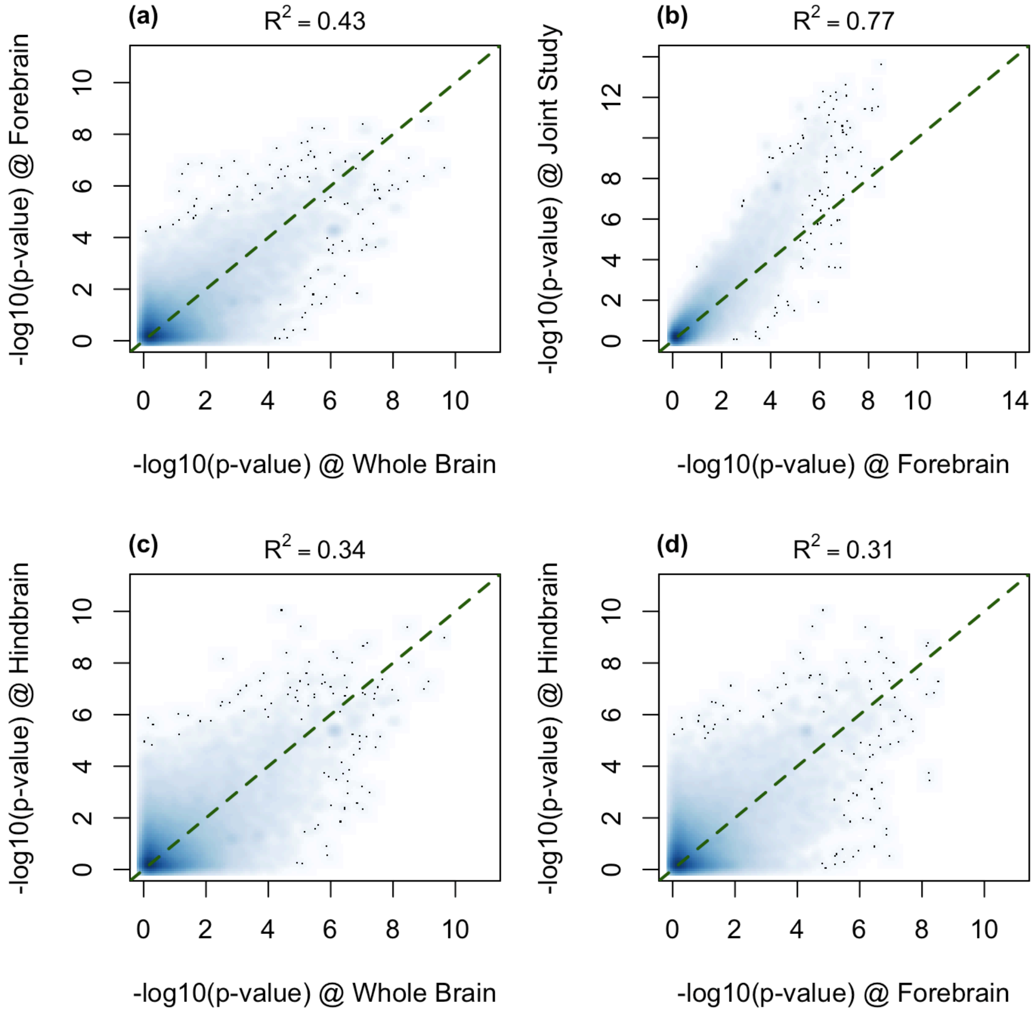

Figure S7 Comparison of brain-region-specific analysis and joint analysis. Comparison of strain effects assessed in different brain regions (by fixed effect model) or assessed by joint modeling three brain regions using linear mixed effect model. The R^2 is calculated as the correlation square of $-\log_{10}$(p-value).

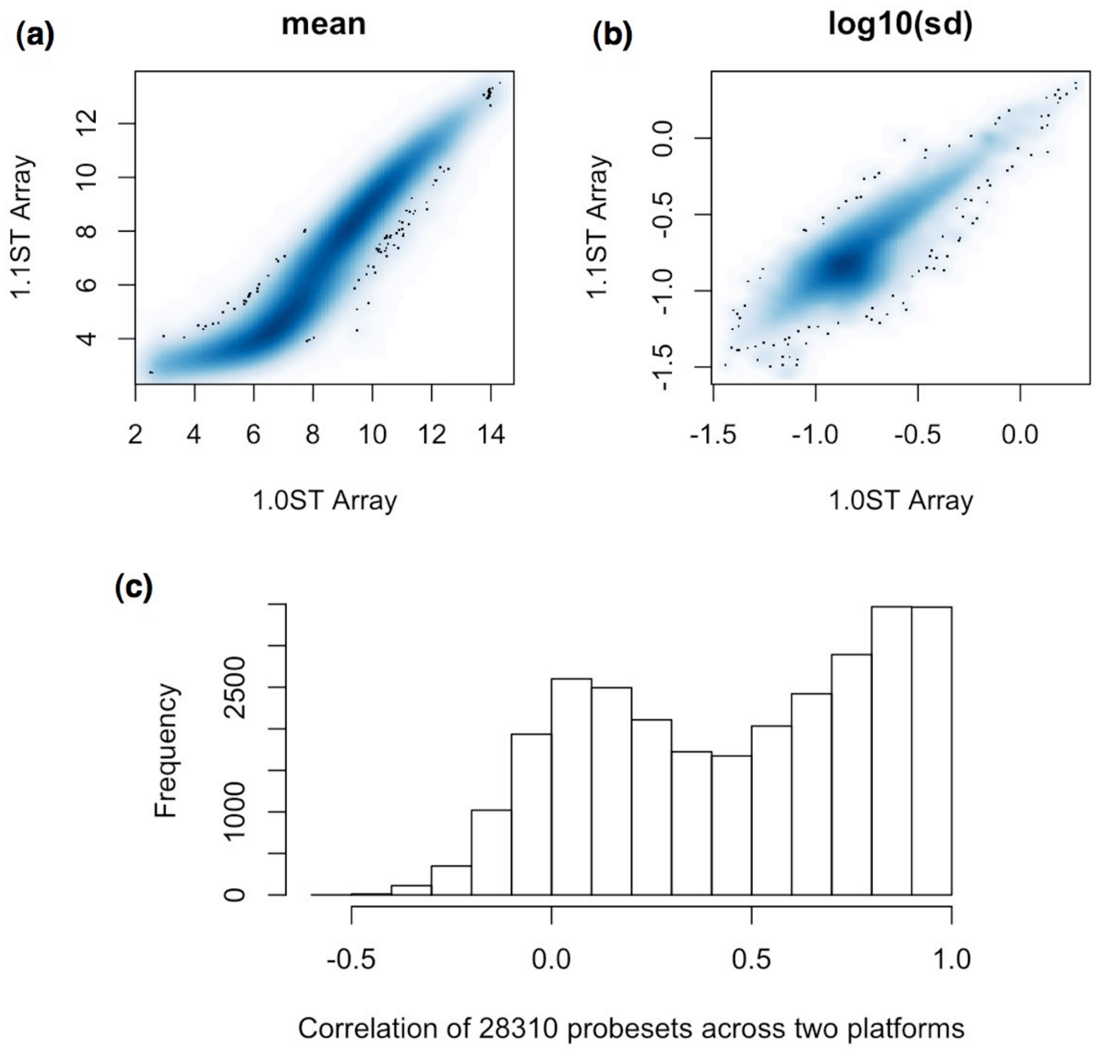

Figure S8 Comparisons of two platforms (I). (a) Scatter plot of the expression means of the 28,310 transcripts across the two platforms. (b) Scatter plot of the expression standard deviations of the 28,310 transcripts across the two platforms. (c) Histogram of the correlations of transcript expression across the two platforms. The correlation of each transcript is calculated as follows. Since there are 48 samples for each platform, the expression data of one transcript across the two platforms are two vectors of length 48, and the correlations of these two vectors were calculated.

(a) Cross-platform correlations and mean expression level and standard deviations.

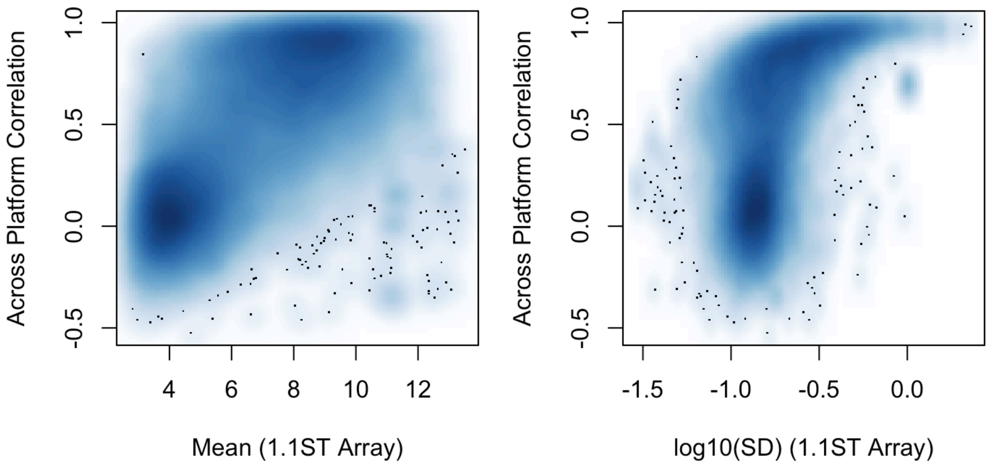

(b) The overlap of the differentially expressed genes across the two platforms.

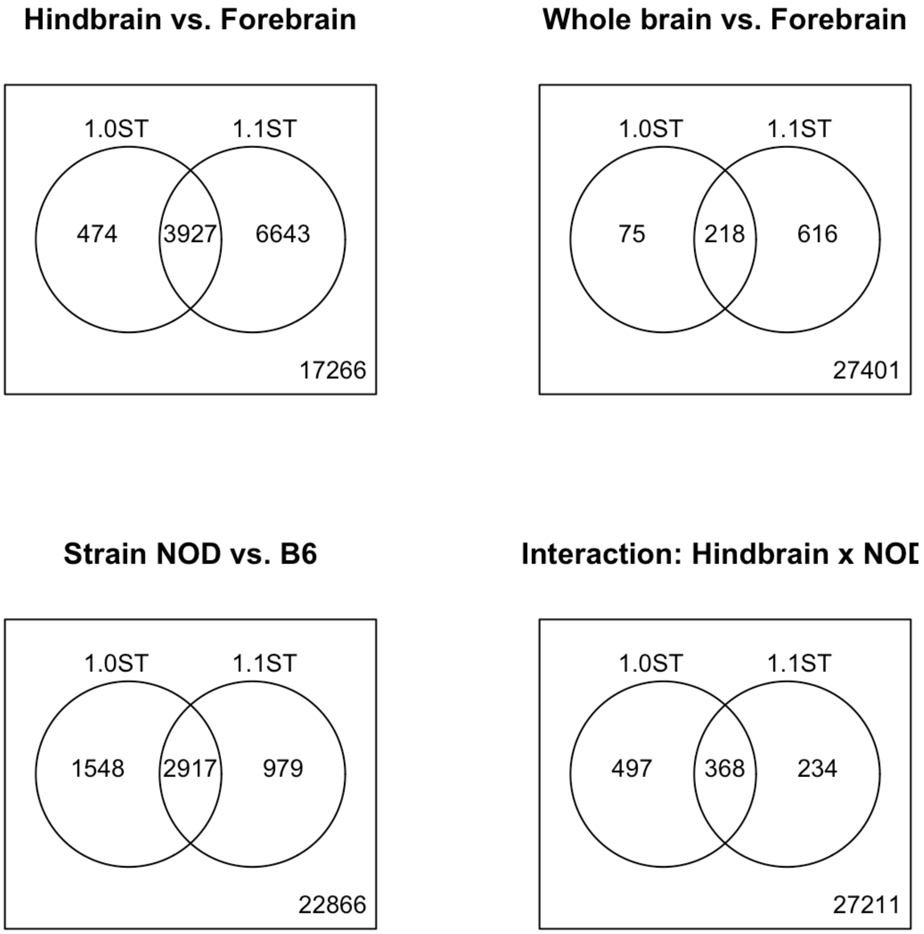

Figure S9 Comparisons of two platforms (II).

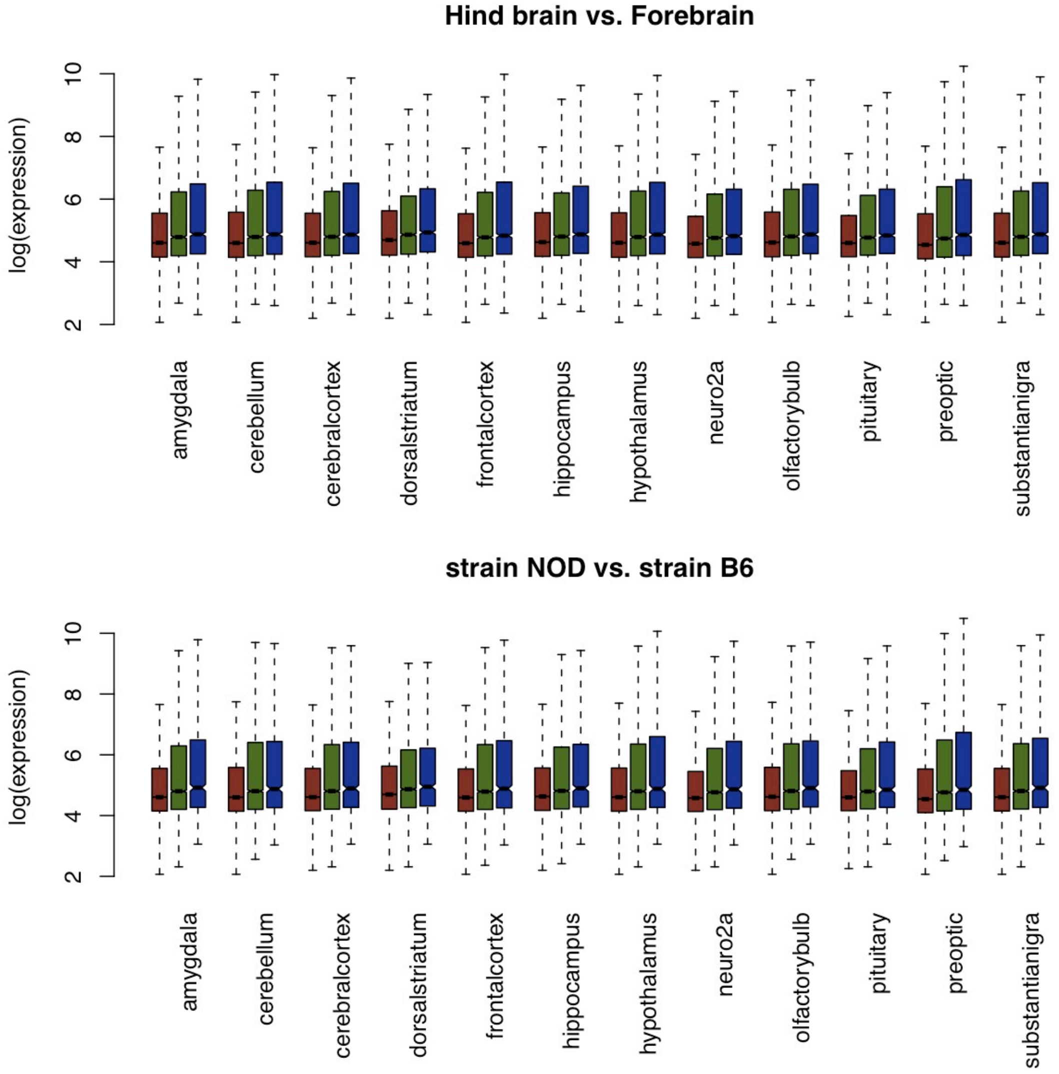

Figure S10 Comparison with GNF Atlas data. GNF atlas gene expression and array annotation (GNF1M) were downloaded from http://biogps.org/downloads/. The expressions of two replicates of each brain tissue were log transformed and then averaged. Probe sets of 1.1ST array and GNF 1M array were matched through NCBI refseq IDs. The red boxplots were the expression from all probe sets in the GNF 1M array. In the upper panel, the green and blue boxplots showed the expression of the genes in GNF1M arrays that were equivalently expressed (p-value > 0.1) or differentially expressed (p-value < 0.001) between forebrain and hindbrain in 1.1ST arrays. In the lower panel, the green and blue boxplots showed the expression of the genes in GNF1M arrays that were equivalently expressed (p-value > 0.1) or differentially expressed (p-value < 0.001) between two strains in the 1.1ST arrays. Note equivalently expressed genes have higher expression level in GNF 1M array partly because genes that can be mapped by refseq IDs have higher expression level.

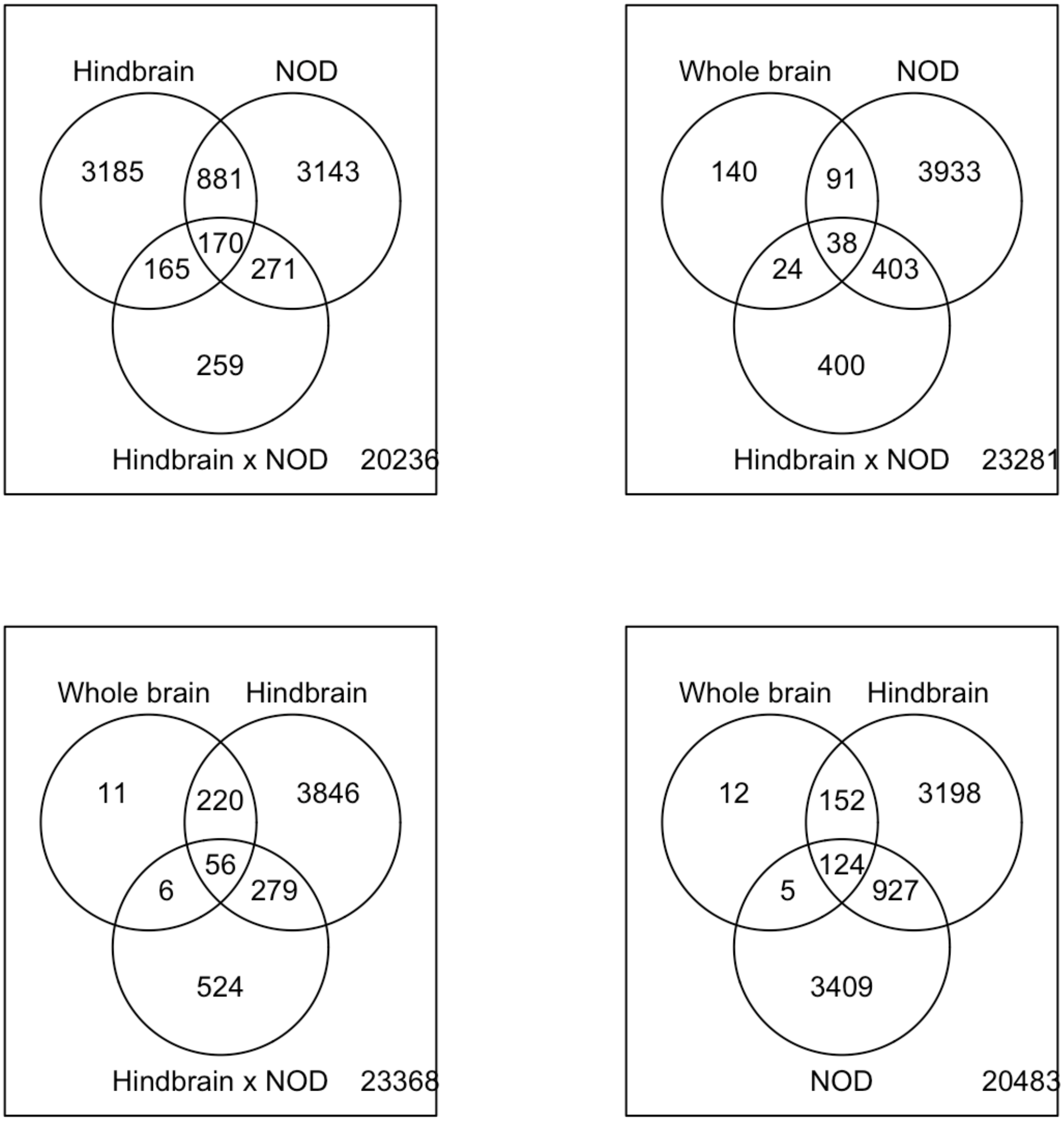

Figure S11 Comparison of differentially expressed genes in 1.0ST cartridge array. This Venn diagram is based on the results from linear mixed effect model for all the 48 samples and p-value cutoff for differential expression is decided by FDR < 0.05. Circles labeled by Hindbrain/Whole brain describe the differential expression between forebrain and Hindbrain/ Whole brain, circles labeled by NOD describe the differential expression between strains B6 and NOD, and Hindbrain x NOD indicates interaction term between Hindbrain indicator and NOD strain indicator.

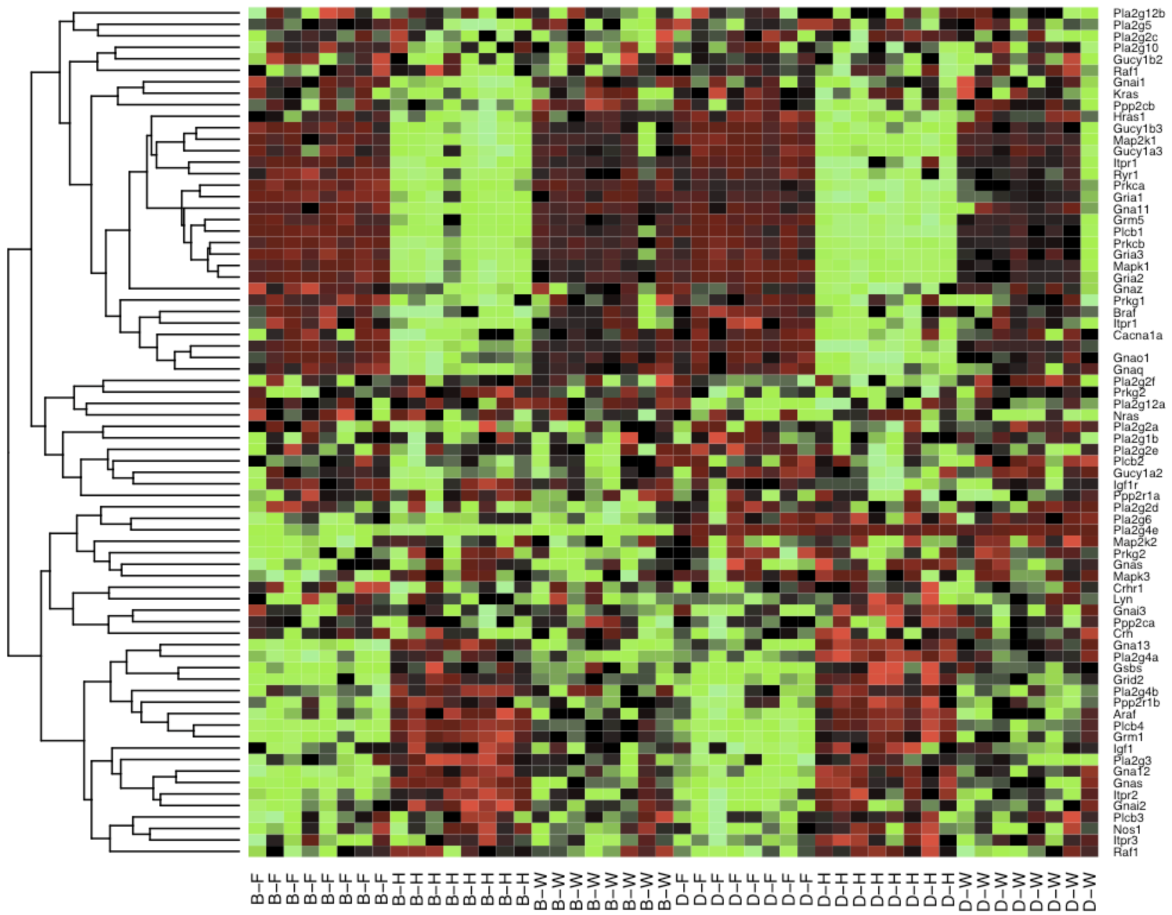

Figure S12 Heatmap of the expression of pathway "Long-term depression". Heatmap of the expression of the 76 probesets belonging to pathway "Long-term depression". Each column of the heatmap corresponds to a sample and each row corresponds to a probeset. In the column labels, B and D indicates strain of B6 and NOD respectively, and F, H, and W indicate forebrain, hindbrain and whole brain, respectively.

Figure S13: Heatmap of the expression of pathway “Melanoma”.

Figure S13 is available for download as a PDF at http://www.g3journal.org/lookup/suppl/doi:10.1534/g3.111.001602/-/DC1.

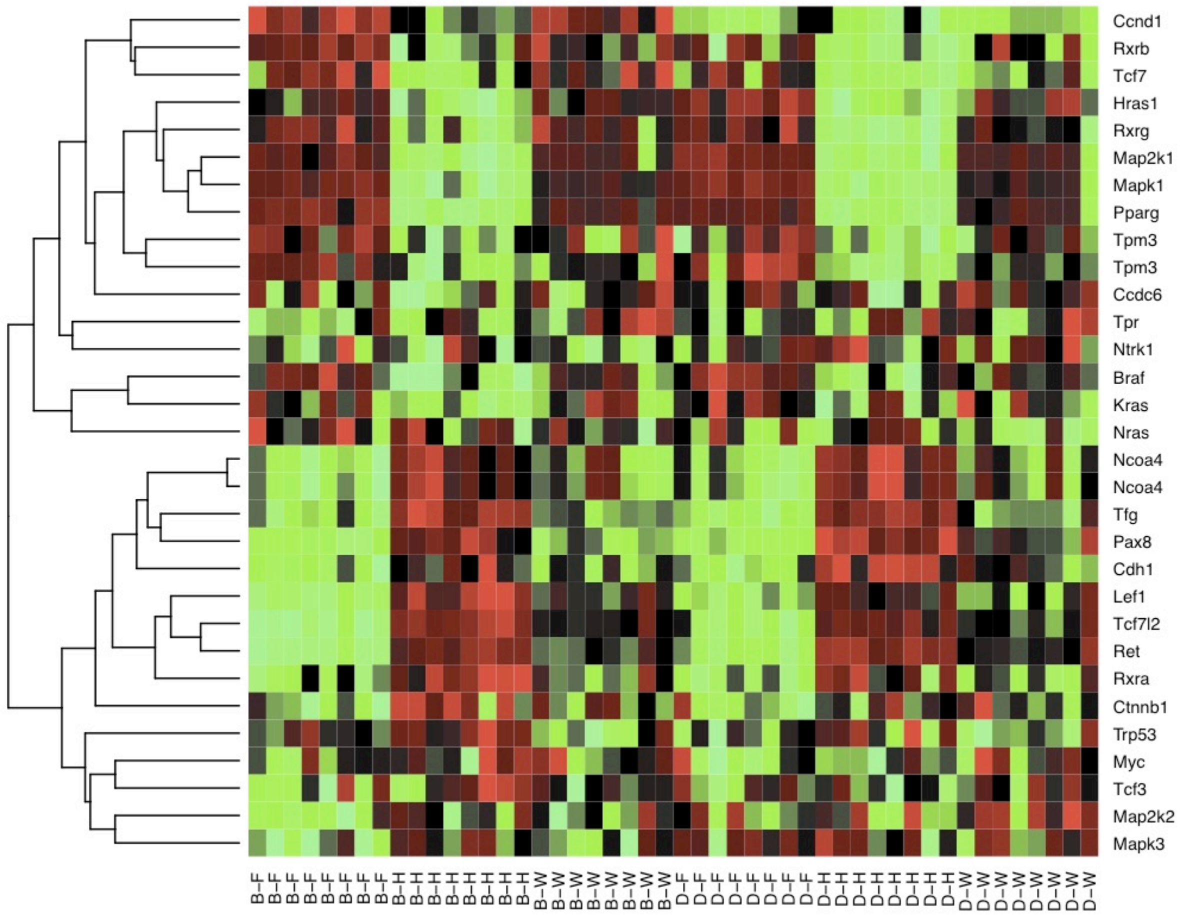

Figure S14 Heatmap of the expression of pathway "Thyroid cancer".

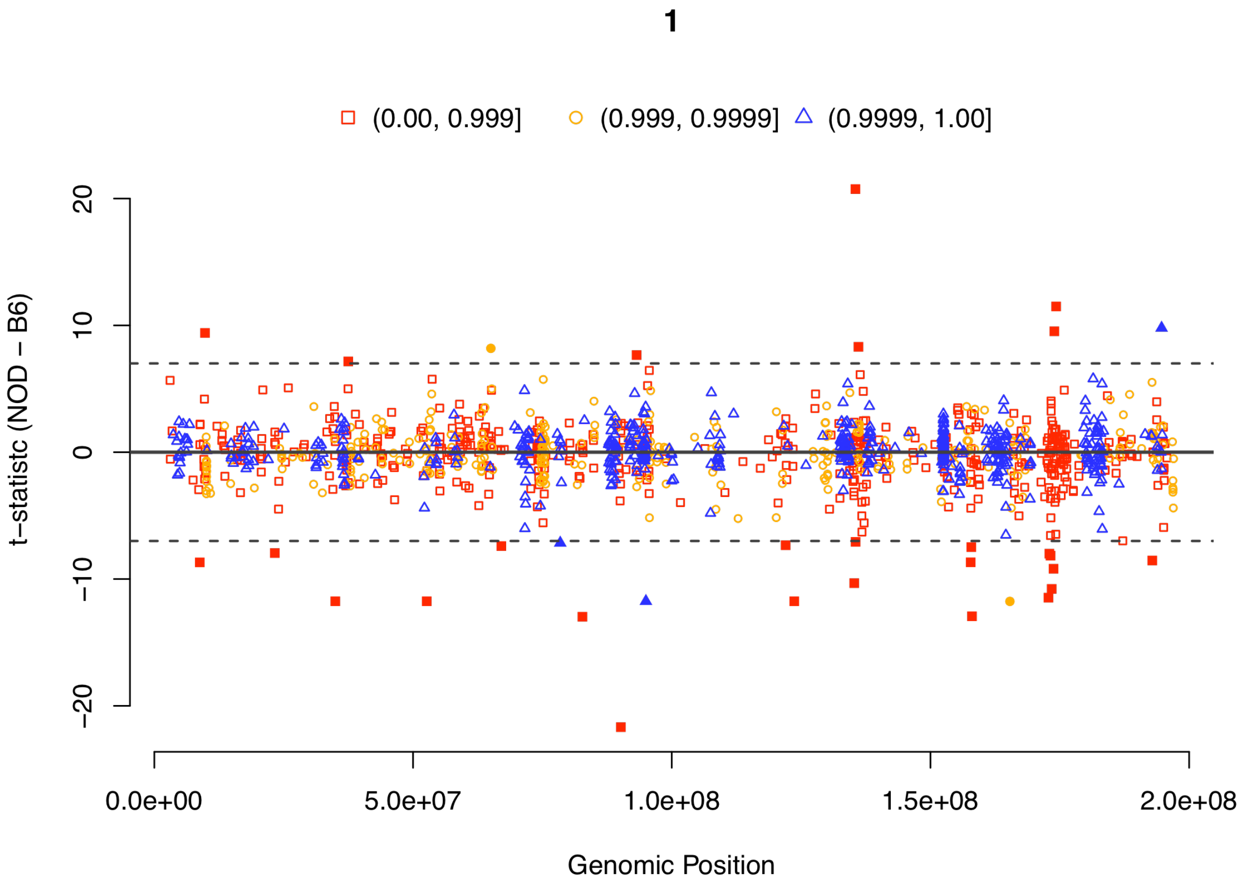

Figure S15 Sequence similarity vs. strain effect in forebrain along chromosomes. This figure shows the relation between strain effect in forebrain and variable levels of sequence similarities along chromosome 1. The X-axis is genomic position by base pair and the Y-axis is the t-statistic comparing the expression in NOD/ShiLtJ vs. C57BL/6J so that positive t-statistics indicate higher expression in NOD. One point indicates one of the 22,963 transcripts with consistent sequence similarity measurement. Different color (as labeled at the top of the figure) indicates different level of sequence similarity. Two horizontal broken lines indicate t-statistics of 7 or -7, which approximately corresponds to p-value 1e-5, the cutoff used in Figure 5 of the main text. Those transcripts with t-statistics beyond these cutoffs are labeled by filled points. Similar figures for other chromosomes can be found in File S2.

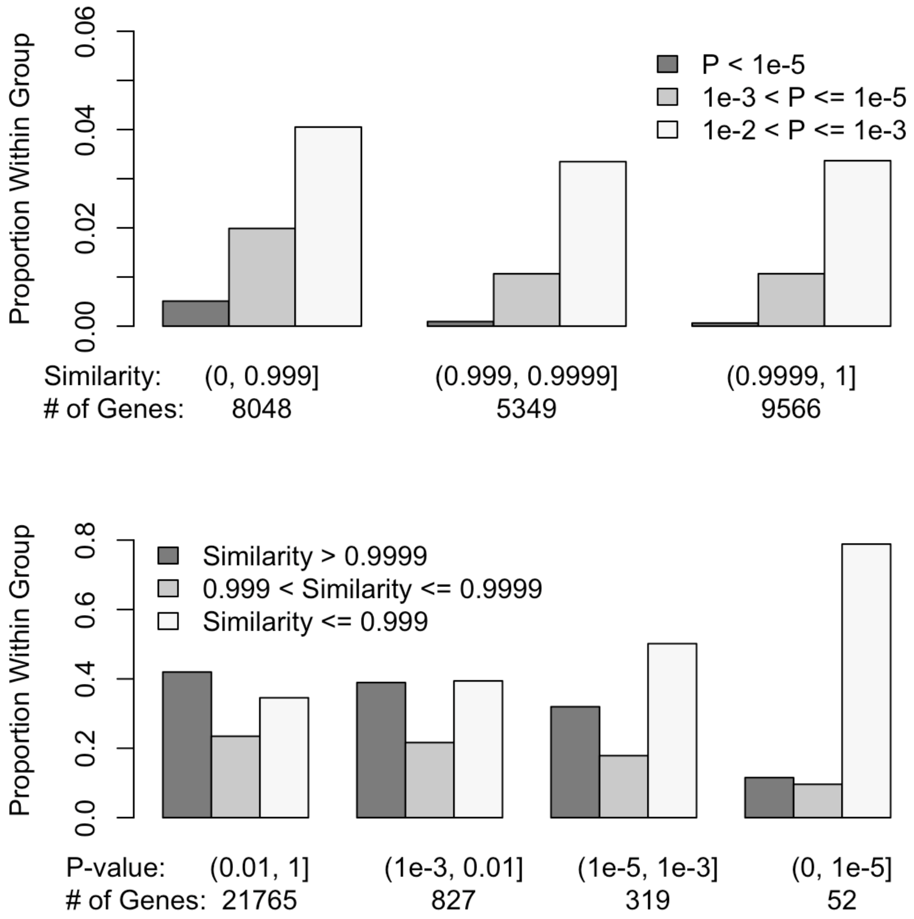

Figure S16 Comparison strain x hindbrain interaction effects vs. DNA similarity. Comparisons of the categories of transcripts based on p-value of strain x hindbrain interaction and DNA similarity, for the 22,963 transcripts with consistent DNA similarity measurements along the gene body. In the upper panel, the transcripts were grouped based on DNA similarity and within each group we compared proportion of transcripts within different p-value ranges. In the lower panel, the transcripts were grouped based on strain x hindbrain interaction effect p-values and within each group we compared the proportion of transcripts with different DNA similarities.

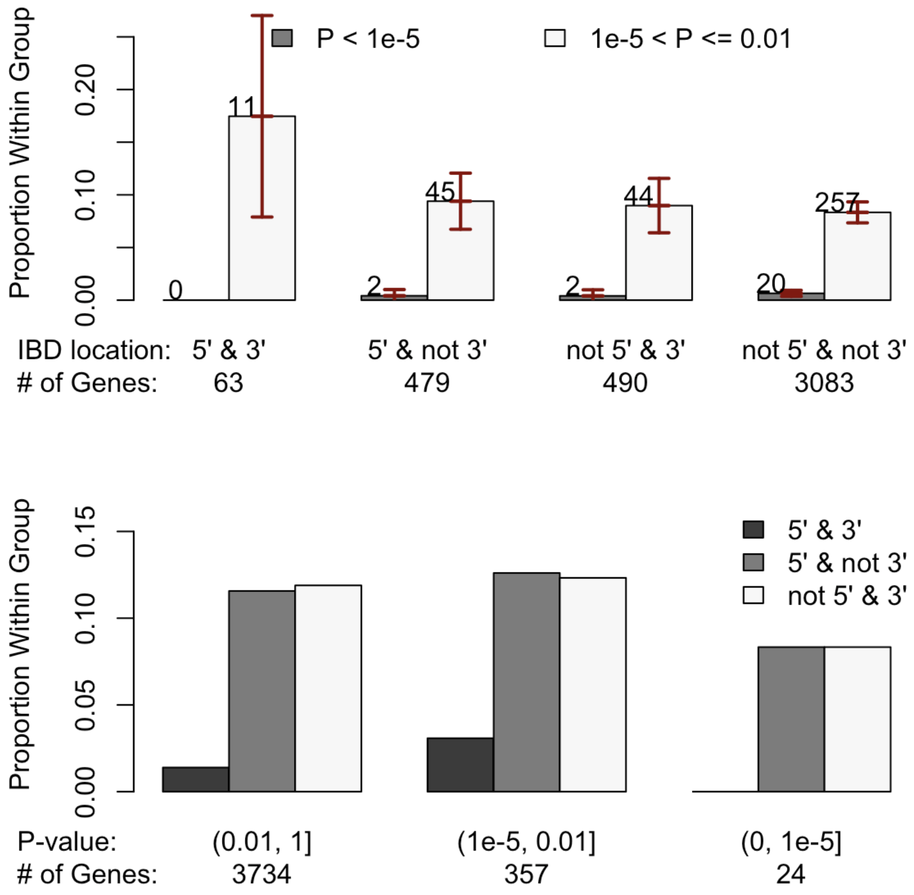

Figure S17 Comparison strain x hindbrain interaction effects vs. DNA similarity at 5' and 3' regions. Comparisons of the categories of transcripts based on p-value of strain x hindbrain interaction and IBD status at 5' and 3' regions, for the 4,118 transcripts with variable DNA similarity measurements in the gene body. Here IBD is defined as DNA similarity > 0.999. In the upper panel, the transcripts were grouped based on IBD location and within each group we compared proportion of transcripts within different p-value ranges. The vertical bars indicated the 95% confidence intervals. In the lower panel, the transcripts were grouped based on strain x hindbrain interaction p-values and within each group we compared the proportion of transcripts with different IBD location statuses.

Tables

Table S1 FDR at different p-value cutoffs for 1.1ST array.

p-value	Forebrain vs. hindbrain			Whole brain vs. forebrain			NOD vs. B6			Clozapine vs. vehicle			Interaction: hindbrain-NOD		
	D	FD	FDR	D	FD	FDR	D	FD	FDR	D	FD	FDR	D	FD	FDR
0.05	11141	680.5	0.061	4128	1093.9	0.265	6666	943.4	0.142	2418	1259	0.521	4071	1104.7	0.271
0.04	**10570**	**544.4**	**0.052**	3654	875.1	0.239	6099	754.7	0.124	2010	1007.2	0.501	3564	883.8	0.248
0.03	9910	408.3	0.041	3120	656.3	0.21	5495	566	0.103	1575	755.4	0.48	3032	662.8	0.219
0.02	9102	272.2	0.03	2508	437.6	0.174	4784	377.4	0.079	1125	503.6	0.448	2457	441.9	0.18
0.01	7954	136.1	0.017	1745	218.8	0.125	**3896**	**188.7**	**0.048**	638	251.8	0.395	1697	220.9	0.13
0.009	7810	122.5	0.016	1646	196.9	0.12	3759	169.8	0.045	588	226.6	0.385	1596	198.8	0.125
0.008	7648	108.9	0.014	1562	175	0.112	3628	150.9	0.042	530	201.4	0.38	1506	176.8	0.117
0.007	7455	95.3	0.013	1468	153.1	0.104	3496	132.1	0.038	472	176.3	0.374	1411	154.7	0.11
0.006	7219	81.7	0.011	1372	131.3	0.096	3338	113.2	0.034	412	151.1	0.367	1304	132.6	0.102
0.005	7005	68.0	0.01	1245	109.4	0.088	3178	94.3	0.03	346	125.9	0.364	1195	110.5	0.092
0.004	6748	54.4	0.008	1103	87.5	0.079	3029	75.5	0.025	274	100.7	0.368	1065	88.4	0.083
0.003	6403	40.8	0.006	993	65.6	0.066	2757	56.6	0.021	200	75.5	0.378	919	66.3	0.072
0.002	5976	27.2	0.005	**834**	**43.8**	**0.053**	2470	37.7	0.015	142	50.4	0.355	**778**	**44.2**	**0.057**
0.001	5330	13.6	0.003	625	21.9	0.035	2087	18.9	0.009	61	25.2	0.413	602	22.1	0.037
9e-04	5245	12.2	0.002	593	19.7	0.033	2040	17	0.008	53	22.7	0.428	579	19.9	0.034
8e-04	5135	10.9	0.002	563	17.5	0.031	1975	15.1	0.008	48	20.1	0.419	551	17.7	0.032
7e-04	5026	9.5	0.002	543	15.3	0.028	1911	13.2	0.007	46	17.6	0.383	526	15.5	0.029
6e-04	4902	8.2	0.002	510	13.1	0.026	1853	11.3	0.006	38	15.1	0.397	501	13.3	0.027
5e-04	4764	6.8	0.001	466	10.9	0.023	1766	9.4	0.005	33	12.6	0.382	453	11	0.024
4e-04	4598	5.4	0.001	427	8.8	0.021	1663	7.5	0.005	24	10.1	0.421	407	8.8	0.022
3e-04	4375	4.1	0.001	397	6.6	0.017	1545	5.7	0.004	17	7.6	0.447	364	6.6	0.018
2e-04	4112	2.7	0.001	339	4.4	0.013	1402	3.8	0.003	10	5	0.5	294	4.4	0.015
1e-04	3683	1.4	0	275	2.2	0.008	1206	1.9	0.002	4	2.5	0.625	237	2.2	0.009

For a p-value cutoff p, the expected number of false discoveries (FD) is $\pi 0Np$ (N is the total number of transcripts), the number of discoveries (D) was the number of transcripts with p-values < p, and FDR is calculated as FD/D.

Table S2 FDR at different p-value cutoffs for 1.0ST array.

p-value	Forebrain vs. hindbrain			Whole brain vs. forebrain			NOD vs. B6			Clozapine vs. vehicle			Interaction: hindbrain-NOD		
	D	FD	FDR	D	FD	FDR	D	FD	FDR	D	FD	FDR	D	FD	FDR
0.05	7351	888.2	0.121	2877	1235.3	0.429	7446	893.9	0.12	3391	1138.9	0.336	4196	1109.9	0.265
0.04	6837	710.6	0.104	2494	988.2	0.396	6877	715.1	0.104	2898	911.1	0.314	3733	887.9	0.238
0.03	6213	532.9	0.086	2088	741.2	0.355	6275	536.3	0.085	2355	683.3	0.29	3180	665.9	0.209
0.02	5429	355.3	0.065	1623	494.1	0.304	5502	357.6	0.065	1772	455.6	0.257	2585	444	0.172
0.01	**4401**	**177.6**	**0.04**	1125	247.1	0.22	**4465**	**178.8**	**0.04**	1085	227.8	0.21	1803	222	0.123
0.009	4266	159.9	0.037	1080	222.4	0.206	4328	160.9	0.037	1005	205	0.204	1710	199.8	0.117
0.008	4119	142.1	0.034	1010	197.6	0.196	4168	143	0.034	924	182.2	0.197	1619	177.6	0.11
0.007	3964	124.3	0.031	944	172.9	0.183	4015	125.1	0.031	834	159.4	0.191	1527	155.4	0.102
0.006	3776	106.6	0.028	881	148.2	0.168	3860	107.3	0.028	753	136.7	0.182	1406	133.2	0.095
0.005	3610	88.8	0.025	804	123.5	0.154	3696	89.4	0.024	663	113.9	0.172	1300	111	0.085
0.004	3417	71.1	0.021	726	98.8	0.136	3467	71.5	0.021	560	91.1	0.163	1179	88.8	0.075
0.003	3161	53.3	0.017	616	74.1	0.12	3187	53.6	0.017	459	68.3	0.149	1027	66.6	0.065
0.002	2823	35.5	0.013	498	49.4	0.099	2854	35.8	0.013	347	45.6	0.131	**865**	**44.4**	**0.051**
0.001	2351	17.8	0.008	364	24.7	0.068	2437	17.9	0.007	180	22.8	0.127	672	22.2	0.033
9e-04	2285	16	0.007	355	22.2	0.063	2365	16.1	0.007	167	20.5	0.123	652	20	0.031
8e-04	2212	14.2	0.006	337	19.8	0.059	2295	14.3	0.006	153	18.2	0.119	622	17.8	0.029
7e-04	2127	12.4	0.006	316	17.3	0.055	2233	12.5	0.006	135	15.9	0.118	588	15.5	0.026
6e-04	2034	10.7	0.005	**293**	**14.8**	**0.051**	2147	10.7	0.005	113	13.7	0.121	555	13.3	0.024
5e-04	1937	8.9	0.005	273	12.4	0.045	2051	8.9	0.004	92	11.4	0.124	519	11.1	0.021
4e-04	1811	7.1	0.004	259	9.9	0.038	1957	7.2	0.004	79	9.1	0.115	482	8.9	0.018
3e-04	1669	5.3	0.003	231	7.4	0.032	1828	5.4	0.003	61	6.8	0.111	438	6.7	0.015
2e-04	1521	3.6	0.002	203	4.9	0.024	1652	3.6	0.002	41	4.6	0.112	378	4.4	0.012
1e-04	1287	1.8	0.001	140	2.5	0.018	1386	1.8	0.001	23	2.3	0.1	310	2.2	0.007

For a p-value cutoff p, the expected number of false discoveries (FD) is $\pi 0Np$ (N is the total number of genes), the number of discoveries (D) was the number of genes with p-values < p, and FDR is FD/D.

Table S3 The 122 GO terms associated with brain region effects

GO ID	GO type	Size	GO Term Name	P-val F vs. H	P-val W vs. H
GO:0006887	BP	109	exocytosis	0	0
GO:0008283	BP	737	cell proliferation	0	0
GO:0016192	BP	446	vesicle-mediated transport	0	0
GO:0019226	BP	250	transmission of nerve impulse	0	0
GO:0005262	MF	70	calcium channel activity	0	0
GO:0007155	BP	614	cell adhesion	0	2e-04
GO:0022610	BP	614	biological adhesion	0	2e-04
GO:0048489	BP	28	synaptic vesicle transport	0	2e-04
GO:0000267	CC	607	cell fraction	0	2e-04
GO:0042391	BP	108	regulation of membrane potential	0	4e-04
GO:0051128	BP	281	regulation of cellular component organization	0	4e-04
GO:0007275	BP	2721	multicellular organismal development	0	6e-04
GO:0021987	BP	36	cerebral cortex development	0	6e-04
GO:0016788	MF	666	hydrolase activity, acting on ester bonds	0	6e-04
GO:0042578	MF	324	phosphoric ester hydrolase activity	0	6e-04
GO:0007267	BP	336	cell-cell signaling	0	8e-04
GO:0016790	MF	102	thiolester hydrolase activity	0	8e-04
GO:0031344	BP	54	regulation of cell projection organization	0	0.001
GO:0048869	BP	1765	cellular developmental process	0	0.001
GO:0008066	MF	29	glutamate receptor activity	0	0.001
GO:0016044	BP	276	cellular membrane organization	0	0.0014
GO:0042063	BP	50	gliogenesis	0	0.0014
GO:0007156	BP	78	homophilic cell adhesion	0	0.0016
GO:0022603	BP	200	regulation of anatomical structure morphogenesis	0	0.0018
GO:0045211	CC	126	postsynaptic membrane	0	0.002
GO:0006575	BP	159	cellular amino acid derivative metabolic process	0	0.0022
GO:0022604	BP	98	regulation of cell morphogenesis	0	0.0024
GO:0006897	BP	189	endocytosis	0	0.0028
GO:0010324	BP	189	membrane invagination	0	0.0028
GO:0050770	BP	39	regulation of axonogenesis	0	0.0032
GO:0051239	BP	767	regulation of multicellular organismal process	0	0.0032
GO:0008081	MF	69	phosphoric diester hydrolase activity	0	0.0032
GO:0006576	BP	102	cellular biogenic amine metabolic process	0	0.0042
GO:0007270	BP	37	nerve-nerve synaptic transmission	0	0.0048
GO:0048856	BP	2274	anatomical structure development	0	0.0048

GO:0048468	BP	801	cell development	0	0.0056
GO:0048168	BP	21	regulation of neuronal synaptic plasticity	0	0.0074
GO:0051493	BP	89	regulation of cytoskeleton organization	0	0.0076
GO:0030041	BP	53	actin filament polymerization	0	0.0086
GO:0016337	BP	178	cell-cell adhesion	0	0.009
GO:0004620	MF	61	phospholipase activity	0	0.0096
GO:0005267	MF	132	potassium channel activity	0	0.0126
GO:0000902	BP	360	cell morphogenesis	0	0.0134
GO:0008092	MF	395	cytoskeletal protein binding	0	0.0146
GO:0048646	BP	355	anatomical structure formation involved in morphogenesis	0	0.0148
GO:0032989	BP	398	cellular component morphogenesis	0	0.015
GO:0065003	BP	330	macromolecular complex assembly	0	0.0162
GO:0005886	CC	2802	plasma membrane	0	0.0182
GO:0044459	CC	1345	plasma membrane part	0	0.0186
GO:0007015	BP	95	actin filament organization	0	0.0188
GO:0030036	BP	186	actin cytoskeleton organization	0	0.022
GO:0003779	MF	290	actin binding	0	0.026
GO:0034622	BP	275	cellular macromolecular complex assembly	0	0.027
GO:0021954	BP	33	central nervous system neuron development	0	0.0308
GO:0032956	BP	59	regulation of actin cytoskeleton organization	0	0.0316
GO:0030029	BP	199	actin filament-based process	0	0.0336
GO:0010926	BP	791		0	0.0346
GO:0016298	MF	82	lipase activity	0	0.035
GO:0060284	BP	145	regulation of cell development	0	0.0354
GO:0019199	MF	69	transmembrane receptor protein kinase activity	0	0.0404
GO:0030155	BP	86	regulation of cell adhesion	0	0.0438
GO:0022607	BP	486	cellular component assembly	0	0.0448
GO:0051960	BP	138	regulation of nervous system development	0	0.0468
GO:0001725	CC	21	stress fiber	0	0.0516
GO:0004714	MF	56	transmembrane receptor protein tyrosine kinase activity	0	0.052
GO:0001525	BP	147	angiogenesis	0	0.0554
GO:0050767	BP	124	regulation of neurogenesis	0	0.0592
GO:0017016	MF	61	Ras GTPase binding	0	0.0638
GO:0007519	BP	91	skeletal muscle tissue development	0	0.0642
GO:0060538	BP	92	skeletal muscle organ development	0	0.0684
GO:0045664	BP	99	regulation of neuron differentiation	0	0.0704
GO:0017048	MF	22	Rho GTPase binding	0	0.1924
GO:0044456	CC	197	synapse part	2e-04	0
GO:0004721	MF	168	phosphoprotein phosphatase activity	4e-04	0

GO:0007268	BP	209	synaptic transmission	6e-04	0
GO:0032879	BP	361	regulation of localization	6e-04	0
GO:0070838	BP	140	divalent metal ion transport	6e-04	0
GO:0030425	CC	63	dendrite	8e-04	0
GO:0016791	MF	256	phosphatase activity	8e-04	0
GO:0006816	BP	137	calcium ion transport	0.001	0
GO:0044057	BP	162	regulation of system process	0.001	0
GO:0006461	BP	196	protein complex assembly	0.0012	0
GO:0070271	BP	196	protein complex biogenesis	0.0012	0
GO:0001505	BP	72	regulation of neurotransmitter levels	0.0016	0
GO:0007269	BP	48	neurotransmitter secretion	0.0018	0
GO:0016358	BP	48	dendrite development	0.0022	0
GO:0051049	BP	212	regulation of transport	0.0032	0
GO:0030665	CC	25	clathrin coated vesicle membrane	0.0032	0
GO:0050673	BP	79	epithelial cell proliferation	0.0034	0
GO:0046903	BP	264	secretion	0.004	0
GO:0032940	BP	235	secretion by cell	0.0042	0
GO:0044448	CC	65	cell cortex part	0.0042	0
GO:0051641	BP	732	cellular localization	0.0044	0
GO:0043005	CC	218	neuron projection	0.0044	0
GO:0016311	BP	148	dephosphorylation	0.0066	0
GO:0043112	BP	29	receptor metabolic process	0.0074	0
GO:0043687	BP	1117	post-translational protein modification	0.0106	0
GO:0030136	CC	75	clathrin-coated vesicle	0.0106	0
GO:0005097	MF	43	Rab GTPase activator activity	0.0108	0
GO:0050768	BP	39	negative regulation of neurogenesis	0.012	0
GO:0005516	MF	112	calmodulin binding	0.0124	0
GO:0003001	BP	119	generation of a signal involved in cell-cell signaling	0.0126	0
GO:0051046	BP	96	regulation of secretion	0.0128	0
GO:0005057	MF	93	receptor signaling protein activity	0.0136	0
GO:0008021	CC	59	synaptic vesicle	0.0152	0
GO:0046474	BP	45	glycerophospholipid biosynthetic process	0.0184	0
GO:0030662	CC	38	coated vesicle membrane	0.0184	0
GO:0030135	CC	90	coated vesicle	0.0194	0
GO:0060341	BP	117	regulation of cellular localization	0.0226	0
GO:0016529	CC	34	sarcoplasmic reticulum	0.027	0
GO:0009056	BP	1528	catabolic process	0.03	0
GO:0003713	MF	72	transcription coactivator activity	0.0312	0
GO:0016528	CC	36	sarcoplasm	0.0356	0
GO:0044248	BP	890	cellular catabolic process	0.0392	0

GO:0007416	BP	24	synapse assembly	0.0478	0
GO:0048873	BP	23	homeostasis of number of cells within a tissue	0.0716	0
GO:0017157	BP	24	regulation of exocytosis	0.1088	0
GO:0044444	CC	4121	cytoplasmic part	0.1206	0
GO:0016569	BP	68	covalent chromatin modification	0.1242	0
GO:0016570	BP	64	histone modification	0.1392	0
GO:0048872	BP	137	homeostasis of number of cells	0.1614	0
GO:0001894	BP	56	tissue homeostasis	0.1778	0

The columns “P-val F vs. H” and “P-val W vs. H” are the p-values comparing the expression of forebrain vs. hindbrain, and comparing whole brain vs. hindbrain, respectively.

Table S4 The 41 GO terms associated with strain effect in hindbrain.

GO ID	GO type	GO name	Size	P-value
GO:0000323	CC	lytic vacuole	160	0
GO:0005764	CC	lysosome	160	0
GO:0044456	CC	synapse part	197	0
GO:0008076	CC	voltage-gated potassium channel complex	57	0
GO:0034705	CC	potassium channel complex	57	0
GO:0030135	CC	coated vesicle	90	0
GO:0030136	CC	clathrin-coated vesicle	75	0
GO:0005789	CC	endoplasmic reticulum membrane	99	0
GO:0008021	CC	synaptic vesicle	59	0
GO:0005768	CC	endosome	242	8.00e-04
GO:0045202	CC	synapse	311	8.00e-04
GO:0044432	CC	endoplasmic reticulum part	118	8.00e-04
GO:0042175	CC	nuclear envelope-endoplasmic reticulum network	103	0.0012
GO:0044463	CC	cell projection part	103	0.0016
GO:0005773	CC	vacuole	187	0.0016
GO:0045211	CC	postsynaptic membrane	126	0.0016
GO:0031988	CC	membrane-bounded vesicle	251	0.002
GO:0016023	CC	cytoplasmic membrane-bounded vesicle	244	0.002
GO:0043025	CC	neuronal cell body	84	0.002
GO:0005856	CC	cytoskeleton	1008	0.0029
GO:0005097	MF	Rab GTPase activator activity	43	0
GO:0016301	MF	kinase activity	846	0
GO:0016627	MF	oxidoreductase activity, acting on the CH-CH group of donors	48	0
GO:0051536	MF	iron-sulfur cluster binding	48	0
GO:0051540	MF	metal cluster binding	48	0
GO:0005509	MF	calcium ion binding	829	0
GO:0046873	MF	metal ion transmembrane transporter activity	292	0
GO:0031406	MF	carboxylic acid binding	69	0
GO:0005215	MF	transporter activity	1048	0
GO:0016772	MF	transferase activity, transferring phosphorus-containing groups	981	4.00e-04
GO:0019842	MF	vitamin binding	119	4.00e-04
GO:0016597	MF	amino acid binding	25	4.00e-04
GO:0016773	MF	phosphotransferase activity, alcohol group as acceptor	698	8.00e-04
GO:0005267	MF	potassium channel activity	132	8.00e-04
GO:0005249	MF	voltage-gated potassium channel activity	85	8.00e-04
GO:0022843	MF	voltage-gated cation channel activity	132	8.00e-04
GO:0005261	MF	cation channel activity	252	8.00e-04

Table S5 The 35 pathways associated with brain region effect.

Pathway ID	Size	Pathway Name	P-val F vs. H	P-val W vs. H
05218	75	Melanoma	2e-04	0.0016
04720	77	Long-term potentiation	8e-04	2e-04
00562	55	Inositol phosphate metabolism	8e-04	0.0036
05216	31	Thyroid cancer	0.0018	0.0028
04730	76	Long-term depression	0.0026	0
04020	204	Calcium signaling pathway	0.003	0
04270	133	Vascular smooth muscle contraction	0.0036	0.0024
04810	224	Regulation of actin cytoskeleton	0	0.0064
04360	140	Axon guidance	0	0.0116
00500	36	Starch and sucrose metabolism	0	0.2154
04540	95	Gap junction	6e-04	0.0088
04520	87	Adherens junction	8e-04	0.0228
01040	28	Biosynthesis of unsaturated fatty acids	0.001	0.0368
04510	209	Focal adhesion	0.0014	0.0306
04320	22	Dorso-ventral axis formation	0.0016	0.0536
00512	29	O-Glycan biosynthesis	0.0026	0.2054
04010	287	MAPK signaling pathway	0.0028	0.0056
04920	71	Adipocytokine signaling pathway	0.003	0.116
04070	79	Phosphatidylinositol signaling system	0.0036	0.0042
04080	260	Neuroactive ligand-receptor interaction	0.004	0.0142
00380	41	Tryptophan metabolism	0.004	0.0672
05412	83	Arrhythmogenic right ventricular cardiomyopathy (ARVC)	0.0048	4e-04
04916	105	Melanogenesis	0.0076	4e-04
04912	105	GnRH signaling pathway	0.0084	0.0034
00564	70	Glycerophospholipid metabolism	0.0104	0.001
05214	70	Glioma	0.0166	0.0022
04144	221	Endocytosis	0.0188	0.0036
00563	25	Glycosylphosphatidylinositol(GPI)-anchor biosynthesis	0.0206	0.001
04512	83	ECM-receptor interaction	0.0222	0.002
05410	92	Hypertrophic cardiomyopathy (HCM)	0.0408	0.0012
00620	50	Pyruvate metabolism	0.044	0.0016
00310	45	Lysine degradation	0.072	0
04260	89	Cardiac muscle contraction	0.1026	0.0014
04514	159	Cell adhesion molecules (CAMs)	0.1278	0.0026
04662	90	B cell receptor signaling pathway	0.5024	0.0034

The columns “P-val F vs. H” and “P-val W vs. H” are the p-values comparing the expression of forebrain vs. hindbrain, and comparing whole brain vs. hindbrain, respectively.
