# Supplementary material for: Transcriptome Atlases of Mouse Brain Reveals Differential Expression Across Brain Regions and Genetic Backgrounds
Source: G3 (Bethesda). 2012 Feb 1;2(2):203–11. doi: 10.1534/g3.111.001602 (PMC3284328; doi:10.1534/g3.111.001602)
Supplement: Supporting Information [file supp_2.2.203_FigureS1.pdf]

Cerebellum Dissection

Important Note: The following dissections are performed using the aid of a dissecting microscope, and on filter paper moistened with cold RNAse-free 1X PBS, on top of a petri dish filled with ice. All surgical instruments are cleaned with RNAseZap in between each animal and brain region.

Method:
Cut one side at the cerebellar peduncle (fig.1, black line) with surgical scissors, or by pinching with forceps.

Note: Inferior colliculi have been removed for better visualization of the peduncles.
Removing the inferior colliculi is not necessary for the cerebellum dissection.

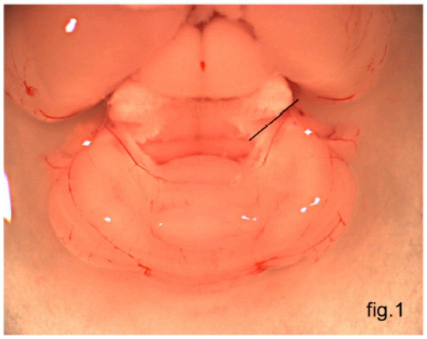

Gently push cerebellum to the side (fig.2).

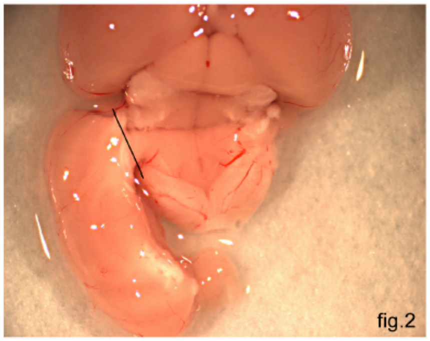

Snip the other cerebellar peduncle (fig.2, black line) to remove the cerebellum (cerebellum is shown removed in fig.3).
Immediately place tissue in the bottom of a 15ml Falcon tube, and immediately snap freeze on dry ice.

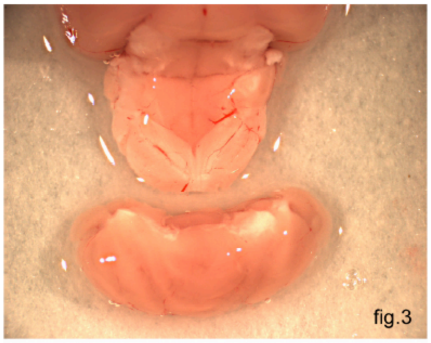

Figure S1 Removal of cerebellum.
