# Supplementary material for: Transcriptome Atlases of Mouse Brain Reveals Differential Expression Across Brain Regions and Genetic Backgrounds
Source: G3 (Bethesda). 2012 Feb 1;2(2):203–11. doi: 10.1534/g3.111.001602 (PMC3284328; doi:10.1534/g3.111.001602)
Supplement: Supporting Information [file supp_2.2.203_FigureS2.pdf]

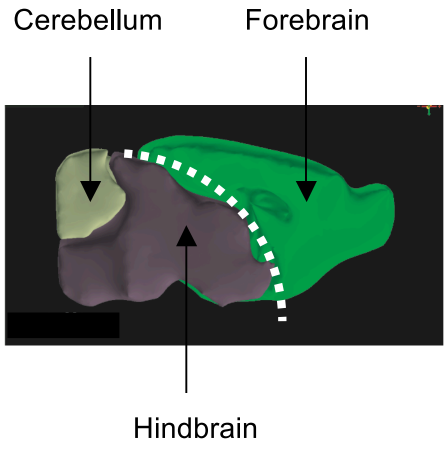

Figure S2 Separation of forebrain from hindbrain. One end of fine, curved forceps was wedged into the natural division between forebrain and hindbrain (dashed white line). The forceps were then closed, which acted to scoop out the hindbrain while leaving the underlying cortex intact.
