# Supplementary material for: Transcriptome Atlases of Mouse Brain Reveals Differential Expression Across Brain Regions and Genetic Backgrounds
Source: G3 (Bethesda). 2012 Feb 1;2(2):203–11. doi: 10.1534/g3.111.001602 (PMC3284328; doi:10.1534/g3.111.001602)
Supplement: Supporting Information [file supp_2.2.203_FigureS3.pdf]

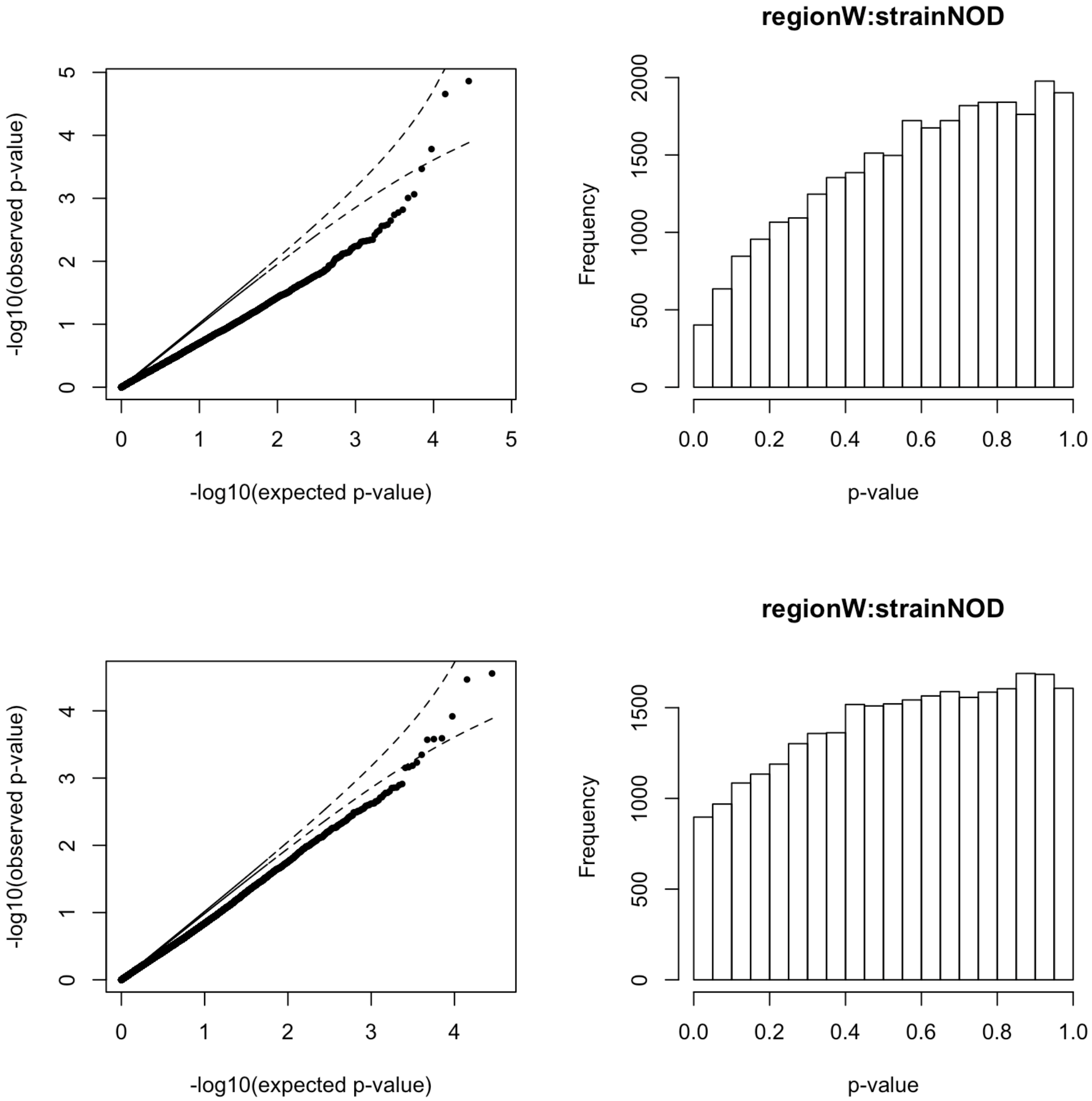

Figure S3 Conservative p-values from the initial analysis. In the initial analysis, we ran a linear mixed effect model for each gene. These figures illustrate a conservative p-value distribution for the interaction between whole brain region indicator and NOD strain indicator for 1.0ST cartridge array (upper panel) and 1.1ST GeneTitan array (lower panel). The left panel shows the QQ-plot of the p-values for the 35,556 genes. The broken lines indicate 95% confidence intervals if p-values follow a uniform distribution. The right panel is the histogram of the p-values. These figures clearly demonstrate conservative patterns, which imply the effects of unaccounted confounding variables.
