# Supplementary material for: Transcriptome Atlases of Mouse Brain Reveals Differential Expression Across Brain Regions and Genetic Backgrounds
Source: G3 (Bethesda). 2012 Feb 1;2(2):203–11. doi: 10.1534/g3.111.001602 (PMC3284328; doi:10.1534/g3.111.001602)
Supplement: Supporting Information [file supp_2.2.203_FigureS4.pdf]

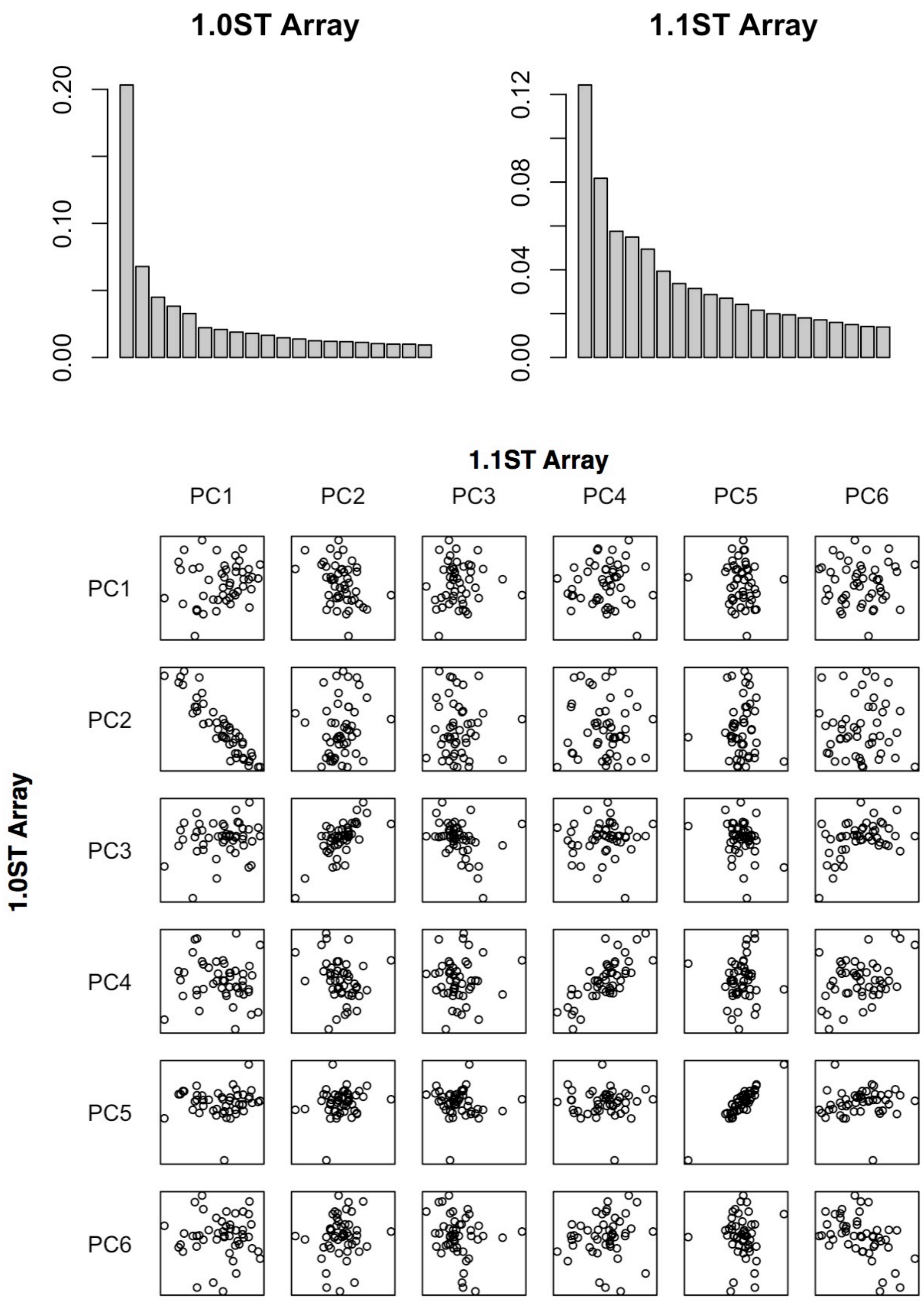

Figure S4 **Surrogate Variable Analysis. (a) The first 20 eigenvalues of the residuals from linear mixed effect model specified in equation (1) in main text Methods section for 1.0ST cartridge data. (b) The first 20 eigenvalues of the residuals of 1.1ST GeneTitan data. (c) Scatter plots of PC1-5 from expression data of the two platforms. GeneTitan PC1 is correlated with cartridge PC2, and PC3 and PC4 of the two platforms are correlated. We chose the first four PCs as surrogate variables since they either have large eigenvalues or are correlated with other PCs in the other platform.**
