# Supplementary material for: Transcriptome Atlases of Mouse Brain Reveals Differential Expression Across Brain Regions and Genetic Backgrounds
Source: G3 (Bethesda). 2012 Feb 1;2(2):203–11. doi: 10.1534/g3.111.001602 (PMC3284328; doi:10.1534/g3.111.001602)
Supplement: Supporting Information [file supp_2.2.203_FigureS5.pdf]

(a) Eigenvalues and projection plots for the first three PCs.

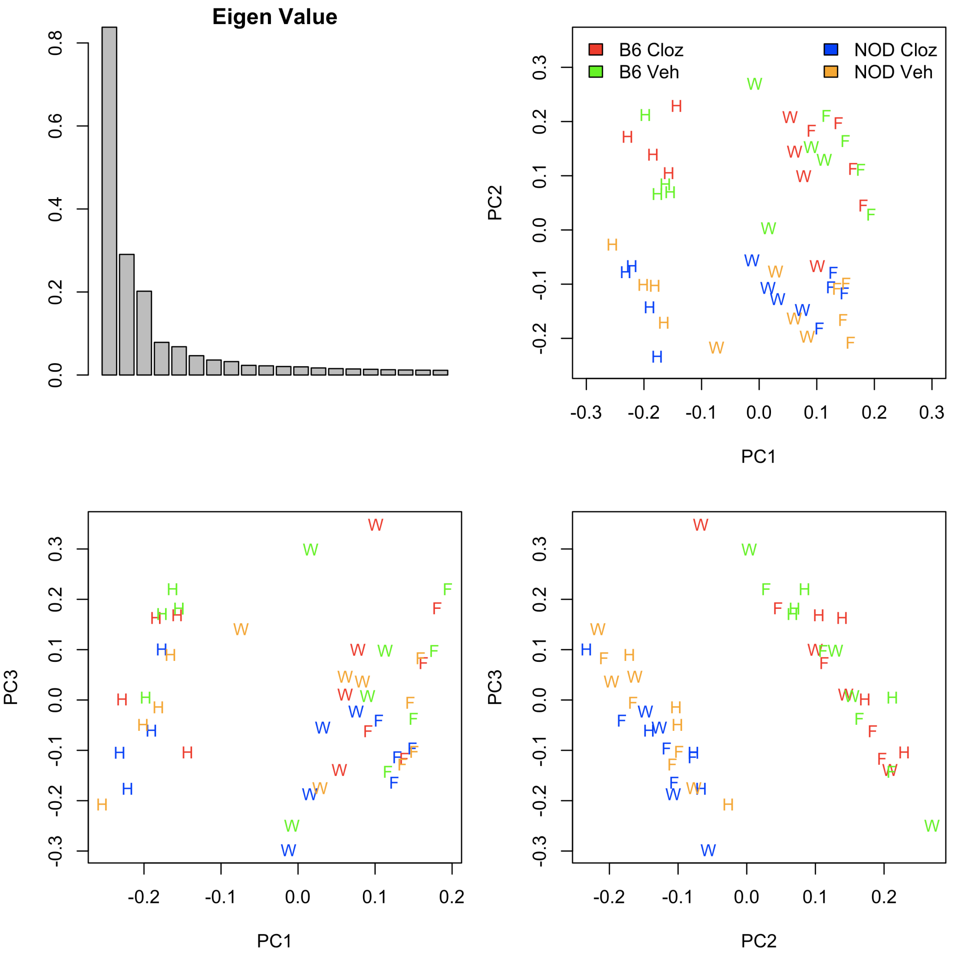

(B) R2 between the five covariates and the first 9 PCs. The covariate left/right indicate the sample is from left or right hemispheres.

	PC 1	PC 2	PC 3	PC 4	PC 5	PC 6	PC 7	PC 8	PC 9
left/right	0	0	0	0.01	0.05	0	0.02	0	0.02
drug	0	0	0.03	0.11	0	0.12	0.12	0.18	0.01
strain	0.01	0.79	0.19	0.01	0	0	0	0	0
region	0.94	0	0.02	0.04	0.07	0.01	0.39	0.02	0.01
day	0.04	0.06	0.02	0	0.42	0	0.02	0.01	0.03

Figure S5 PCA for gene expression from 1.0ST cartridge arrays.
