# Supplementary material for: Transcriptome Atlases of Mouse Brain Reveals Differential Expression Across Brain Regions and Genetic Backgrounds
Source: G3 (Bethesda). 2012 Feb 1;2(2):203–11. doi: 10.1534/g3.111.001602 (PMC3284328; doi:10.1534/g3.111.001602)
Supplement: Supporting Information [file supp_2.2.203_FigureS6.pdf]

(a) Eigenvalues and projection plots for the first three PCs.

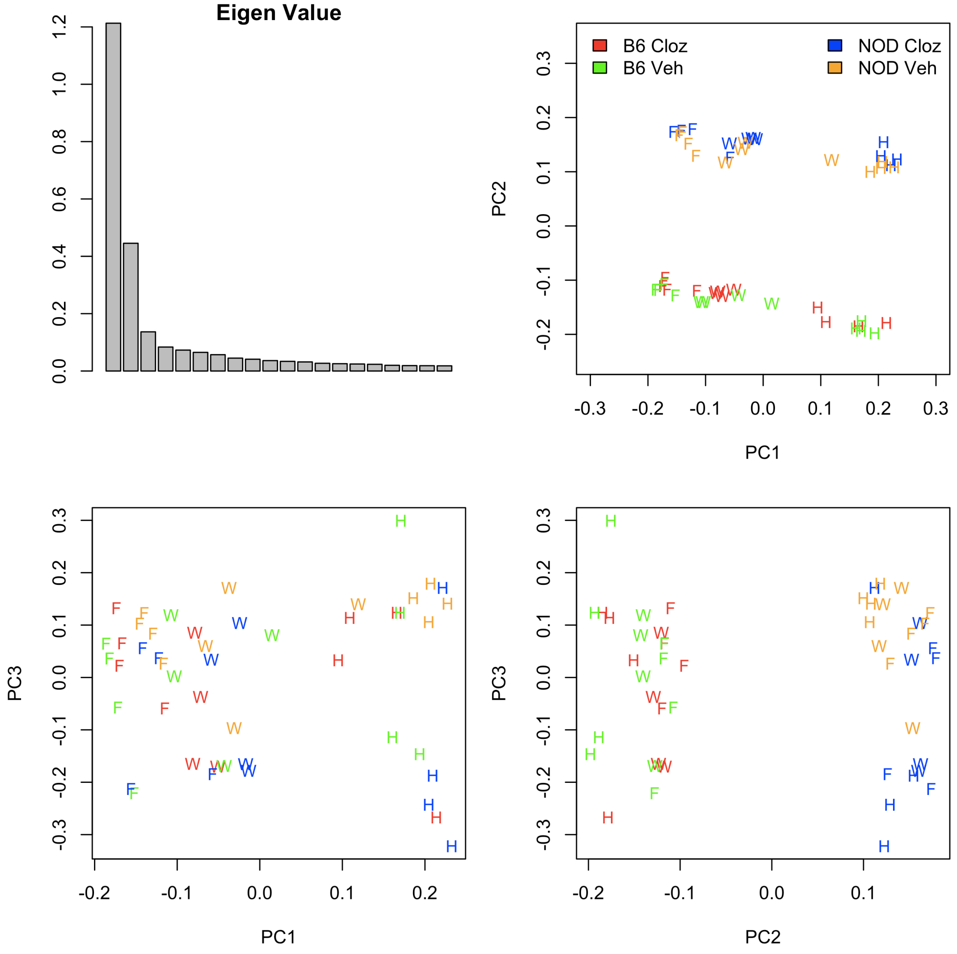

(b) R2 between the five covariates and the first 9 PCs.

	PC 1	PC 2	PC 3	PC 4	PC 5	PC 6	PC 7	PC 8	PC 9
left/right	0	0	0.01	0	0.01	0.03	0	0	0.01
drug	0	0	0.12	0.02	0.06	0.09	0.22	0.08	0.01
strain	0.03	0.96	0	0	0	0	0	0	0
region	0.92	0.02	0	0.13	0.07	0.08	0.1	0.1	0.08
day	0.03	0.04	0.01	0.13	0	0.08	0.02	0.05	0

Figure S6 PCA for gene expression from 1.1ST peg arrays.
