# Supplementary material for: Transcriptome Atlases of Mouse Brain Reveals Differential Expression Across Brain Regions and Genetic Backgrounds
Source: G3 (Bethesda). 2012 Feb 1;2(2):203–11. doi: 10.1534/g3.111.001602 (PMC3284328; doi:10.1534/g3.111.001602)
Supplement: Supporting Information [file supp_2.2.203_FigureS7.pdf]

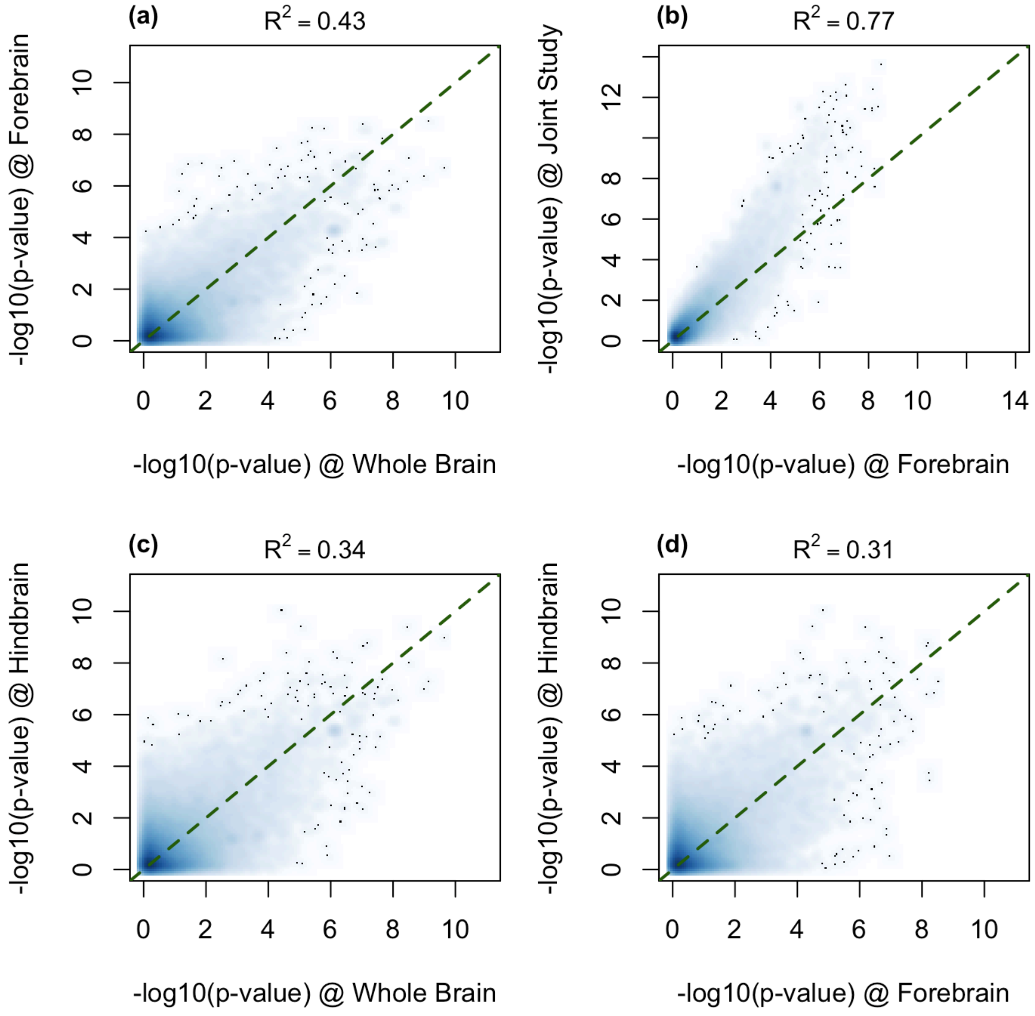

Figure S7 Comparison of brain-region-specific analysis and joint analysis. Comparison of strain effects assessed in different brain regions (by fixed effect model) or assessed by joint modeling three brain regions using linear mixed effect model. The R^2 is calculated as the correlation square of $-\log_{10}$(p-value).
