# Supplementary material for: Transcriptome Atlases of Mouse Brain Reveals Differential Expression Across Brain Regions and Genetic Backgrounds
Source: G3 (Bethesda). 2012 Feb 1;2(2):203–11. doi: 10.1534/g3.111.001602 (PMC3284328; doi:10.1534/g3.111.001602)
Supplement: Supporting Information [file supp_2.2.203_FigureS8.pdf]

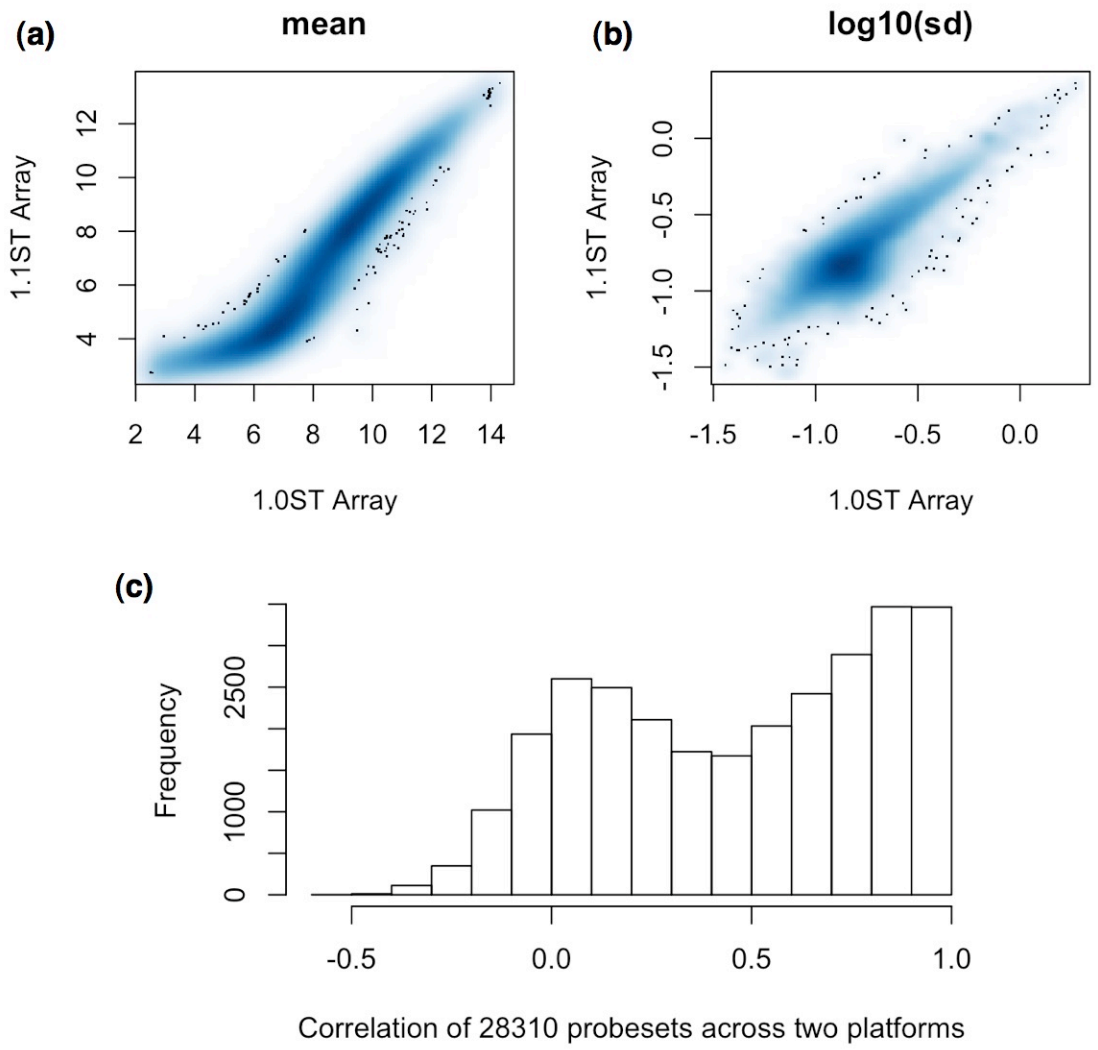

Figure S8 Comparisons of two platforms (I). (a) Scatter plot of the expression means of the 28,310 transcripts across the two platforms. (b) Scatter plot of the expression standard deviations of the 28,310 transcripts across the two platforms. (c) Histogram of the correlations of transcript expression across the two platforms. The correlation of each transcript is calculated as follows. Since there are 48 samples for each platform, the expression data of one transcript across the two platforms are two vectors of length 48, and the correlations of these two vectors were calculated.
