# Supplementary material for: Transcriptome Atlases of Mouse Brain Reveals Differential Expression Across Brain Regions and Genetic Backgrounds
Source: G3 (Bethesda). 2012 Feb 1;2(2):203–11. doi: 10.1534/g3.111.001602 (PMC3284328; doi:10.1534/g3.111.001602)
Supplement: Supporting Information [file supp_2.2.203_FigureS9.pdf]

(a) Cross-platform correlations and mean expression level and standard deviations.

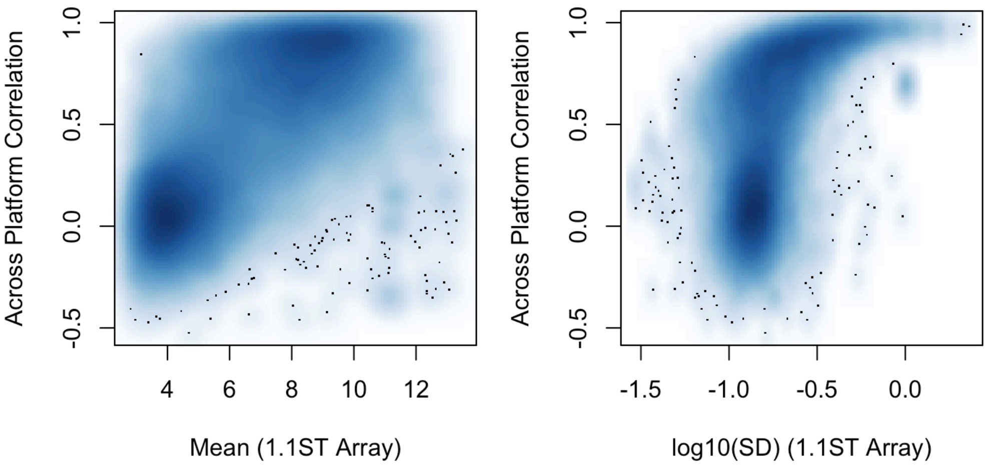

(b) The overlap of the differentially expressed genes across the two platforms.

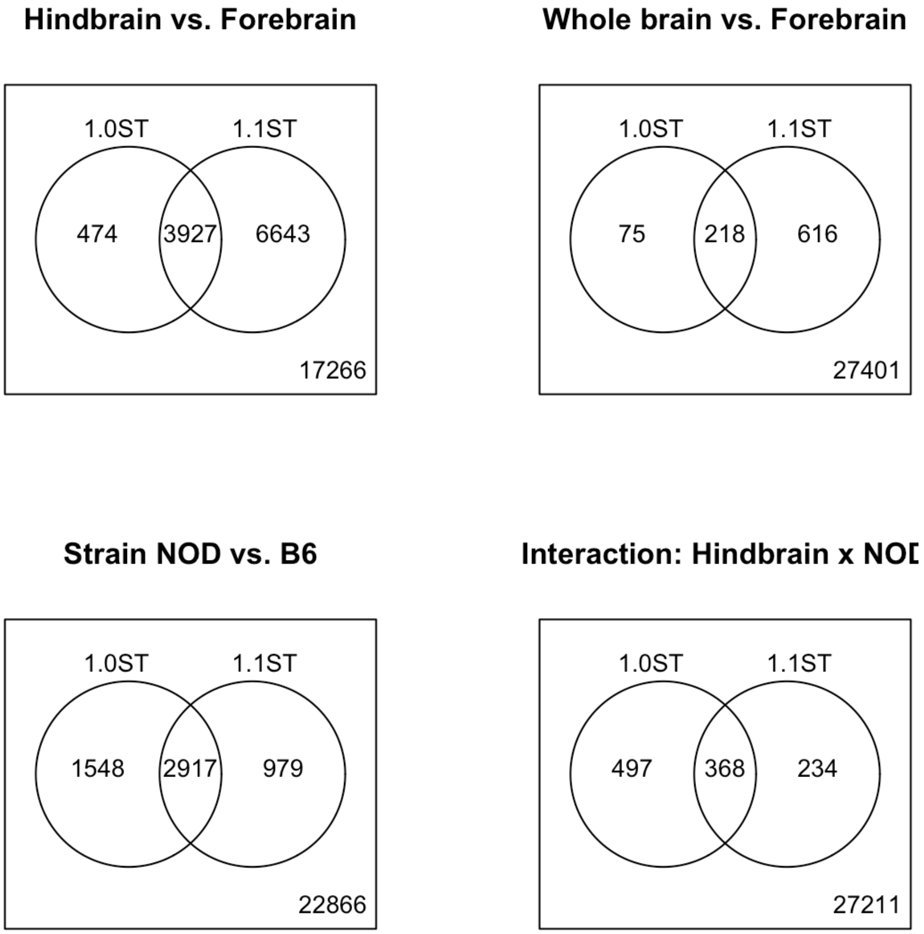

Figure S9 Comparisons of two platforms (II).
