# Supplementary material for: Transcriptome Atlases of Mouse Brain Reveals Differential Expression Across Brain Regions and Genetic Backgrounds
Source: G3 (Bethesda). 2012 Feb 1;2(2):203–11. doi: 10.1534/g3.111.001602 (PMC3284328; doi:10.1534/g3.111.001602)
Supplement: Supporting Information [file supp_2.2.203_FigureS10.pdf]

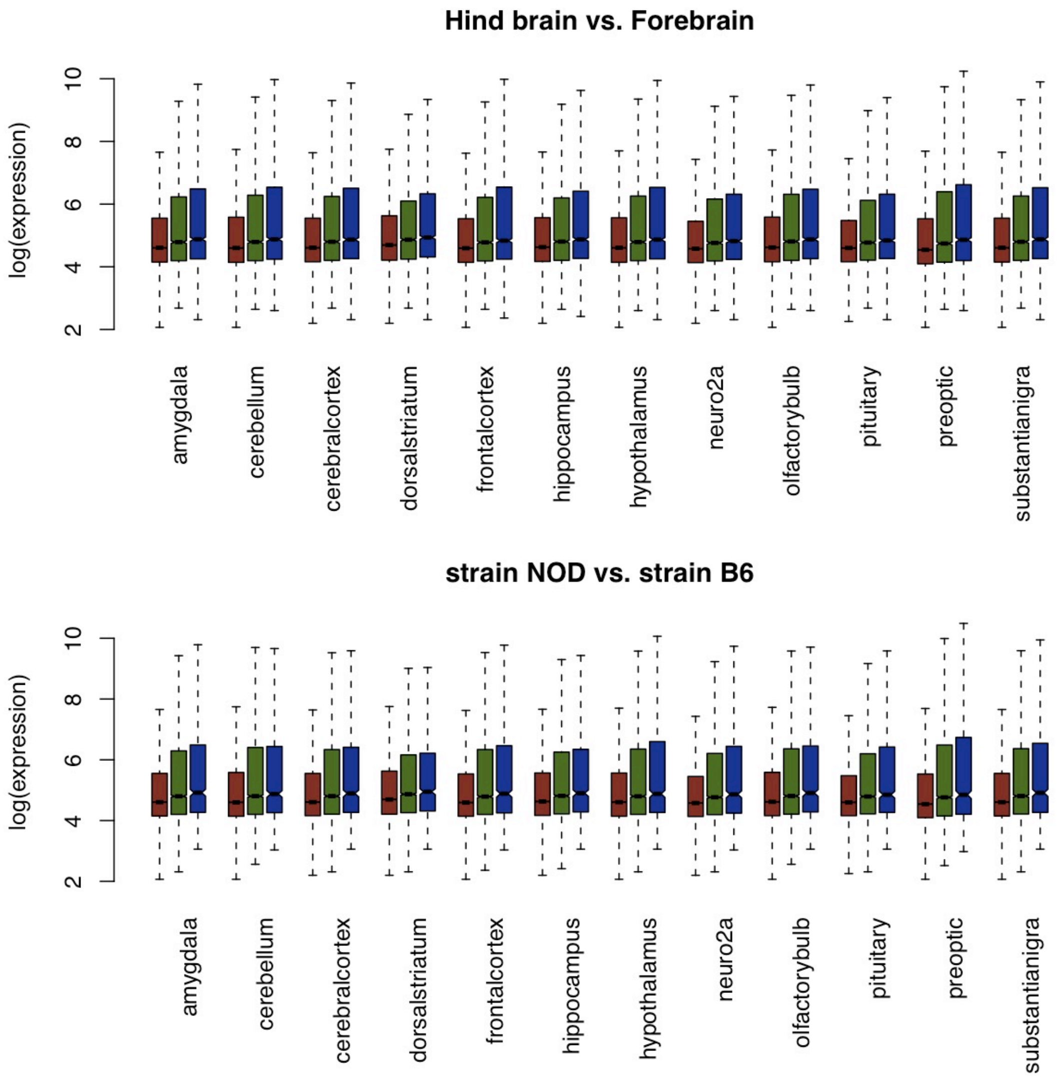

Figure S10 Comparison with GNF Atlas data. GNF atlas gene expression and array annotation (GNF1M) were downloaded from http://biogps.org/downloads/. The expressions of two replicates of each brain tissue were log transformed and then averaged. Probe sets of 1.1ST array and GNF 1M array were matched through NCBI refseq IDs. The red boxplots were the expression from all probe sets in the GNF 1M array. In the upper panel, the green and blue boxplots showed the expression of the genes in GNF1M arrays that were equivalently expressed (p-value > 0.1) or differentially expressed (p-value < 0.001) between forebrain and hindbrain in 1.1ST arrays. In the lower panel, the green and blue boxplots showed the expression of the genes in GNF1M arrays that were equivalently expressed (p-value > 0.1) or differentially expressed (p-value < 0.001) between two strains in the 1.1ST arrays. Note equivalently expressed genes have higher expression level in GNF 1M array partly because genes that can be mapped by refseq IDs have higher expression level.
