# Supplementary material for: Transcriptome Atlases of Mouse Brain Reveals Differential Expression Across Brain Regions and Genetic Backgrounds
Source: G3 (Bethesda). 2012 Feb 1;2(2):203–11. doi: 10.1534/g3.111.001602 (PMC3284328; doi:10.1534/g3.111.001602)
Supplement: Supporting Information [file supp_2.2.203_FigureS11.pdf]

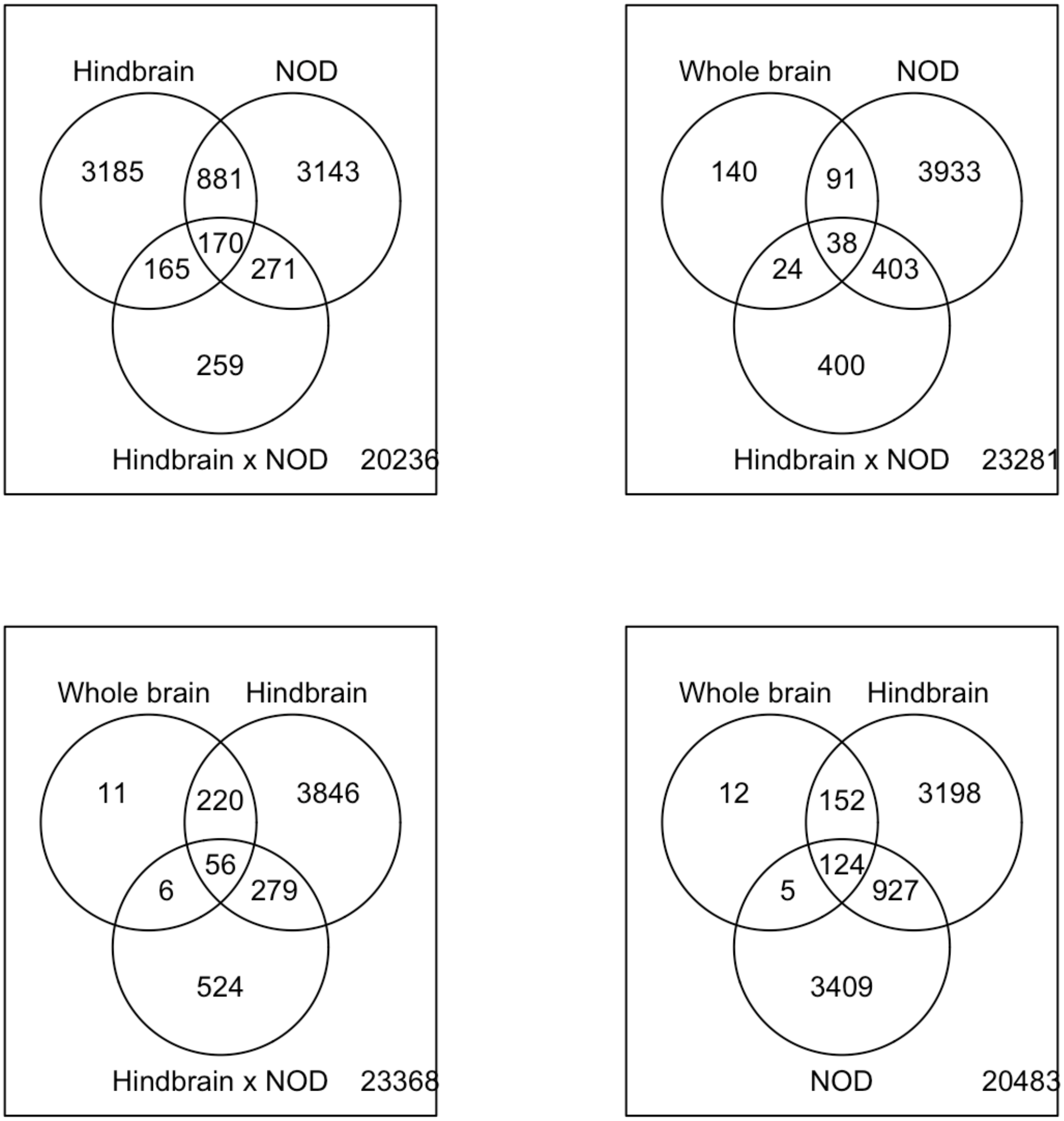

Figure S11 Comparison of differentially expressed genes in 1.0ST cartridge array. This Venn diagram is based on the results from linear mixed effect model for all the 48 samples and p-value cutoff for differential expression is decided by FDR < 0.05. Circles labeled by Hindbrain/Whole brain describe the differential expression between forebrain and Hindbrain/ Whole brain, circles labeled by NOD describe the differential expression between strains B6 and NOD, and Hindbrain x NOD indicates interaction term between Hindbrain indicator and NOD strain indicator.
