# Supplementary figures and images for: Transcriptome Atlases of Mouse Brain Reveals Differential Expression Across Brain Regions and Genetic Backgrounds
Source: G3 (Bethesda). 2012 Feb 1;2(2):203–11. doi: 10.1534/g3.111.001602 (PMC3284328; doi:10.1534/g3.111.001602)

1

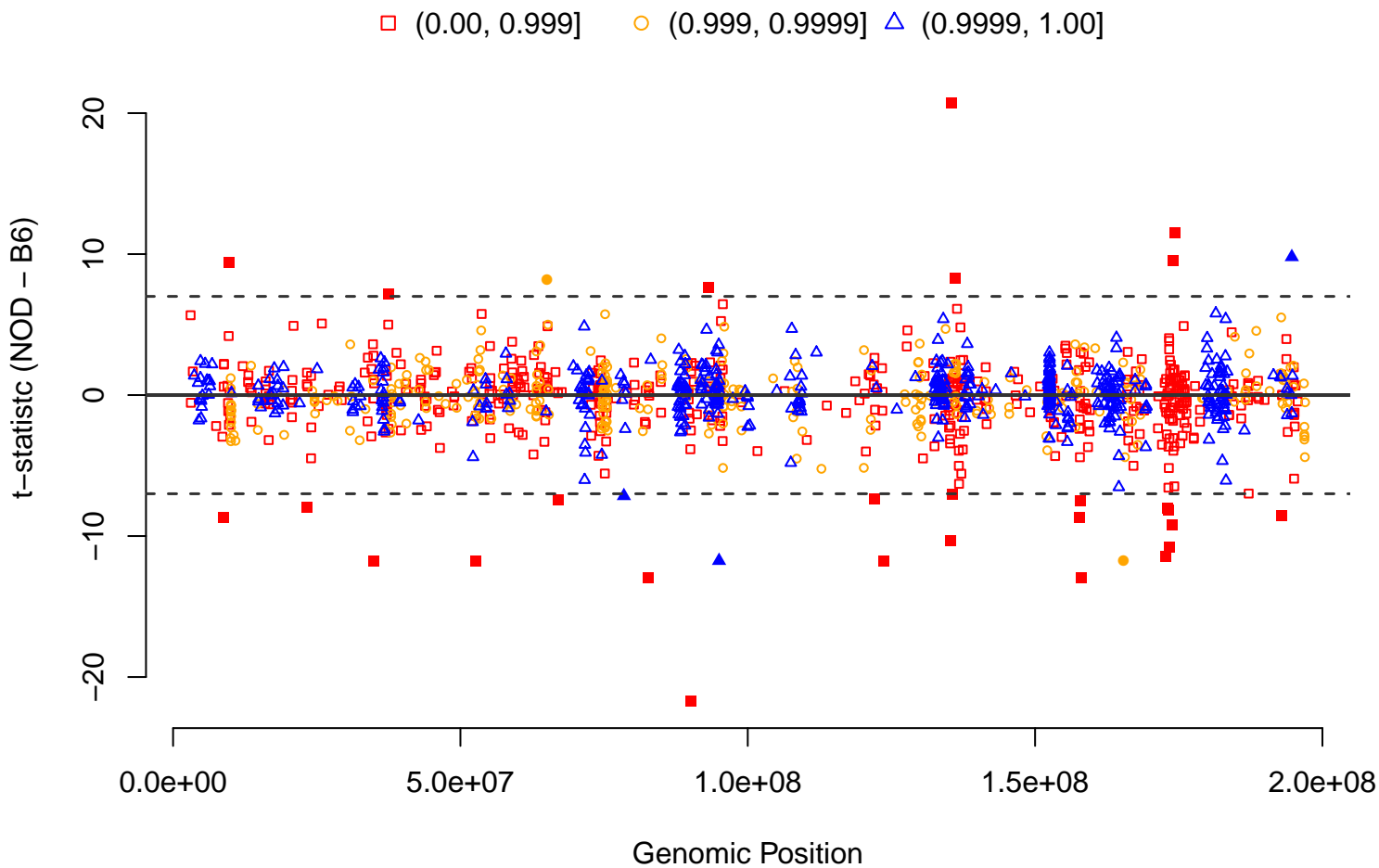

2

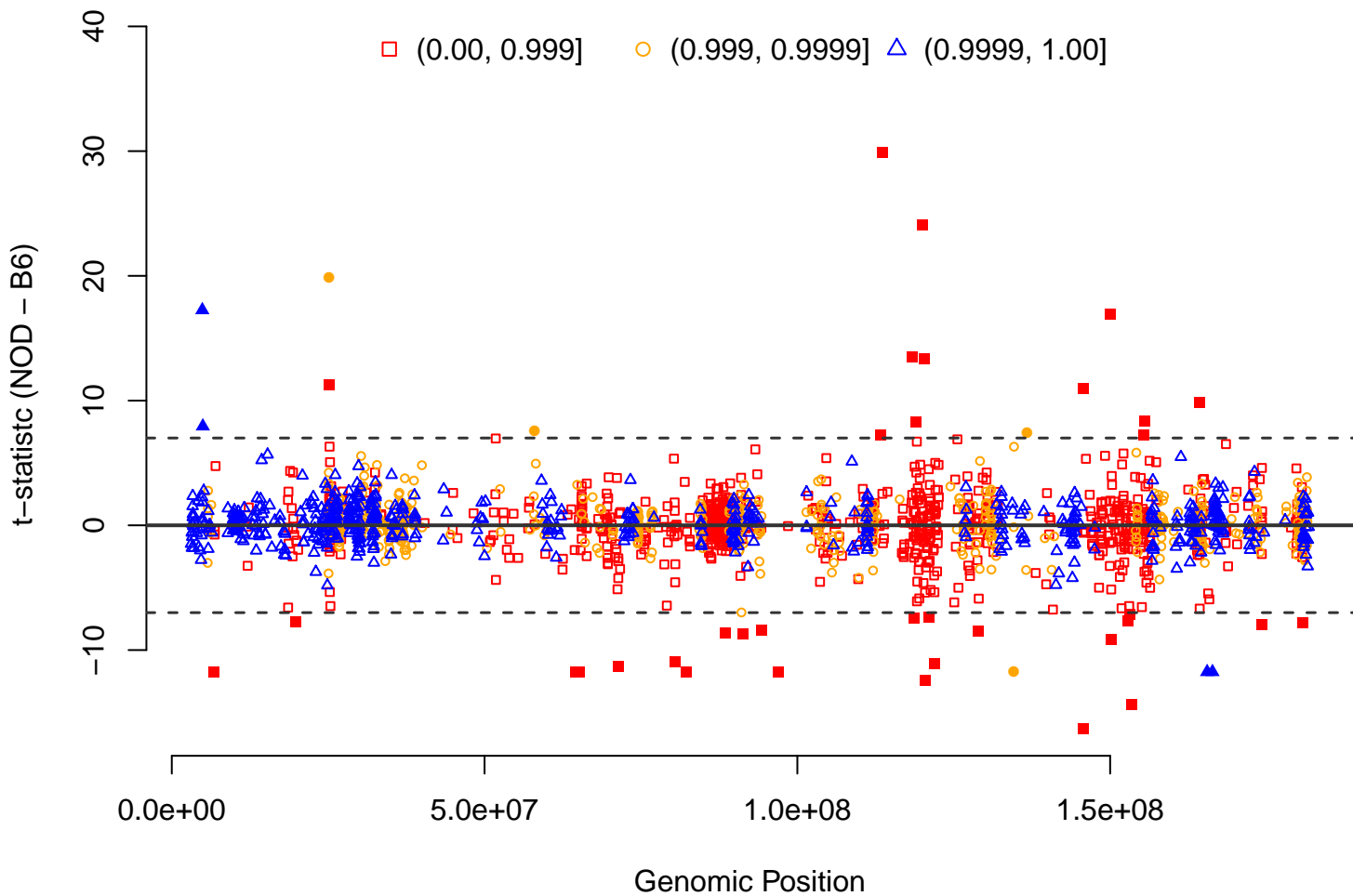

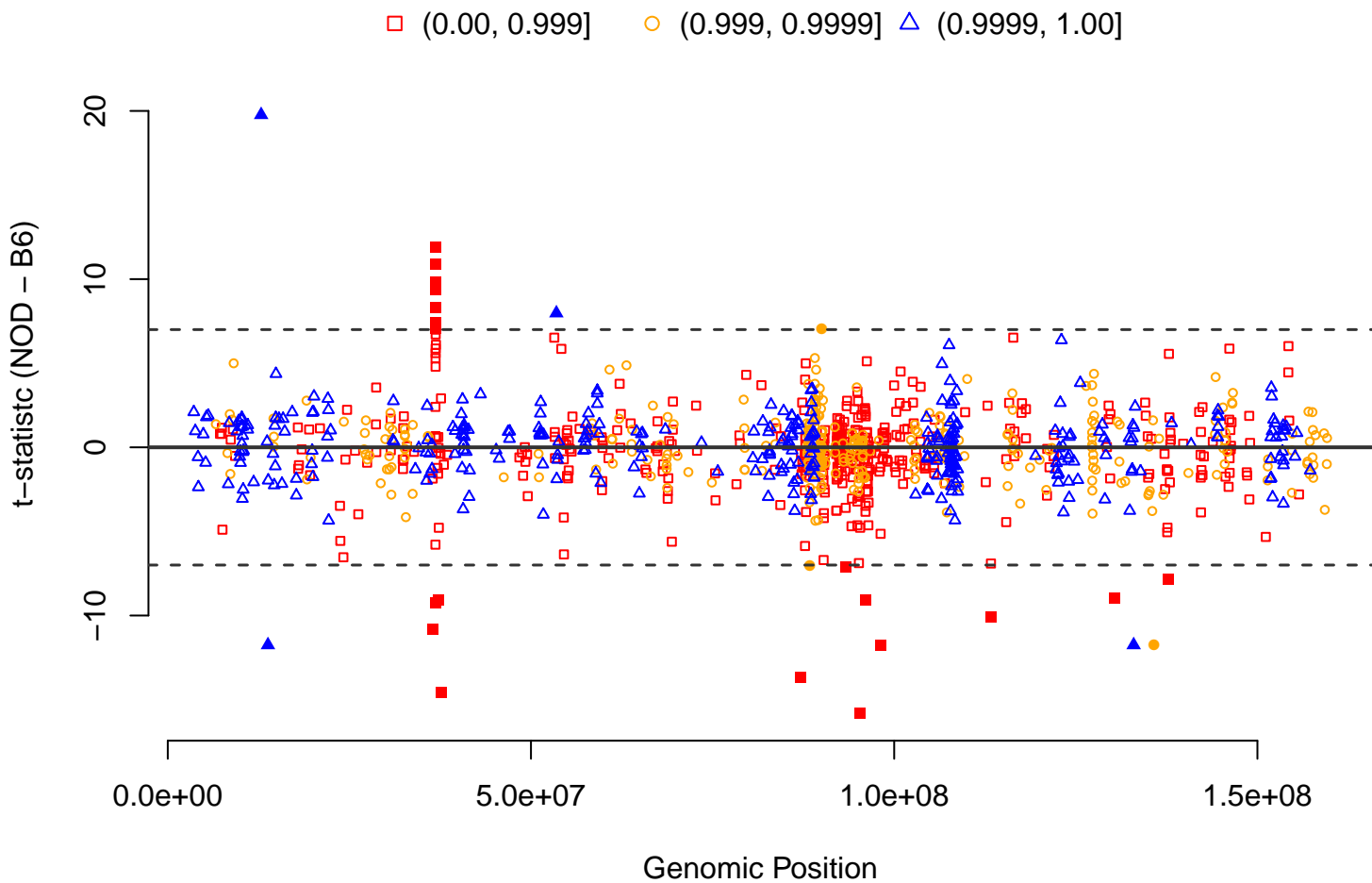

4

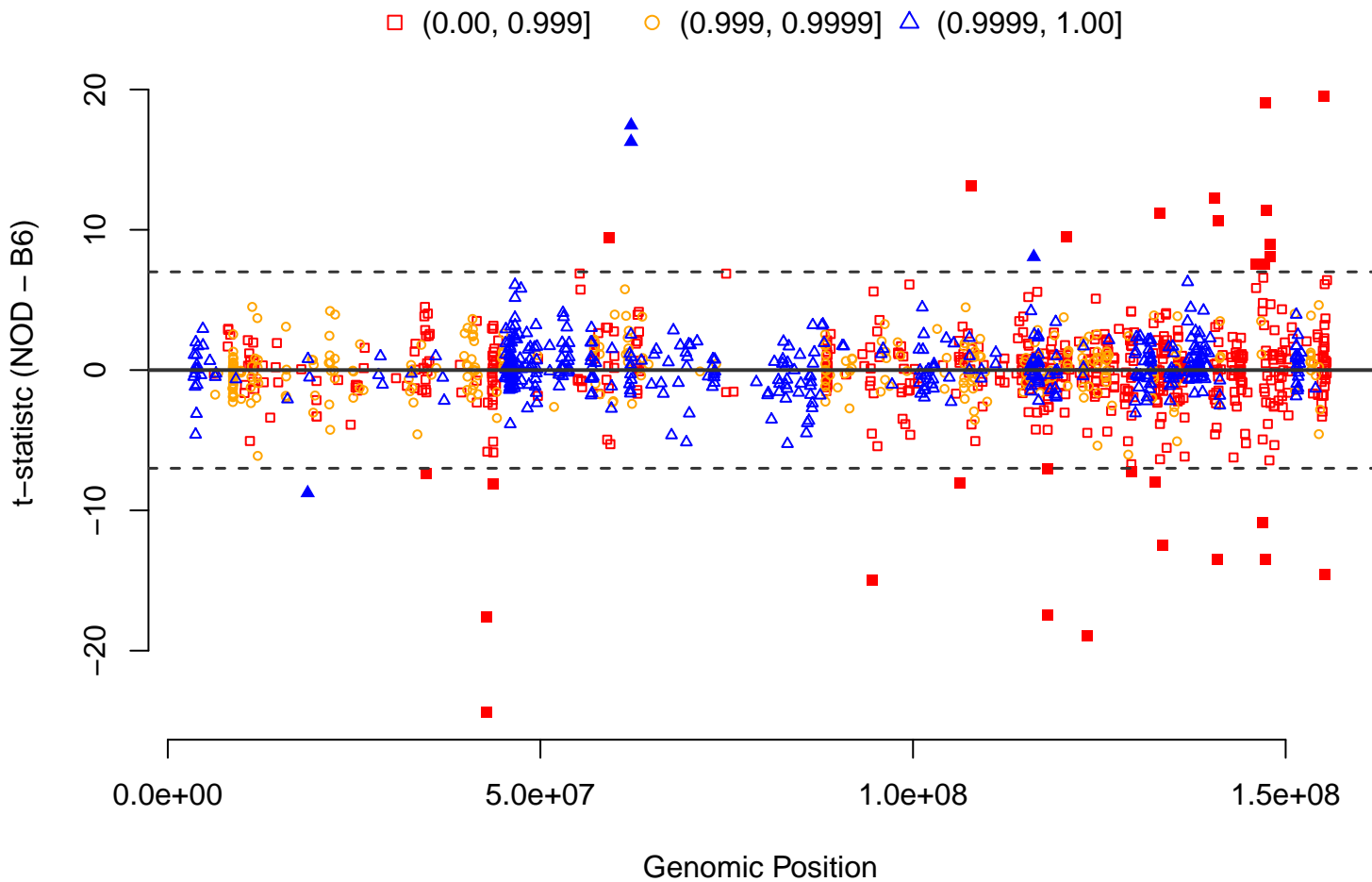

5

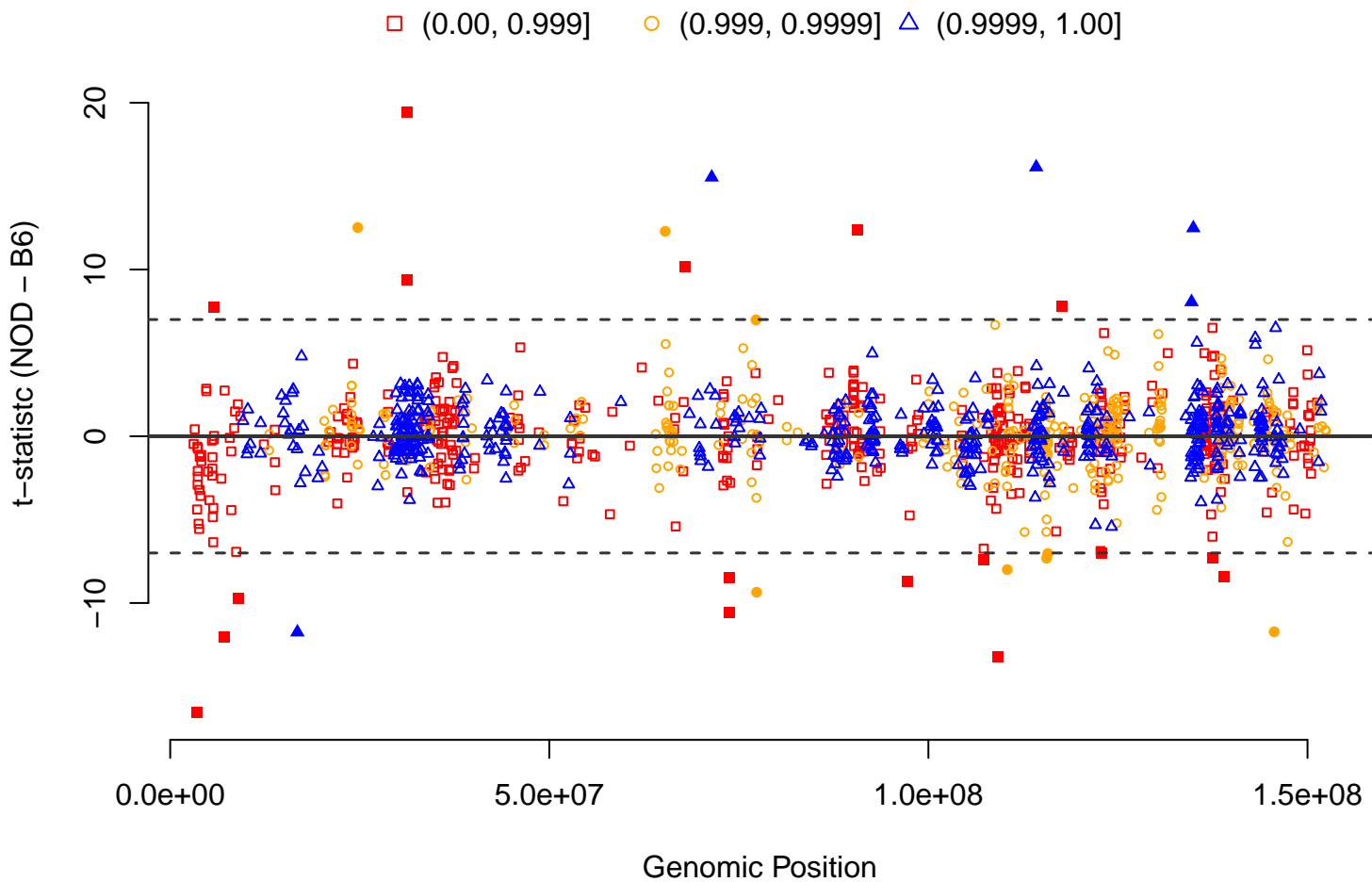

6

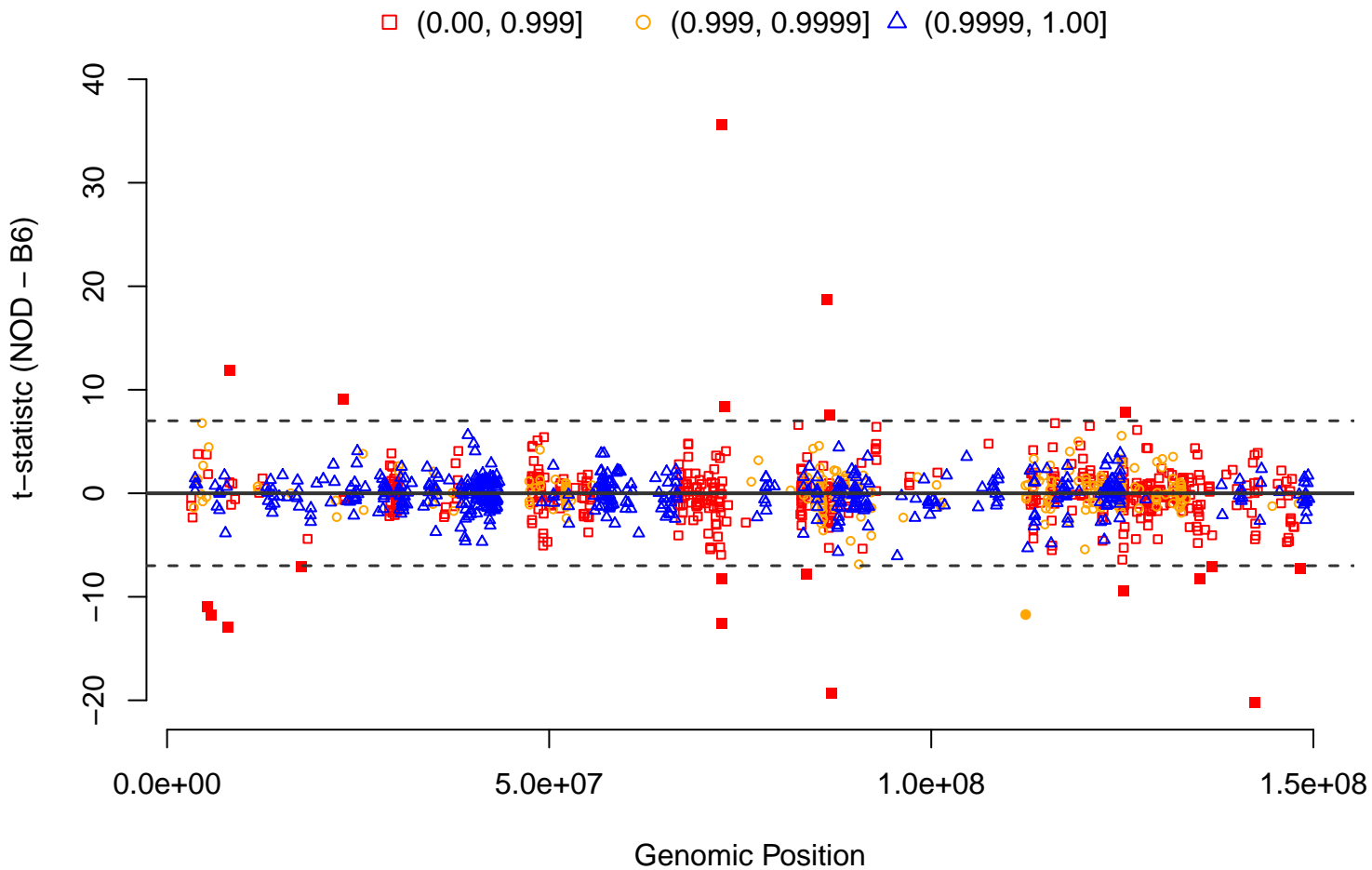

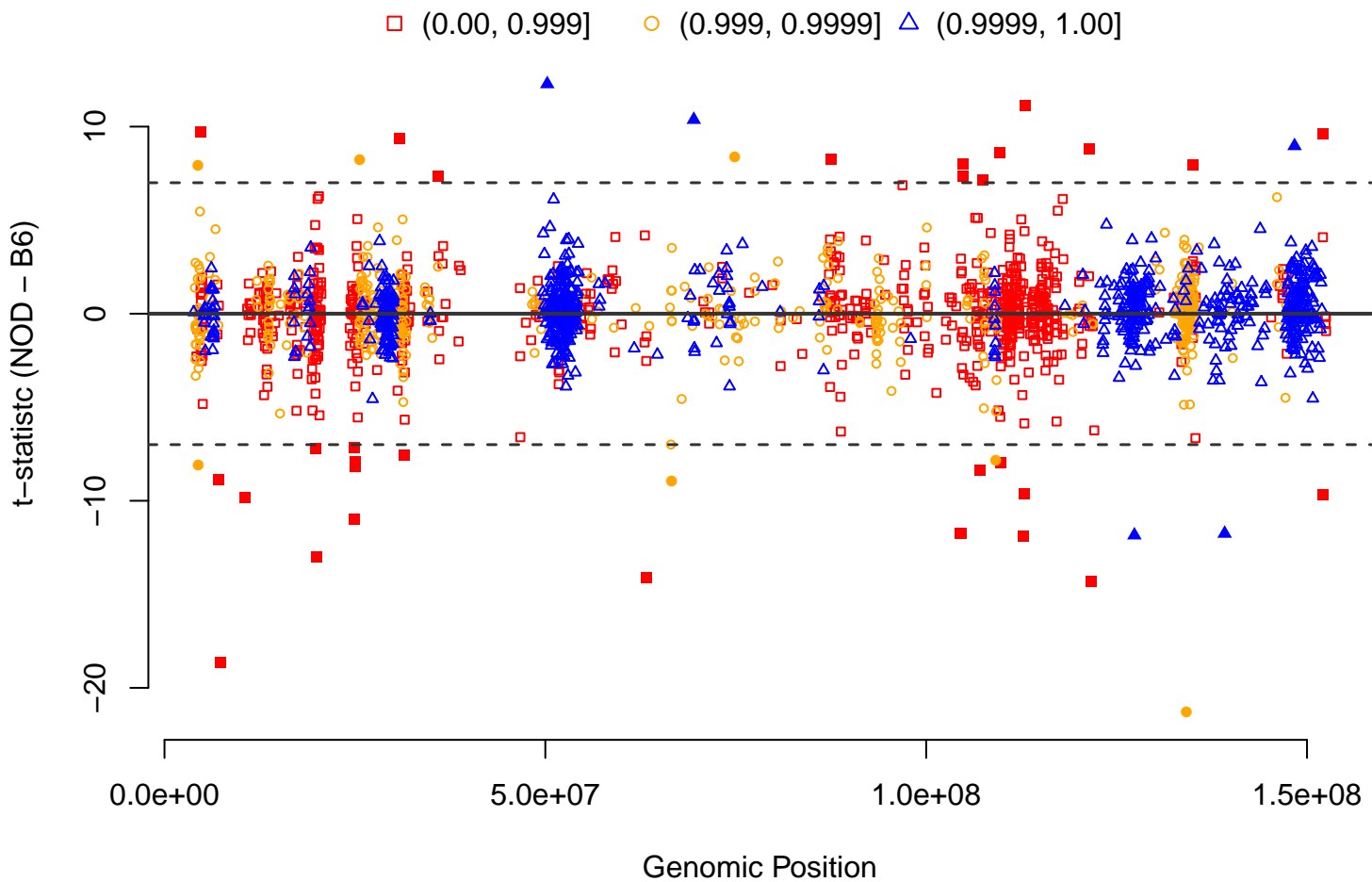

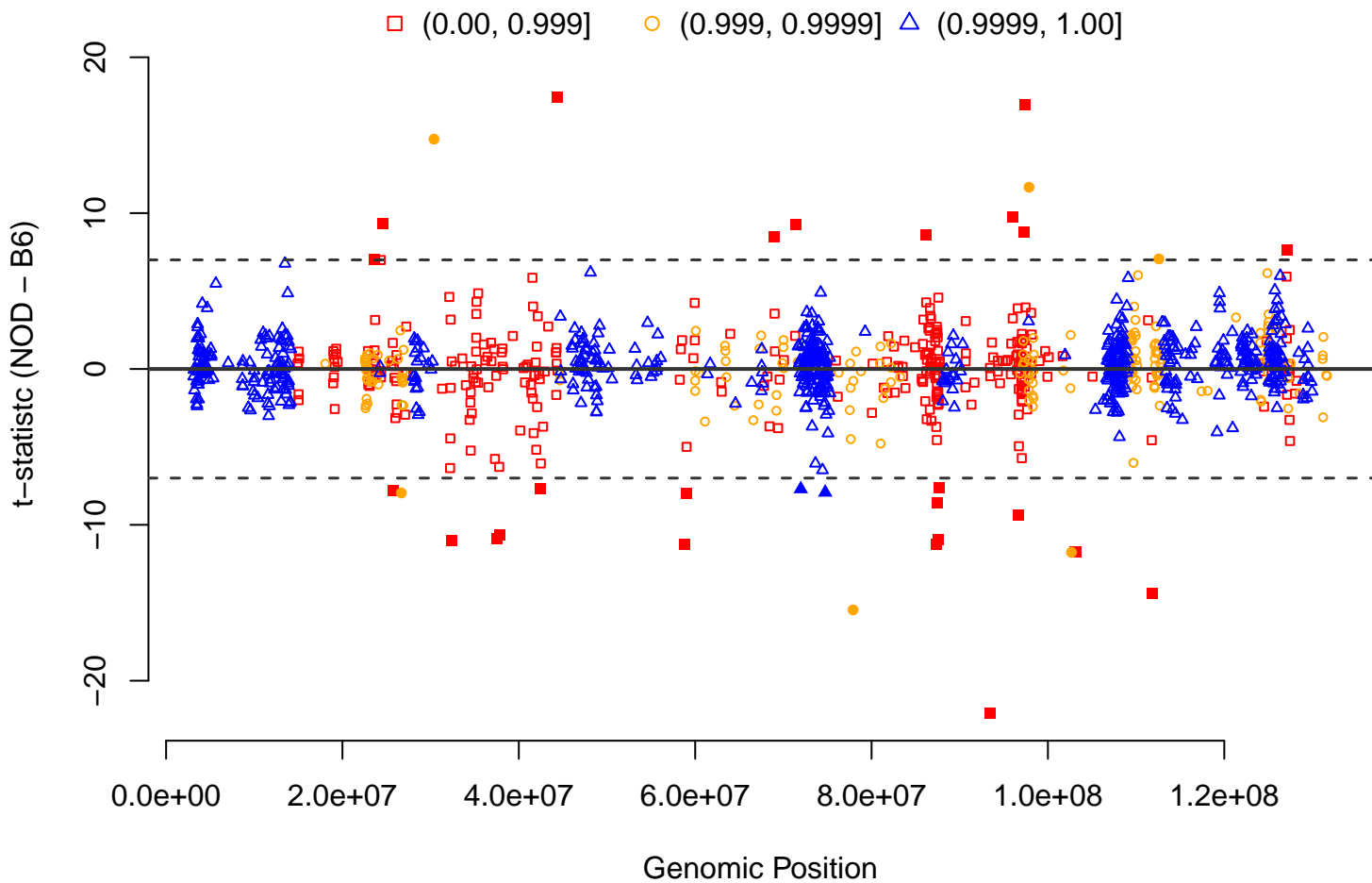

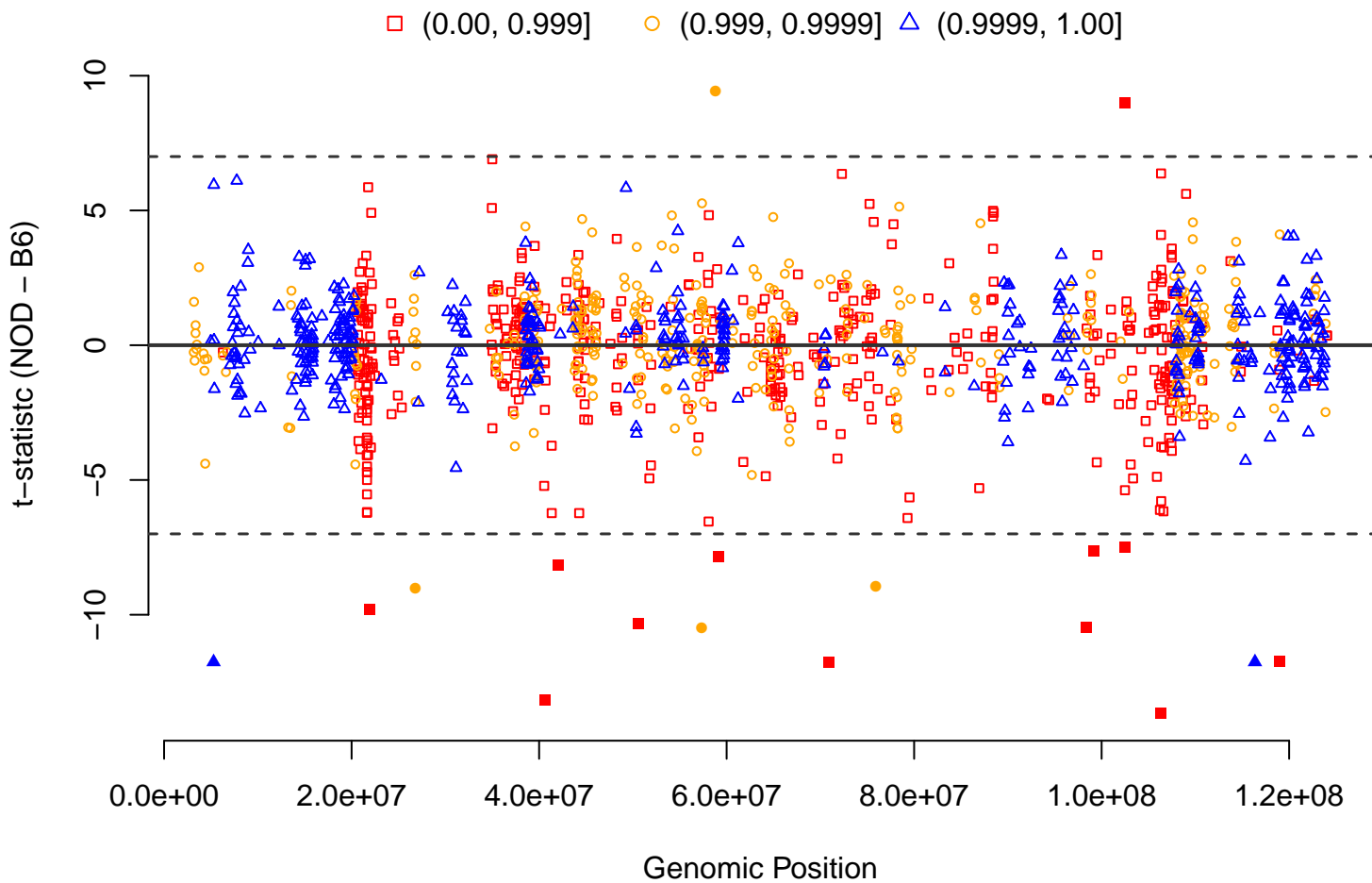

10

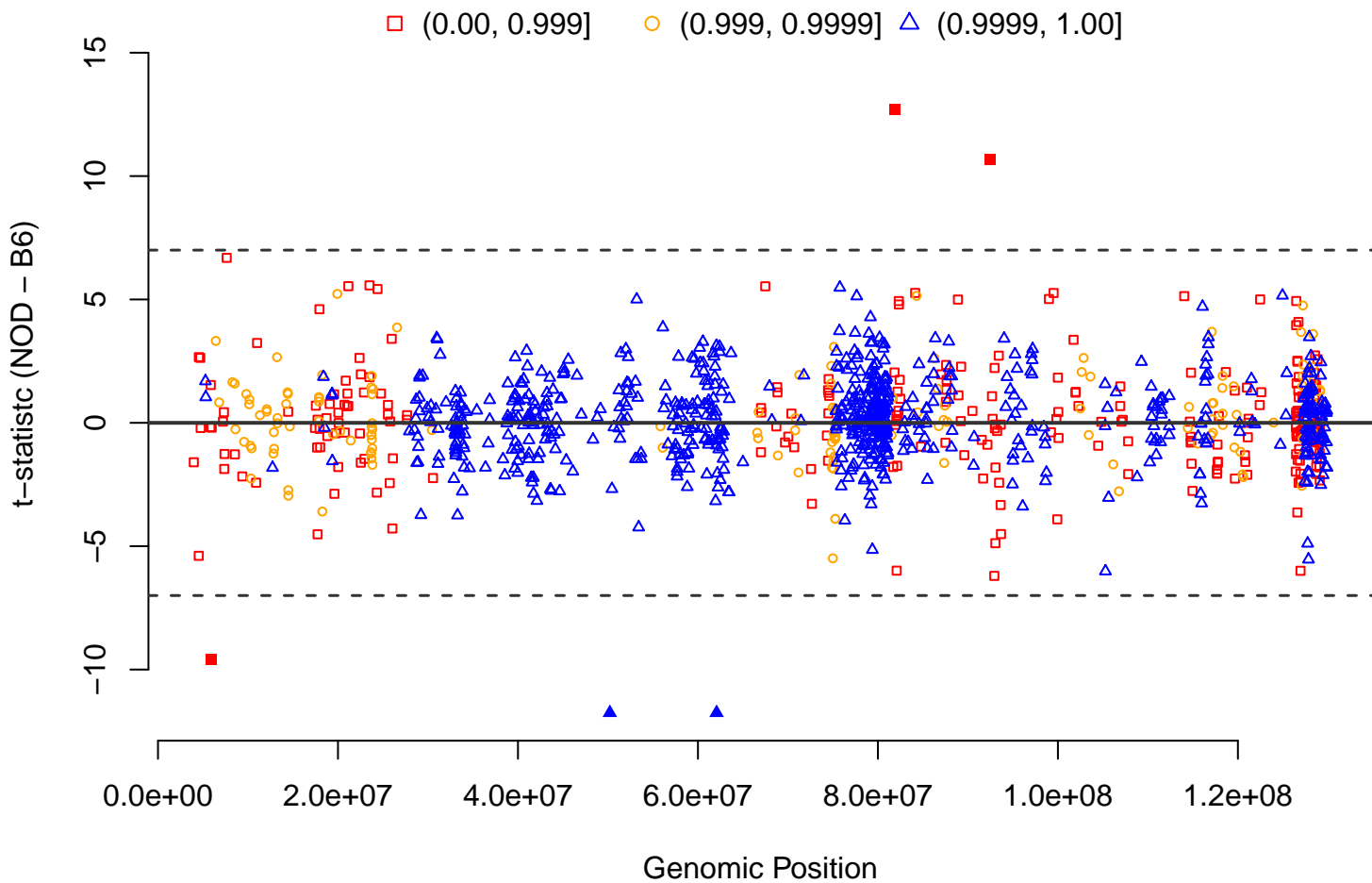

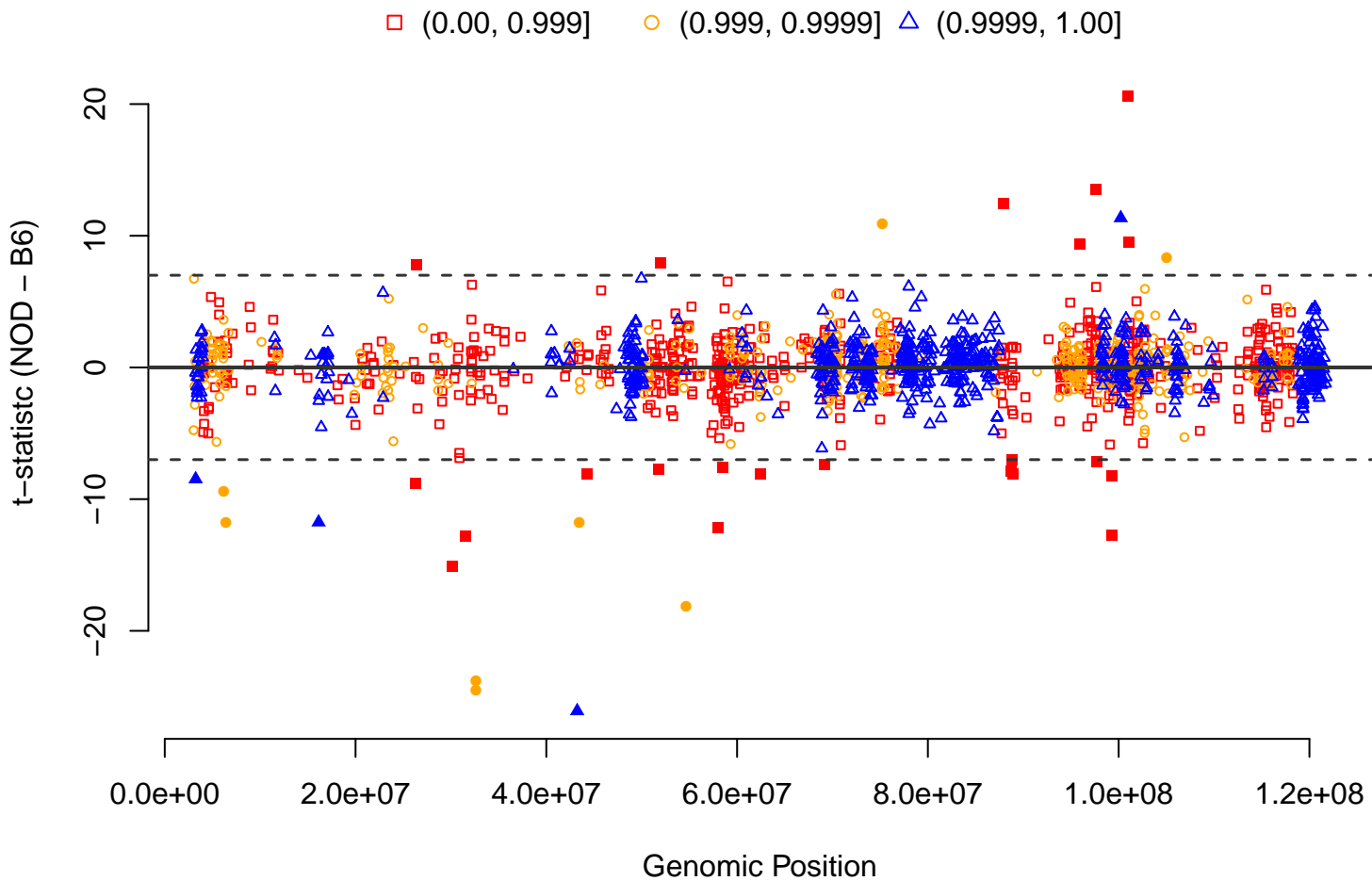

12

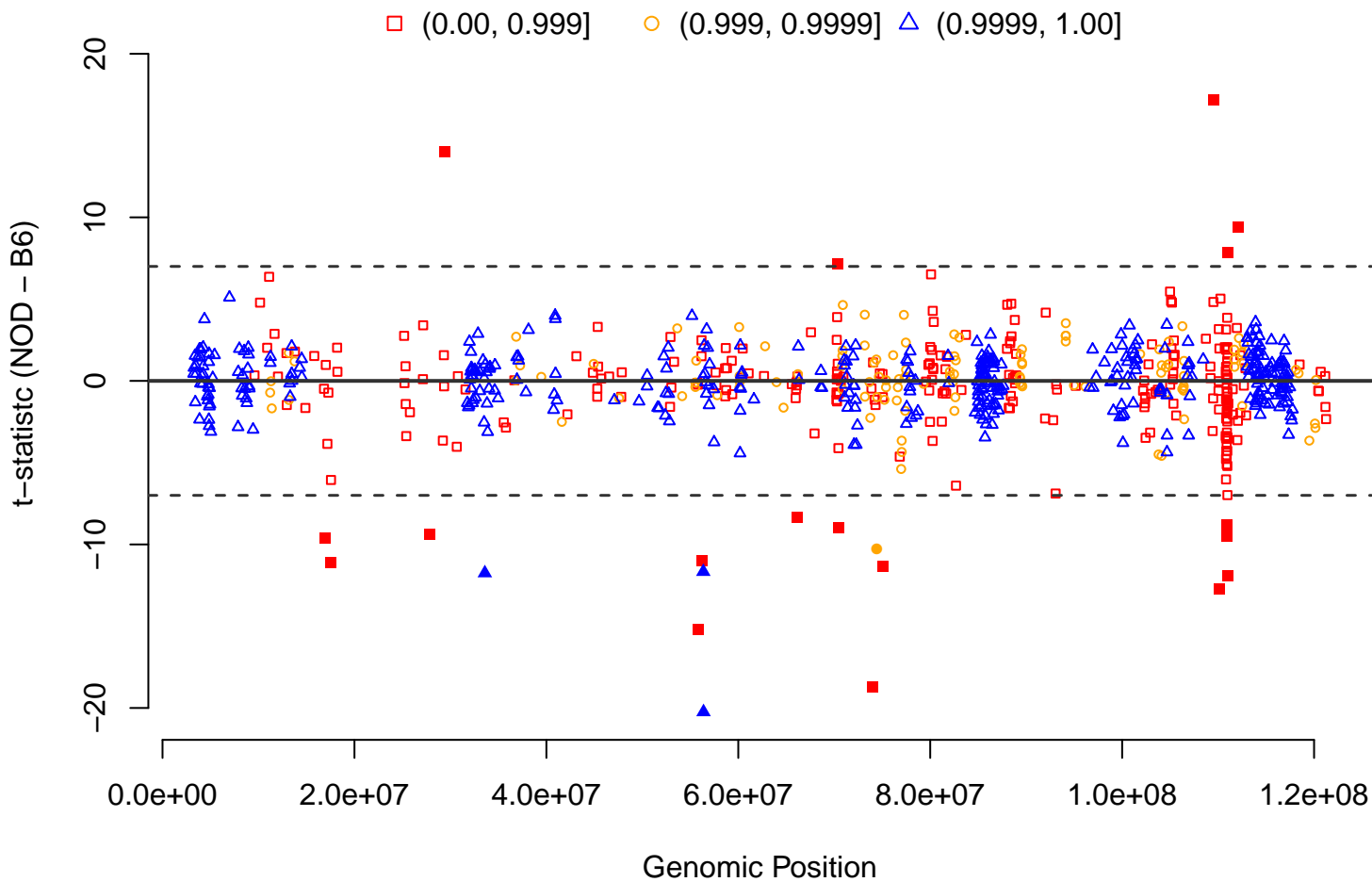

13

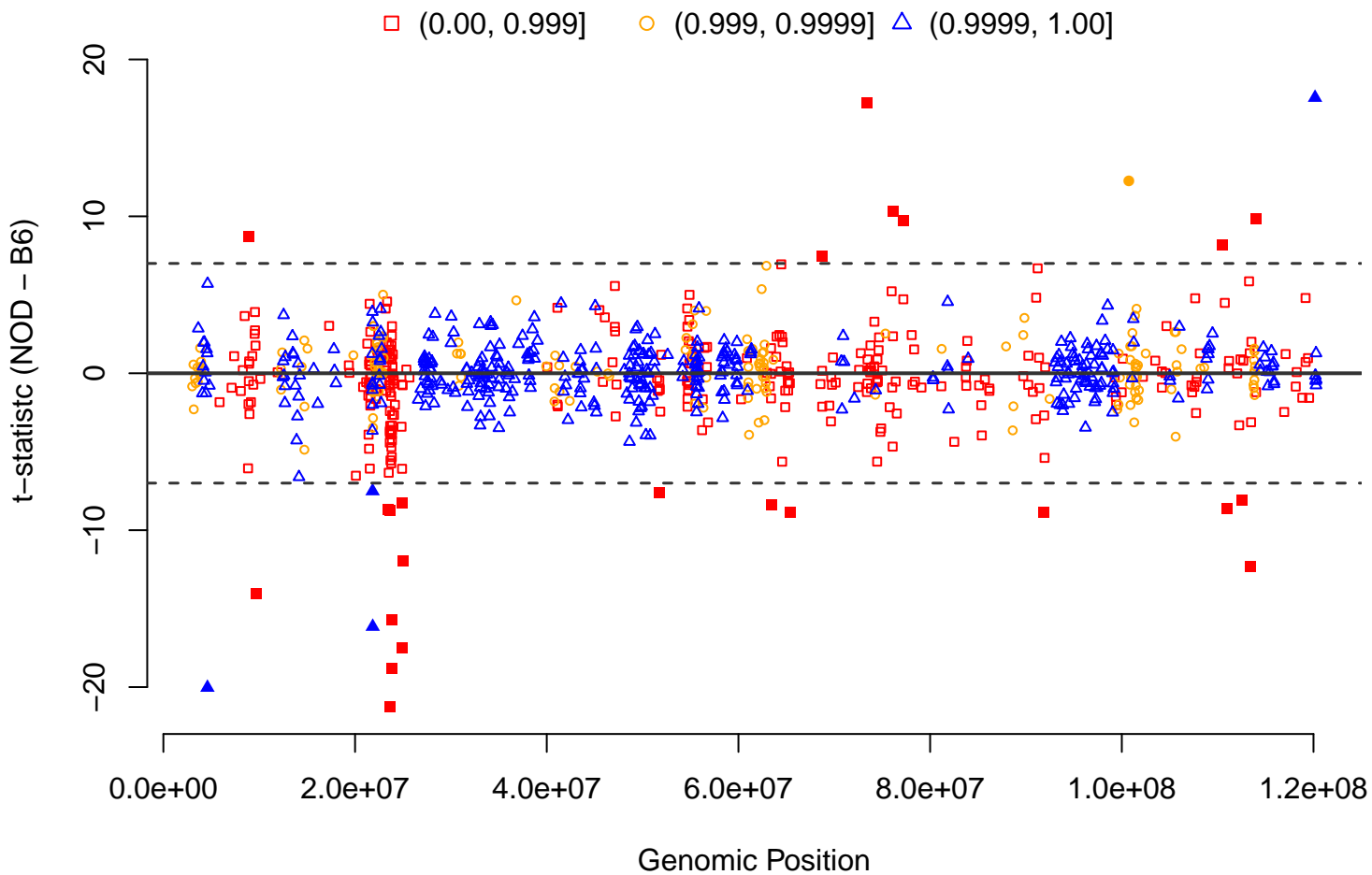

14

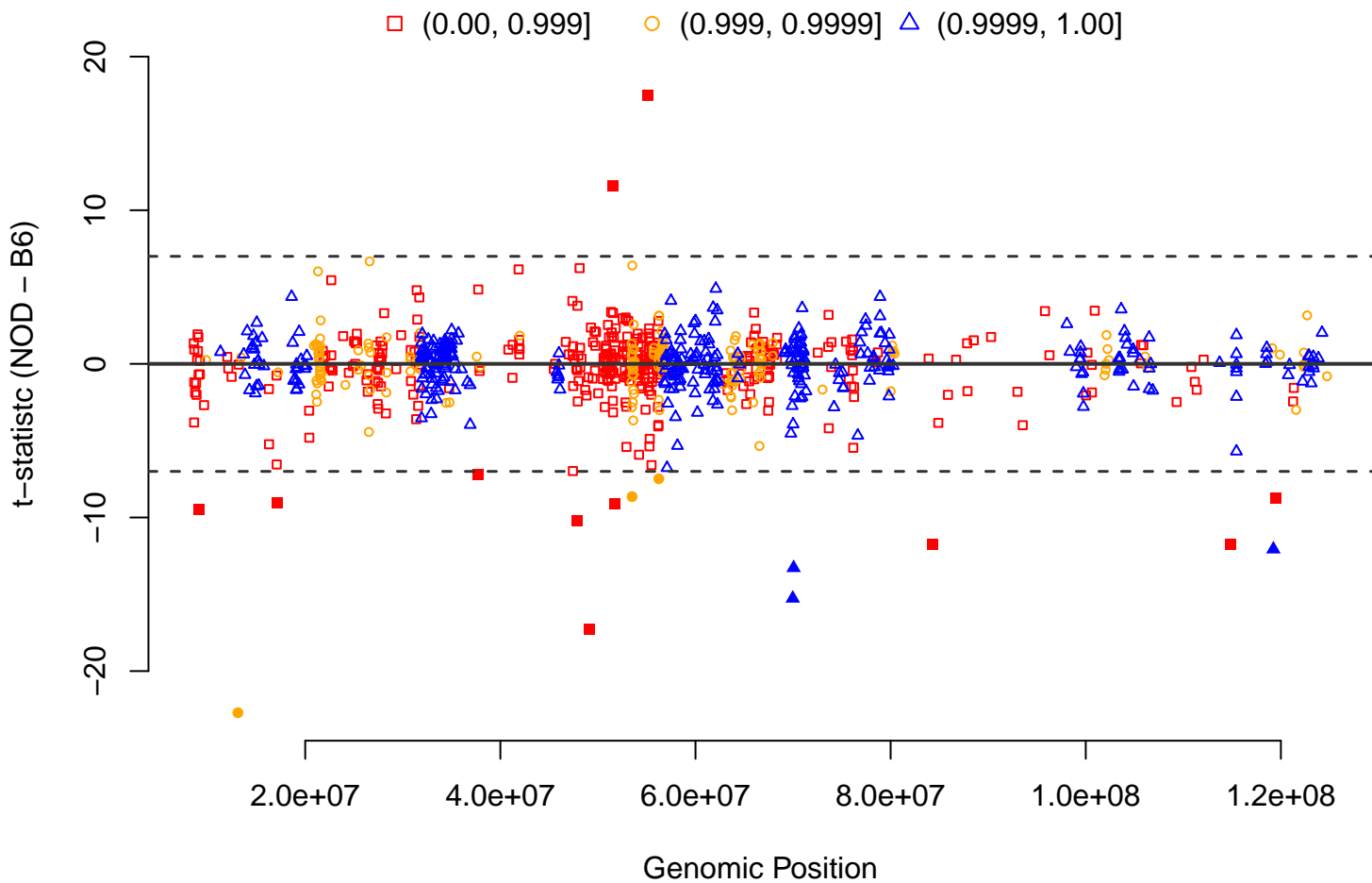

15

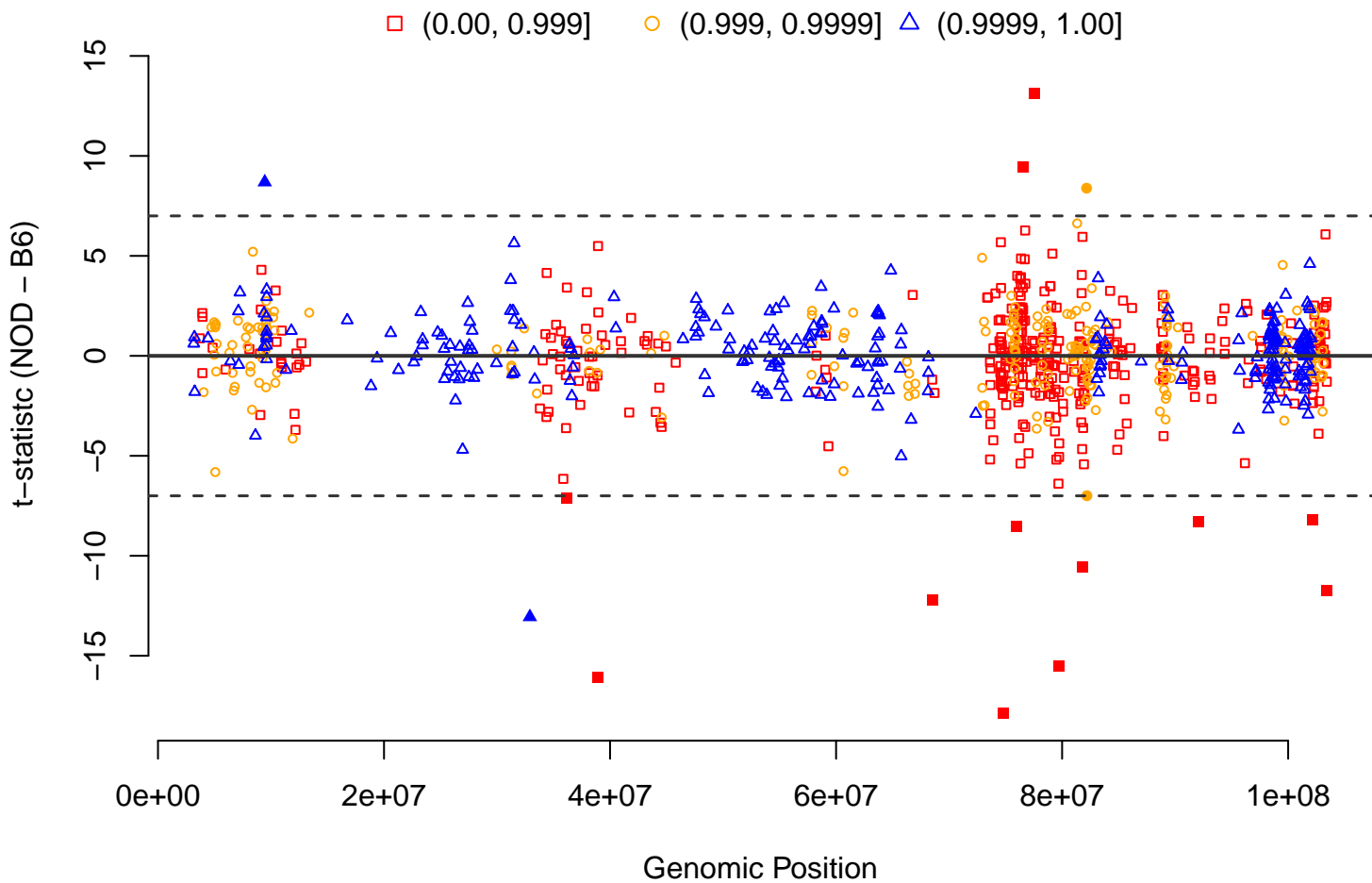

16

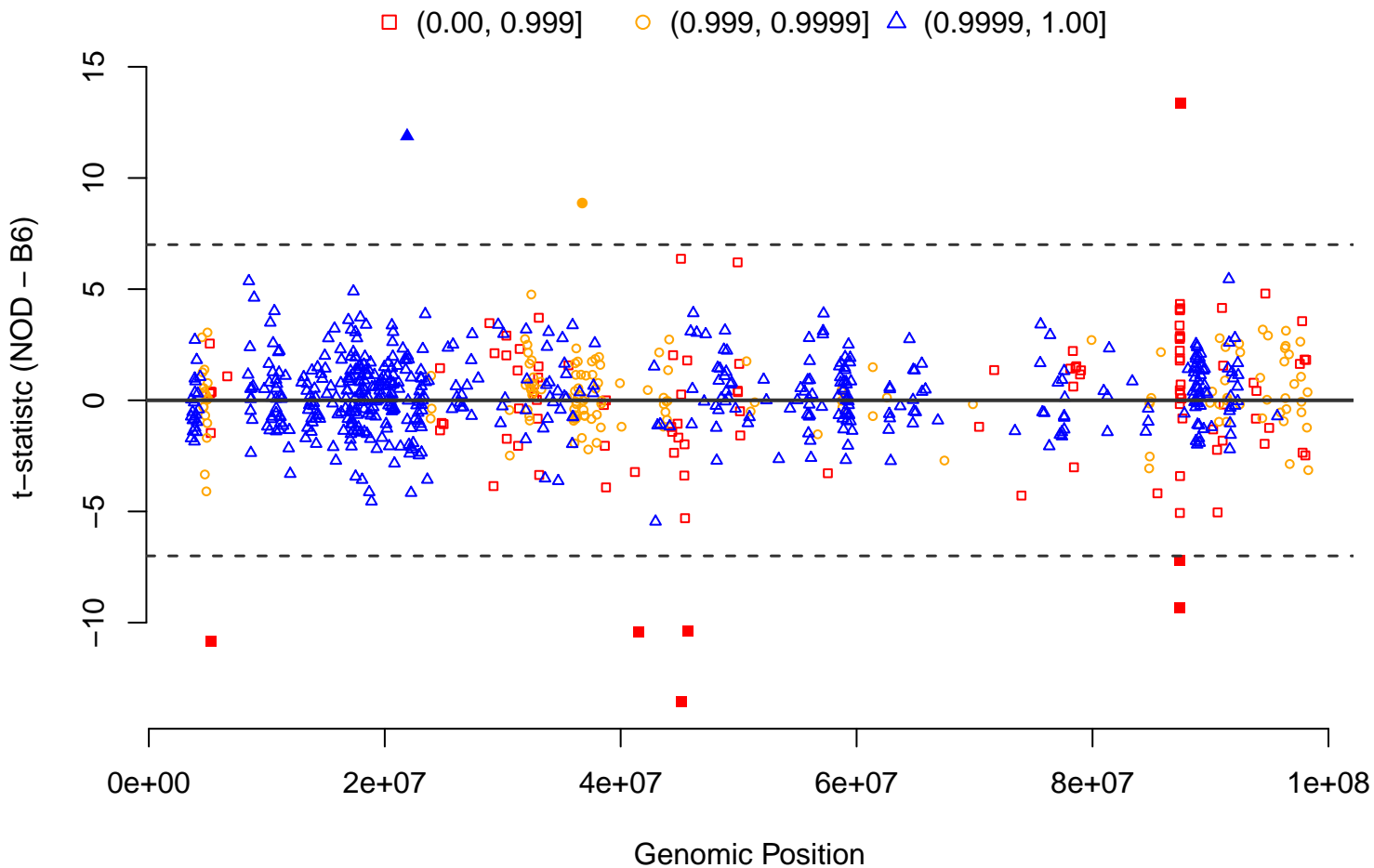

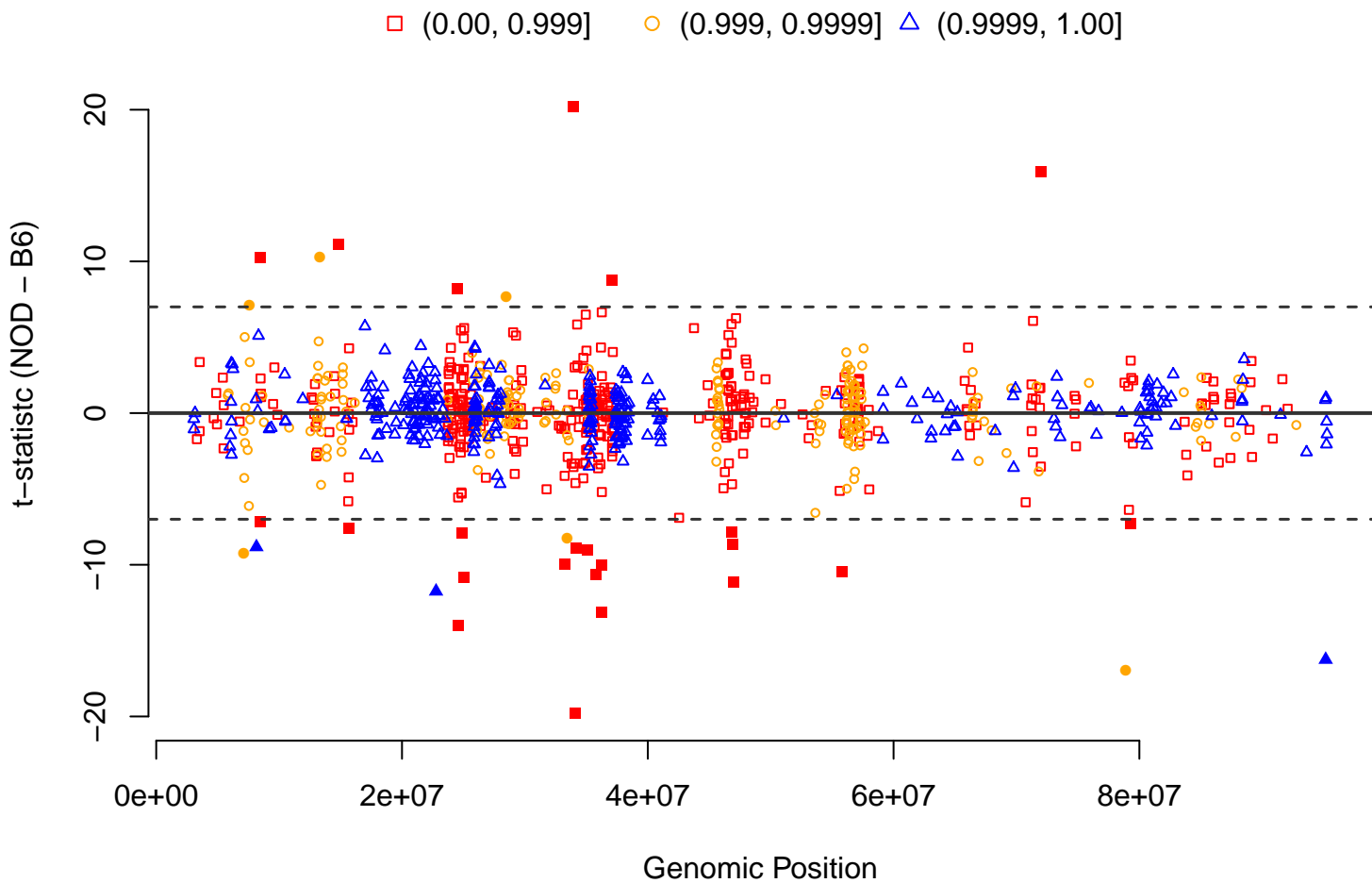

18

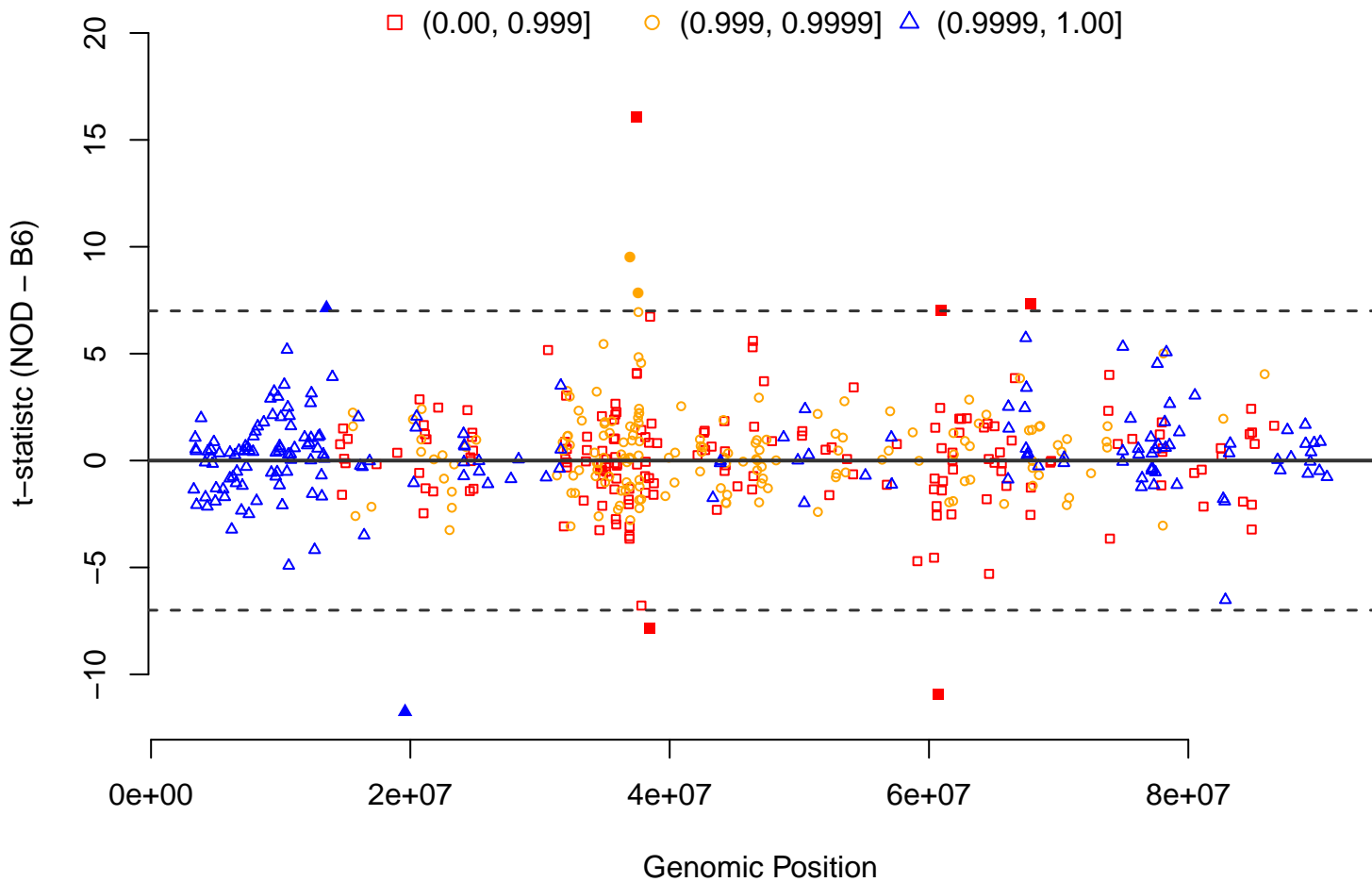

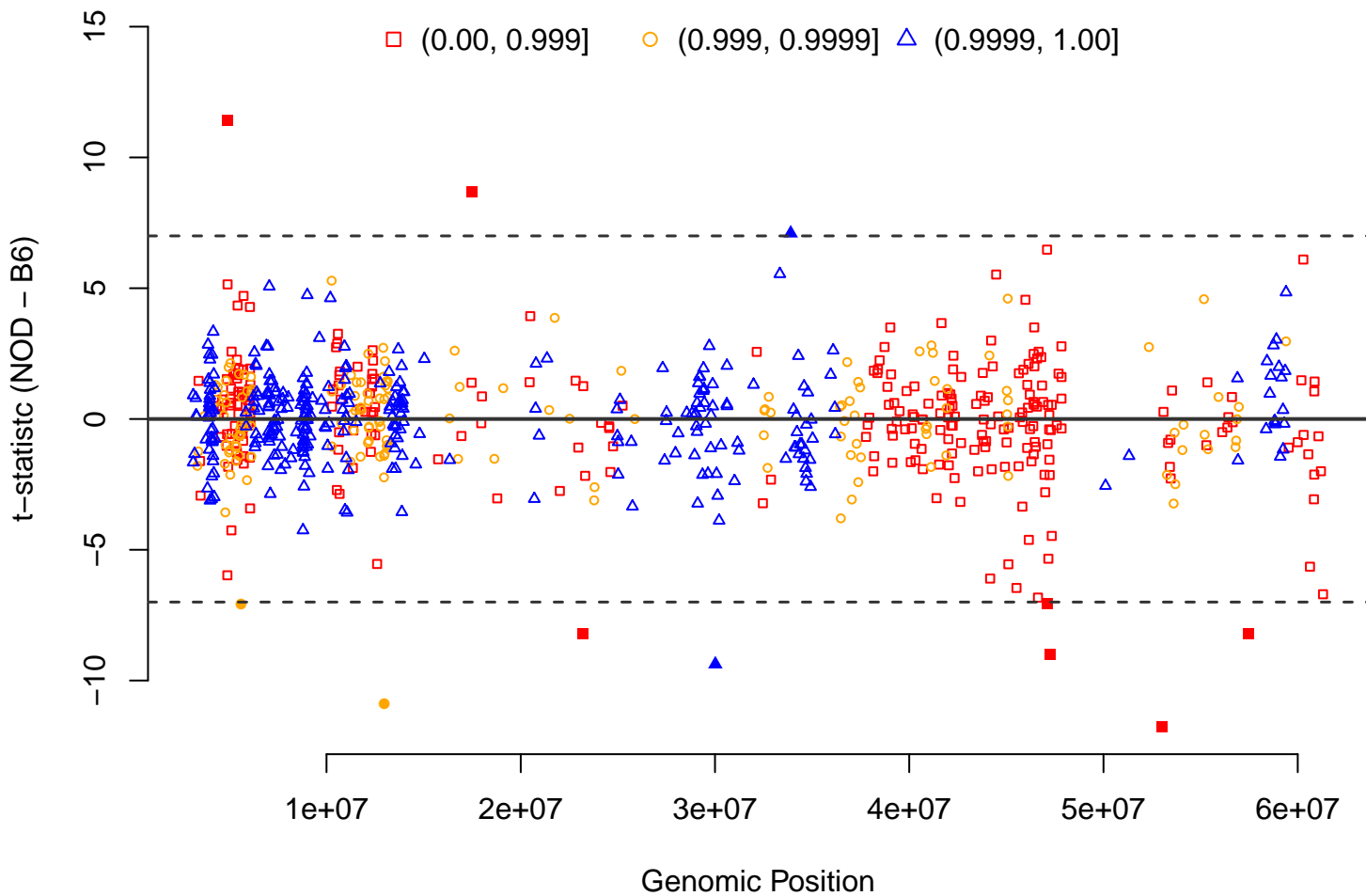

X

□ (0.00, 0.999]    ○ (0.999, 0.9999]    △ (0.9999, 1.00]

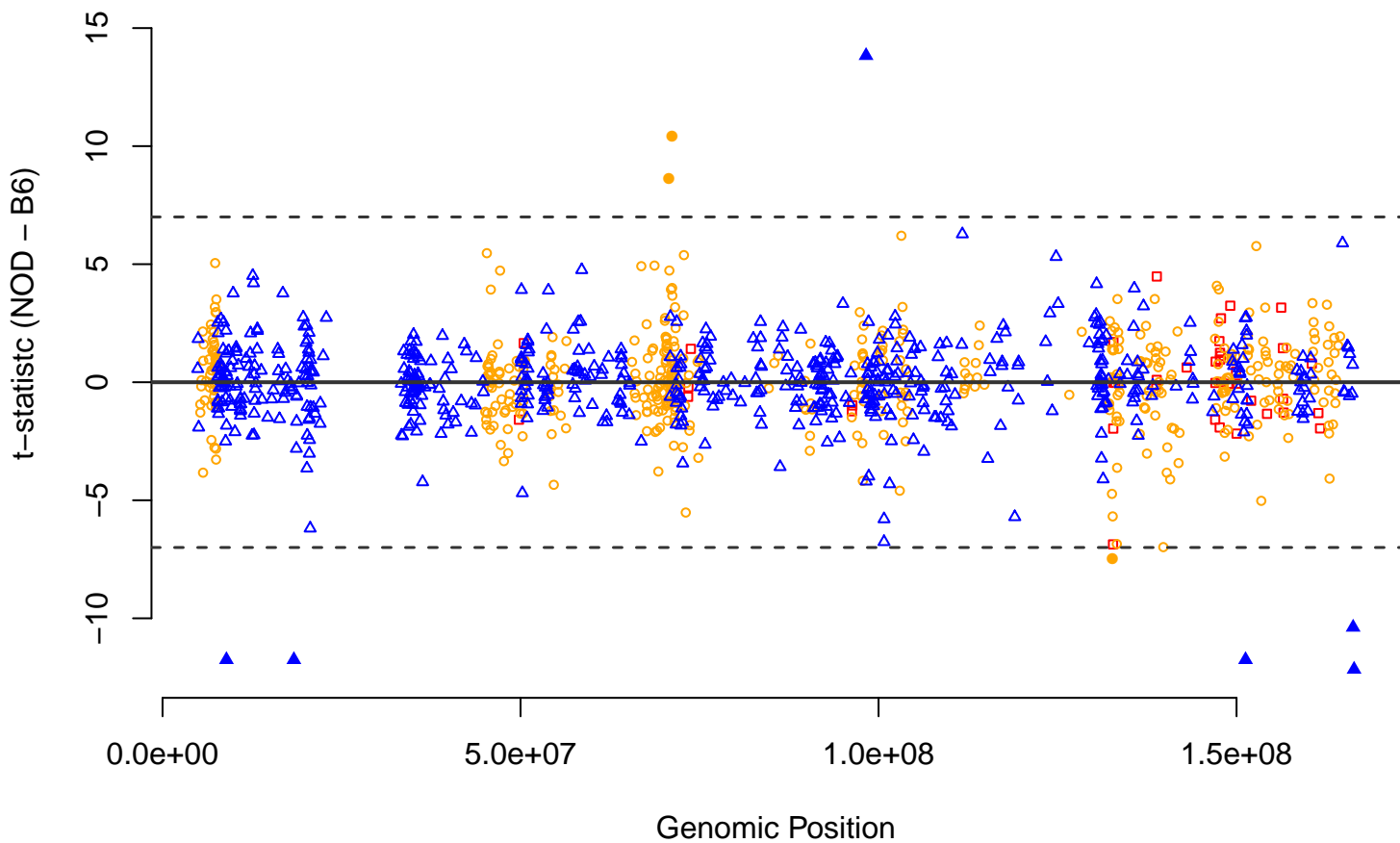

Supplement: Supporting Information [file supp_2.2.203_FigureS13.pdf]

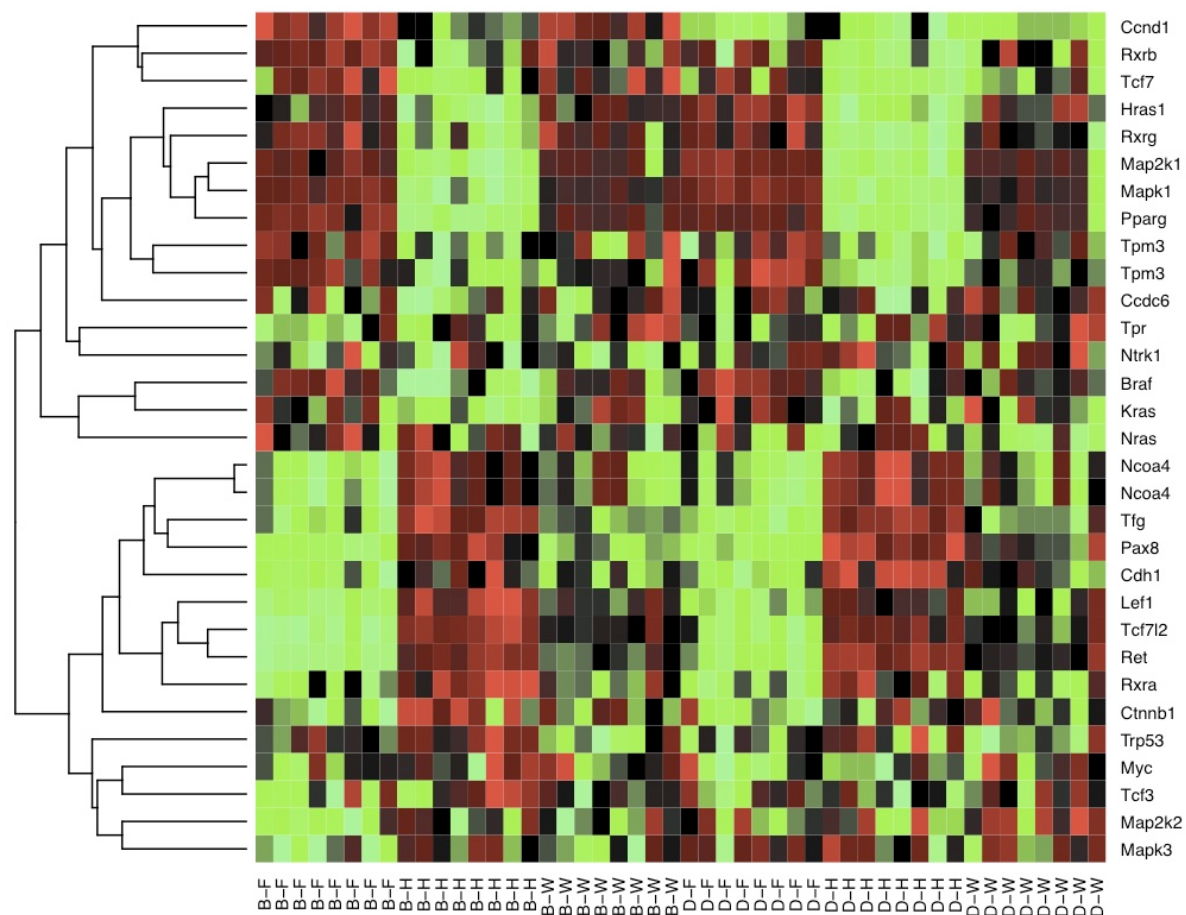

**Figure S14** Heatmap of the expression of pathway "Thyroid cancer".

Supplement: Supporting Information [file supp_2.2.203_FigureS14.pdf]
