# Supplementary material for: Transcriptome Atlases of Mouse Brain Reveals Differential Expression Across Brain Regions and Genetic Backgrounds
Source: G3 (Bethesda). 2012 Feb 1;2(2):203–11. doi: 10.1534/g3.111.001602 (PMC3284328; doi:10.1534/g3.111.001602)
Supplement: Supporting Information [file supp_2.2.203_FigureS15.pdf]

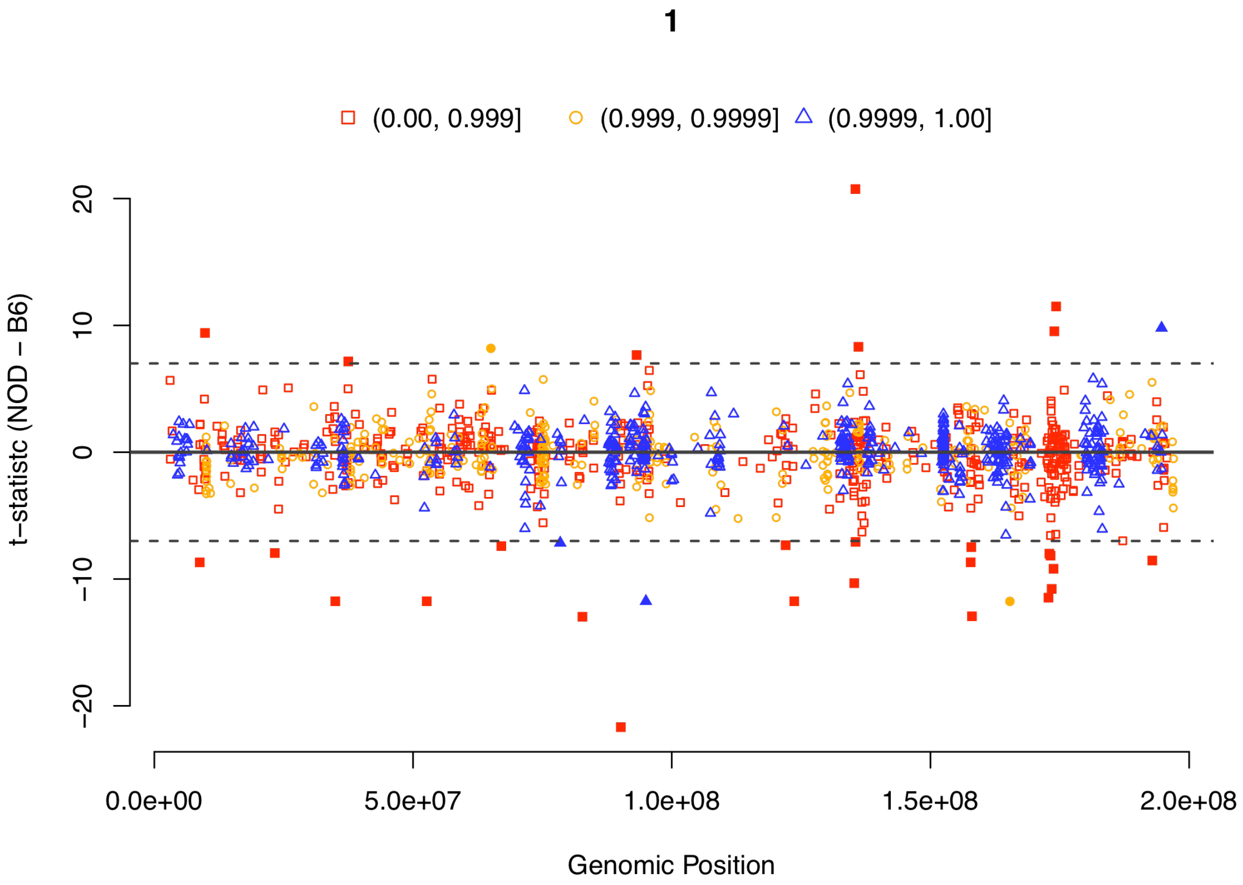

Figure S15 Sequence similarity vs. strain effect in forebrain along chromosomes. This figure shows the relation between strain effect in forebrain and variable levels of sequence similarities along chromosome 1. The X-axis is genomic position by base pair and the Y-axis is the t-statistic comparing the expression in NOD/ShiLtJ vs. C57BL/6J so that positive t-statistics indicate higher expression in NOD. One point indicates one of the 22,963 transcripts with consistent sequence similarity measurement. Different color (as labeled at the top of the figure) indicates different level of sequence similarity. Two horizontal broken lines indicate t-statistics of 7 or -7, which approximately corresponds to p-value 1e-5, the cutoff used in Figure 5 of the main text. Those transcripts with t-statistics beyond these cutoffs are labeled by filled points. Similar figures for other chromosomes can be found in File S2.
