# Supplementary material for: Transcriptome Atlases of Mouse Brain Reveals Differential Expression Across Brain Regions and Genetic Backgrounds
Source: G3 (Bethesda). 2012 Feb 1;2(2):203–11. doi: 10.1534/g3.111.001602 (PMC3284328; doi:10.1534/g3.111.001602)
Supplement: Supporting Information [file supp_2.2.203_FigureS16.pdf]

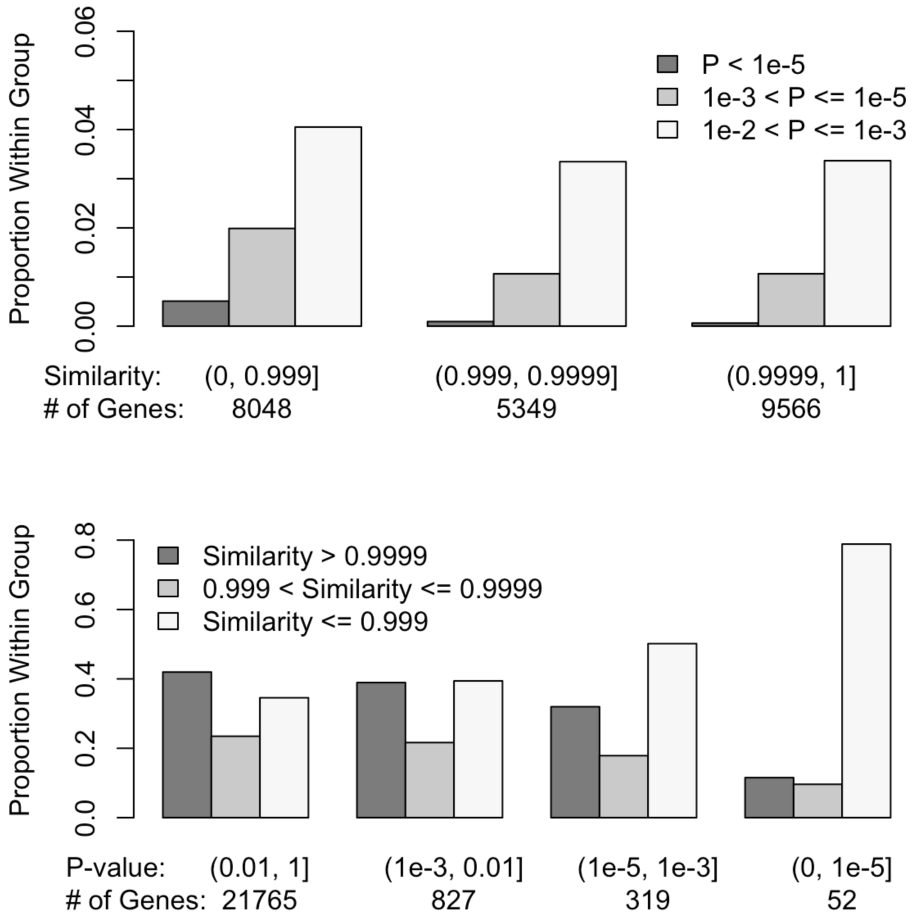

Figure S16 Comparison strain x hindbrain interaction effects vs. DNA similarity. Comparisons of the categories of transcripts based on p-value of strain x hindbrain interaction and DNA similarity, for the 22,963 transcripts with consistent DNA similarity measurements along the gene body. In the upper panel, the transcripts were grouped based on DNA similarity and within each group we compared proportion of transcripts within different p-value ranges. In the lower panel, the transcripts were grouped based on strain x hindbrain interaction effect p-values and within each group we compared the proportion of transcripts with different DNA similarities.
