# Supplementary material for: Transcriptome Atlases of Mouse Brain Reveals Differential Expression Across Brain Regions and Genetic Backgrounds
Source: G3 (Bethesda). 2012 Feb 1;2(2):203–11. doi: 10.1534/g3.111.001602 (PMC3284328; doi:10.1534/g3.111.001602)
Supplement: Supporting Information [file supp_2.2.203_FigureS17.pdf]

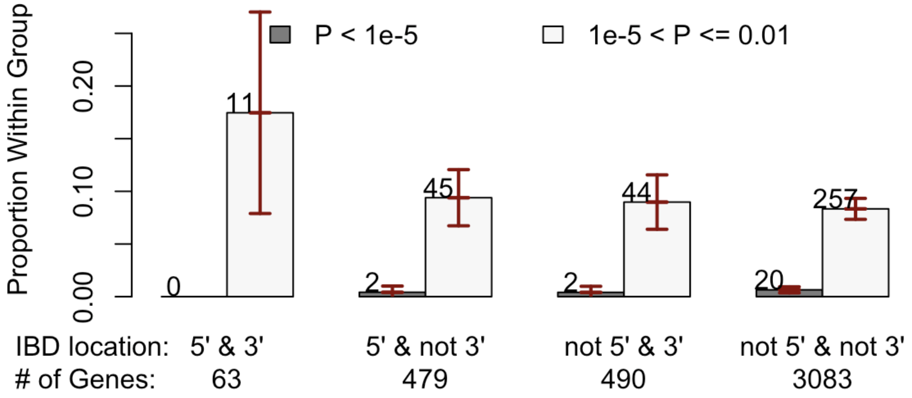

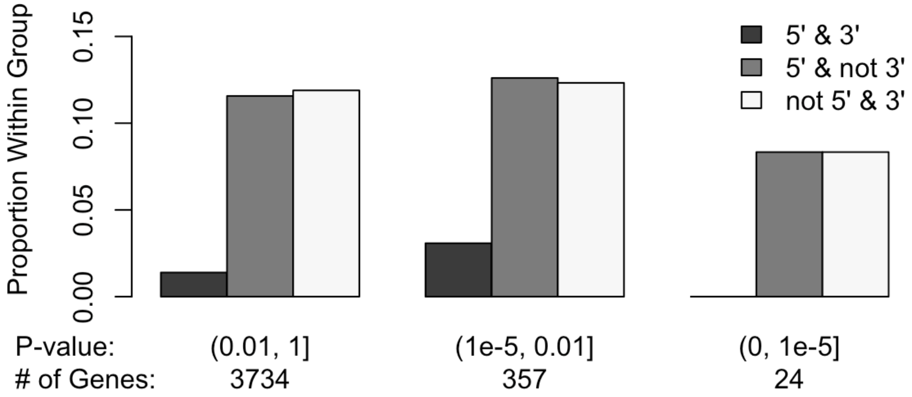

Figure S17 Comparison strain x hindbrain interaction effects vs. DNA similarity at 5' and 3' regions. Comparisons of the categories of transcripts based on p-value of strain x hindbrain interaction and IBD status at 5' and 3' regions, for the 4,118 transcripts with variable DNA similarity measurements in the gene body. Here IBD is defined as DNA similarity > 0.999. In the upper panel, the transcripts were grouped based on IBD location and within each group we compared proportion of transcripts within different p-value ranges. The vertical bars indicated the 95% confidence intervals. In the lower panel, the transcripts were grouped based on strain x hindbrain interaction p-values and within each group we compared the proportion of transcripts with different IBD location statuses.
