# Supplementary material for: Transcriptome Atlases of Mouse Brain Reveals Differential Expression Across Brain Regions and Genetic Backgrounds
Source: G3 (Bethesda). 2012 Feb 1;2(2):203–11. doi: 10.1534/g3.111.001602 (PMC3284328; doi:10.1534/g3.111.001602)
Supplement: Supporting Information [file supp_2.2.203_FigureS12.pdf]

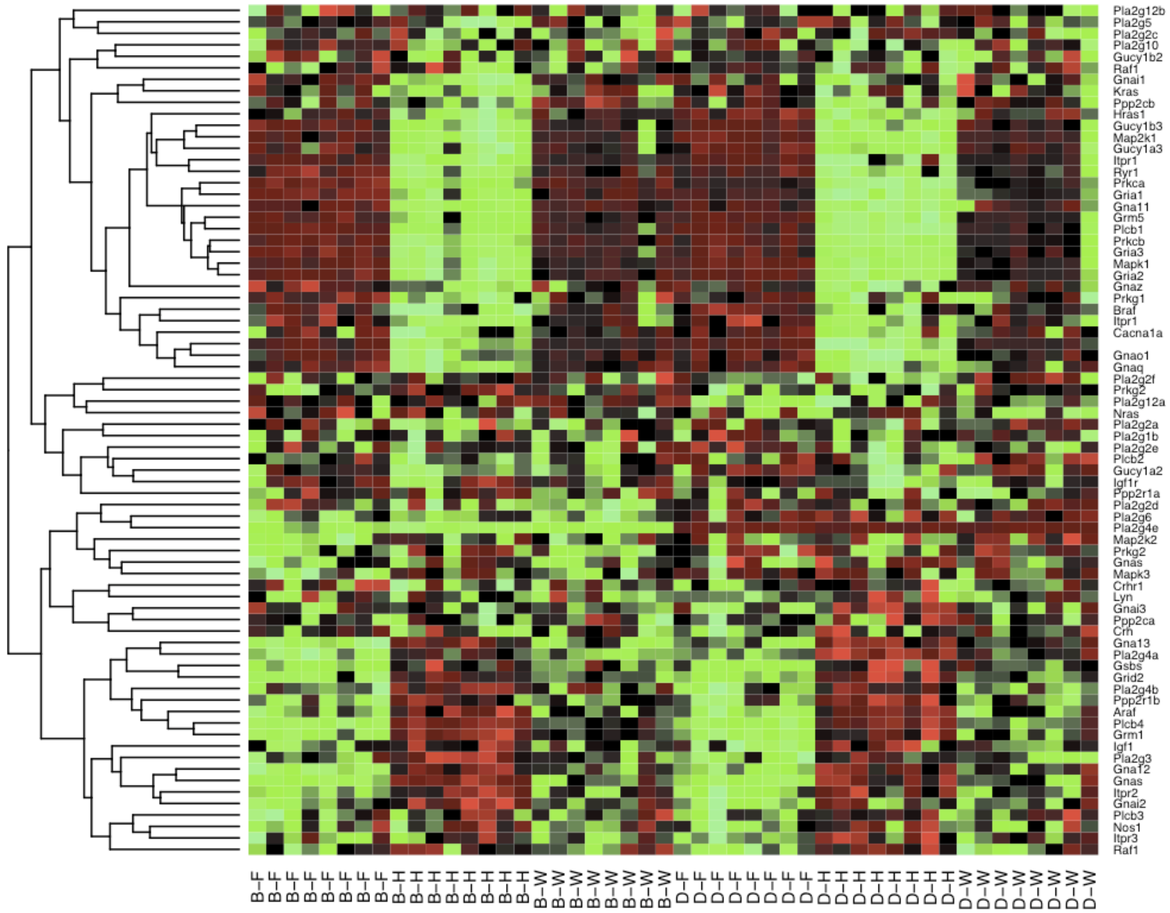

Figure S12 Heatmap of the expression of pathway “Long-term depression”. Heatmap of the expression of the 76 probesets belonging to pathway “Long-term depression”. Each column of the heatmap corresponds to a sample and each row corresponds to a probeset. In the column labels, B and D indicates strain of B6 and NOD respectively, and F, H, and W indicate forebrain, hindbrain and whole brain, respectively.
