# Supplementary material for: Transcriptome Atlases of Mouse Brain Reveals Differential Expression Across Brain Regions and Genetic Backgrounds
Source: G3 (Bethesda). 2012 Feb 1;2(2):203–11. doi: 10.1534/g3.111.001602 (PMC3284328; doi:10.1534/g3.111.001602)
Supplement: Supporting Information [file supp_2.2.203_TableS2.pdf]

Table S2 FDR at different p-value cutoffs for 1.0ST array.

p-value	Forebrain vs. hindbrain			Whole brain vs. forebrain			NOD vs. B6			Clozapine vs. vehicle			Interaction: hindbrain-NOD		
	D	FD	FDR	D	FD	FDR	D	FD	FDR	D	FD	FDR	D	FD	FDR
0.05	7351	888.2	0.121	2877	1235.3	0.429	7446	893.9	0.12	3391	1138.9	0.336	4196	1109.9	0.265
0.04	6837	710.6	0.104	2494	988.2	0.396	6877	715.1	0.104	2898	911.1	0.314	3733	887.9	0.238
0.03	6213	532.9	0.086	2088	741.2	0.355	6275	536.3	0.085	2355	683.3	0.29	3180	665.9	0.209
0.02	5429	355.3	0.065	1623	494.1	0.304	5502	357.6	0.065	1772	455.6	0.257	2585	444	0.172
0.01	**4401**	**177.6**	**0.04**	1125	247.1	0.22	**4465**	**178.8**	**0.04**	1085	227.8	0.21	1803	222	0.123
0.009	4266	159.9	0.037	1080	222.4	0.206	4328	160.9	0.037	1005	205	0.204	1710	199.8	0.117
0.008	4119	142.1	0.034	1010	197.6	0.196	4168	143	0.034	924	182.2	0.197	1619	177.6	0.11
0.007	3964	124.3	0.031	944	172.9	0.183	4015	125.1	0.031	834	159.4	0.191	1527	155.4	0.102
0.006	3776	106.6	0.028	881	148.2	0.168	3860	107.3	0.028	753	136.7	0.182	1406	133.2	0.095
0.005	3610	88.8	0.025	804	123.5	0.154	3696	89.4	0.024	663	113.9	0.172	1300	111	0.085
0.004	3417	71.1	0.021	726	98.8	0.136	3467	71.5	0.021	560	91.1	0.163	1179	88.8	0.075
0.003	3161	53.3	0.017	616	74.1	0.12	3187	53.6	0.017	459	68.3	0.149	1027	66.6	0.065
0.002	2823	35.5	0.013	498	49.4	0.099	2854	35.8	0.013	347	45.6	0.131	**865**	**44.4**	**0.051**
0.001	2351	17.8	0.008	364	24.7	0.068	2437	17.9	0.007	180	22.8	0.127	672	22.2	0.033
9e-04	2285	16	0.007	355	22.2	0.063	2365	16.1	0.007	167	20.5	0.123	652	20	0.031
8e-04	2212	14.2	0.006	337	19.8	0.059	2295	14.3	0.006	153	18.2	0.119	622	17.8	0.029
7e-04	2127	12.4	0.006	316	17.3	0.055	2233	12.5	0.006	135	15.9	0.118	588	15.5	0.026
6e-04	2034	10.7	0.005	**293**	**14.8**	**0.051**	2147	10.7	0.005	113	13.7	0.121	555	13.3	0.024
5e-04	1937	8.9	0.005	273	12.4	0.045	2051	8.9	0.004	92	11.4	0.124	519	11.1	0.021
4e-04	1811	7.1	0.004	259	9.9	0.038	1957	7.2	0.004	79	9.1	0.115	482	8.9	0.018
3e-04	1669	5.3	0.003	231	7.4	0.032	1828	5.4	0.003	61	6.8	0.111	438	6.7	0.015
2e-04	1521	3.6	0.002	203	4.9	0.024	1652	3.6	0.002	41	4.6	0.112	378	4.4	0.012
1e-04	1287	1.8	0.001	140	2.5	0.018	1386	1.8	0.001	23	2.3	0.1	310	2.2	0.007

For a p-value cutoff p, the expected number of false discoveries (FD) is $\pi 0Np$ (N is the total number of genes), the number of discoveries (D) was the number of genes with p-values < p, and FDR is FD/D.
