# Supplementary material for: Transcriptome Atlases of Mouse Brain Reveals Differential Expression Across Brain Regions and Genetic Backgrounds
Source: G3 (Bethesda). 2012 Feb 1;2(2):203–11. doi: 10.1534/g3.111.001602 (PMC3284328; doi:10.1534/g3.111.001602)
Supplement: Supporting Information [file supp_2.2.203_TableS3.pdf]

Table S3   The 122 GO terms associated with brain region effects

GO ID	GO type	Size	GO Term Name	P-val F vs. H	P-val W vs. H
GO:0006887	BP	109	exocytosis	0	0
GO:0008283	BP	737	cell proliferation	0	0
GO:0016192	BP	446	vesicle-mediated transport	0	0
GO:0019226	BP	250	transmission of nerve impulse	0	0
GO:0005262	MF	70	calcium channel activity	0	0
GO:0007155	BP	614	cell adhesion	0	2e-04
GO:0022610	BP	614	biological adhesion	0	2e-04
GO:0048489	BP	28	synaptic vesicle transport	0	2e-04
GO:0000267	CC	607	cell fraction	0	2e-04
GO:0042391	BP	108	regulation of membrane potential	0	4e-04
GO:0051128	BP	281	regulation of cellular component organization	0	4e-04
GO:0007275	BP	2721	multicellular organismal development	0	6e-04
GO:0021987	BP	36	cerebral cortex development	0	6e-04
GO:0016788	MF	666	hydrolase activity, acting on ester bonds	0	6e-04
GO:0042578	MF	324	phosphoric ester hydrolase activity	0	6e-04
GO:0007267	BP	336	cell-cell signaling	0	8e-04
GO:0016790	MF	102	thiolester hydrolase activity	0	8e-04
GO:0031344	BP	54	regulation of cell projection organization	0	0.001
GO:0048869	BP	1765	cellular developmental process	0	0.001
GO:0008066	MF	29	glutamate receptor activity	0	0.001
GO:0016044	BP	276	cellular membrane organization	0	0.0014
GO:0042063	BP	50	gliogenesis	0	0.0014
GO:0007156	BP	78	homophilic cell adhesion	0	0.0016
GO:0022603	BP	200	regulation of anatomical structure morphogenesis	0	0.0018
GO:0045211	CC	126	postsynaptic membrane	0	0.002
GO:0006575	BP	159	cellular amino acid derivative metabolic process	0	0.0022
GO:0022604	BP	98	regulation of cell morphogenesis	0	0.0024
GO:0006897	BP	189	endocytosis	0	0.0028
GO:0010324	BP	189	membrane invagination	0	0.0028
GO:0050770	BP	39	regulation of axonogenesis	0	0.0032
GO:0051239	BP	767	regulation of multicellular organismal process	0	0.0032
GO:0008081	MF	69	phosphoric diester hydrolase activity	0	0.0032
GO:0006576	BP	102	cellular biogenic amine metabolic process	0	0.0042
GO:0007270	BP	37	nerve-nerve synaptic transmission	0	0.0048
GO:0048856	BP	2274	anatomical structure development	0	0.0048

GO:0048468	BP	801	cell development	0	0.0056
GO:0048168	BP	21	regulation of neuronal synaptic plasticity	0	0.0074
GO:0051493	BP	89	regulation of cytoskeleton organization	0	0.0076
GO:0030041	BP	53	actin filament polymerization	0	0.0086
GO:0016337	BP	178	cell-cell adhesion	0	0.009
GO:0004620	MF	61	phospholipase activity	0	0.0096
GO:0005267	MF	132	potassium channel activity	0	0.0126
GO:0000902	BP	360	cell morphogenesis	0	0.0134
GO:0008092	MF	395	cytoskeletal protein binding	0	0.0146
GO:0048646	BP	355	anatomical structure formation involved in morphogenesis	0	0.0148
GO:0032989	BP	398	cellular component morphogenesis	0	0.015
GO:0065003	BP	330	macromolecular complex assembly	0	0.0162
GO:0005886	CC	2802	plasma membrane	0	0.0182
GO:0044459	CC	1345	plasma membrane part	0	0.0186
GO:0007015	BP	95	actin filament organization	0	0.0188
GO:0030036	BP	186	actin cytoskeleton organization	0	0.022
GO:0003779	MF	290	actin binding	0	0.026
GO:0034622	BP	275	cellular macromolecular complex assembly	0	0.027
GO:0021954	BP	33	central nervous system neuron development	0	0.0308
GO:0032956	BP	59	regulation of actin cytoskeleton organization	0	0.0316
GO:0030029	BP	199	actin filament-based process	0	0.0336
GO:0010926	BP	791		0	0.0346
GO:0016298	MF	82	lipase activity	0	0.035
GO:0060284	BP	145	regulation of cell development	0	0.0354
GO:0019199	MF	69	transmembrane receptor protein kinase activity	0	0.0404
GO:0030155	BP	86	regulation of cell adhesion	0	0.0438
GO:0022607	BP	486	cellular component assembly	0	0.0448
GO:0051960	BP	138	regulation of nervous system development	0	0.0468
GO:0001725	CC	21	stress fiber	0	0.0516
GO:0004714	MF	56	transmembrane receptor protein tyrosine kinase activity	0	0.052
GO:0001525	BP	147	angiogenesis	0	0.0554
GO:0050767	BP	124	regulation of neurogenesis	0	0.0592
GO:0017016	MF	61	Ras GTPase binding	0	0.0638
GO:0007519	BP	91	skeletal muscle tissue development	0	0.0642
GO:0060538	BP	92	skeletal muscle organ development	0	0.0684
GO:0045664	BP	99	regulation of neuron differentiation	0	0.0704
GO:0017048	MF	22	Rho GTPase binding	0	0.1924
GO:0044456	CC	197	synapse part	2e-04	0
GO:0004721	MF	168	phosphoprotein phosphatase activity	4e-04	0

GO:0007268	BP	209	synaptic transmission	6e-04	0
GO:0032879	BP	361	regulation of localization	6e-04	0
GO:0070838	BP	140	divalent metal ion transport	6e-04	0
GO:0030425	CC	63	dendrite	8e-04	0
GO:0016791	MF	256	phosphatase activity	8e-04	0
GO:0006816	BP	137	calcium ion transport	0.001	0
GO:0044057	BP	162	regulation of system process	0.001	0
GO:0006461	BP	196	protein complex assembly	0.0012	0
GO:0070271	BP	196	protein complex biogenesis	0.0012	0
GO:0001505	BP	72	regulation of neurotransmitter levels	0.0016	0
GO:0007269	BP	48	neurotransmitter secretion	0.0018	0
GO:0016358	BP	48	dendrite development	0.0022	0
GO:0051049	BP	212	regulation of transport	0.0032	0
GO:0030665	CC	25	clathrin coated vesicle membrane	0.0032	0
GO:0050673	BP	79	epithelial cell proliferation	0.0034	0
GO:0046903	BP	264	secretion	0.004	0
GO:0032940	BP	235	secretion by cell	0.0042	0
GO:0044448	CC	65	cell cortex part	0.0042	0
GO:0051641	BP	732	cellular localization	0.0044	0
GO:0043005	CC	218	neuron projection	0.0044	0
GO:0016311	BP	148	dephosphorylation	0.0066	0
GO:0043112	BP	29	receptor metabolic process	0.0074	0
GO:0043687	BP	1117	post-translational protein modification	0.0106	0
GO:0030136	CC	75	clathrin-coated vesicle	0.0106	0
GO:0005097	MF	43	Rab GTPase activator activity	0.0108	0
GO:0050768	BP	39	negative regulation of neurogenesis	0.012	0
GO:0005516	MF	112	calmodulin binding	0.0124	0
GO:0003001	BP	119	generation of a signal involved in cell-cell signaling	0.0126	0
GO:0051046	BP	96	regulation of secretion	0.0128	0
GO:0005057	MF	93	receptor signaling protein activity	0.0136	0
GO:0008021	CC	59	synaptic vesicle	0.0152	0
GO:0046474	BP	45	glycerophospholipid biosynthetic process	0.0184	0
GO:0030662	CC	38	coated vesicle membrane	0.0184	0
GO:0030135	CC	90	coated vesicle	0.0194	0
GO:0060341	BP	117	regulation of cellular localization	0.0226	0
GO:0016529	CC	34	sarcoplasmic reticulum	0.027	0
GO:0009056	BP	1528	catabolic process	0.03	0
GO:0003713	MF	72	transcription coactivator activity	0.0312	0
GO:0016528	CC	36	sarcoplasm	0.0356	0
GO:0044248	BP	890	cellular catabolic process	0.0392	0

GO:0007416	BP	24	synapse assembly	0.0478	0
GO:0048873	BP	23	homeostasis of number of cells within a tissue	0.0716	0
GO:0017157	BP	24	regulation of exocytosis	0.1088	0
GO:0044444	CC	4121	cytoplasmic part	0.1206	0
GO:0016569	BP	68	covalent chromatin modification	0.1242	0
GO:0016570	BP	64	histone modification	0.1392	0
GO:0048872	BP	137	homeostasis of number of cells	0.1614	0
GO:0001894	BP	56	tissue homeostasis	0.1778	0

The columns “P-val F vs. H” and “P-val W vs. H” are the p-values comparing the expression of forebrain vs. hindbrain, and comparing whole brain vs. hindbrain, respectively.
