# Supplementary material for: Transcriptome Atlases of Mouse Brain Reveals Differential Expression Across Brain Regions and Genetic Backgrounds
Source: G3 (Bethesda). 2012 Feb 1;2(2):203–11. doi: 10.1534/g3.111.001602 (PMC3284328; doi:10.1534/g3.111.001602)
Supplement: Supporting Information [file supp_2.2.203_TableS4.pdf]

Table S4 The 41 GO terms associated with strain effect in hindbrain.

GO ID	GO type	GO name	Size	P-value
GO:0000323	CC	lytic vacuole	160	0
GO:0005764	CC	lysosome	160	0
GO:0044456	CC	synapse part	197	0
GO:0008076	CC	voltage-gated potassium channel complex	57	0
GO:0034705	CC	potassium channel complex	57	0
GO:0030135	CC	coated vesicle	90	0
GO:0030136	CC	clathrin-coated vesicle	75	0
GO:0005789	CC	endoplasmic reticulum membrane	99	0
GO:0008021	CC	synaptic vesicle	59	0
GO:0005768	CC	endosome	242	8.00e-04
GO:0045202	CC	synapse	311	8.00e-04
GO:0044432	CC	endoplasmic reticulum part	118	8.00e-04
GO:0042175	CC	nuclear envelope-endoplasmic reticulum network	103	0.0012
GO:0044463	CC	cell projection part	103	0.0016
GO:0005773	CC	vacuole	187	0.0016
GO:0045211	CC	postsynaptic membrane	126	0.0016
GO:0031988	CC	membrane-bounded vesicle	251	0.002
GO:0016023	CC	cytoplasmic membrane-bounded vesicle	244	0.002
GO:0043025	CC	neuronal cell body	84	0.002
GO:0005856	CC	cytoskeleton	1008	0.0029
GO:0005097	MF	Rab GTPase activator activity	43	0
GO:0016301	MF	kinase activity	846	0
GO:0016627	MF	oxidoreductase activity, acting on the CH-CH group of donors	48	0
GO:0051536	MF	iron-sulfur cluster binding	48	0
GO:0051540	MF	metal cluster binding	48	0
GO:0005509	MF	calcium ion binding	829	0
GO:0046873	MF	metal ion transmembrane transporter activity	292	0
GO:0031406	MF	carboxylic acid binding	69	0
GO:0005215	MF	transporter activity	1048	0
GO:0016772	MF	transferase activity, transferring phosphorus-containing groups	981	4.00e-04
GO:0019842	MF	vitamin binding	119	4.00e-04
GO:0016597	MF	amino acid binding	25	4.00e-04
GO:0016773	MF	phosphotransferase activity, alcohol group as acceptor	698	8.00e-04
GO:0005267	MF	potassium channel activity	132	8.00e-04
GO:0005249	MF	voltage-gated potassium channel activity	85	8.00e-04
GO:0022843	MF	voltage-gated cation channel activity	132	8.00e-04
GO:0005261	MF	cation channel activity	252	8.00e-04
