# Supplementary material for: Transcriptome Atlases of Mouse Brain Reveals Differential Expression Across Brain Regions and Genetic Backgrounds
Source: G3 (Bethesda). 2012 Feb 1;2(2):203–11. doi: 10.1534/g3.111.001602 (PMC3284328; doi:10.1534/g3.111.001602)
Supplement: Supporting Information [file supp_2.2.203_TableS5.pdf]

Table S5 The 35 pathways associated with brain region effect.

Pathway ID	Size	Pathway Name	P-val F vs. H	P-val W vs. H
05218	75	Melanoma	2e-04	0.0016
04720	77	Long-term potentiation	8e-04	2e-04
00562	55	Inositol phosphate metabolism	8e-04	0.0036
05216	31	Thyroid cancer	0.0018	0.0028
04730	76	Long-term depression	0.0026	0
04020	204	Calcium signaling pathway	0.003	0
04270	133	Vascular smooth muscle contraction	0.0036	0.0024
04810	224	Regulation of actin cytoskeleton	0	0.0064
04360	140	Axon guidance	0	0.0116
00500	36	Starch and sucrose metabolism	0	0.2154
04540	95	Gap junction	6e-04	0.0088
04520	87	Adherens junction	8e-04	0.0228
01040	28	Biosynthesis of unsaturated fatty acids	0.001	0.0368
04510	209	Focal adhesion	0.0014	0.0306
04320	22	Dorso-ventral axis formation	0.0016	0.0536
00512	29	O-Glycan biosynthesis	0.0026	0.2054
04010	287	MAPK signaling pathway	0.0028	0.0056
04920	71	Adipocytokine signaling pathway	0.003	0.116
04070	79	Phosphatidylinositol signaling system	0.0036	0.0042
04080	260	Neuroactive ligand-receptor interaction	0.004	0.0142
00380	41	Tryptophan metabolism	0.004	0.0672
05412	83	Arrhythmogenic right ventricular cardiomyopathy (ARVC)	0.0048	4e-04
04916	105	Melanogenesis	0.0076	4e-04
04912	105	GnRH signaling pathway	0.0084	0.0034
00564	70	Glycerophospholipid metabolism	0.0104	0.001
05214	70	Glioma	0.0166	0.0022
04144	221	Endocytosis	0.0188	0.0036
00563	25	Glycosylphosphatidylinositol(GPI)-anchor biosynthesis	0.0206	0.001
04512	83	ECM-receptor interaction	0.0222	0.002
05410	92	Hypertrophic cardiomyopathy (HCM)	0.0408	0.0012
00620	50	Pyruvate metabolism	0.044	0.0016
00310	45	Lysine degradation	0.072	0
04260	89	Cardiac muscle contraction	0.1026	0.0014
04514	159	Cell adhesion molecules (CAMs)	0.1278	0.0026
04662	90	B cell receptor signaling pathway	0.5024	0.0034

The columns “P-val F vs. H” and “P-val W vs. H” are the p-values comparing the expression of forebrain vs. hindbrain, and comparing whole brain vs. hindbrain, respectively.
