# Supplementary material for: Transcriptome Atlases of Mouse Brain Reveals Differential Expression Across Brain Regions and Genetic Backgrounds
Source: G3 (Bethesda). 2012 Feb 1;2(2):203–11. doi: 10.1534/g3.111.001602 (PMC3284328; doi:10.1534/g3.111.001602)
Supplement: Supporting Information [file supp_2.2.203_TableS1.pdf]

Tables

Table S1 FDR at different p-value cutoffs for 1.1ST array.

p-value	Forebrain vs. hindbrain			Whole brain vs. forebrain			NOD vs. B6			Clozapine vs. vehicle			Interaction: hindbrain-NOD		
	D	FD	FDR	D	FD	FDR	D	FD	FDR	D	FD	FDR	D	FD	FDR
0.05	11141	680.5	0.061	4128	1093.9	0.265	6666	943.4	0.142	2418	1259	0.521	4071	1104.7	0.271
0.04	**10570**	**544.4**	**0.052**	3654	875.1	0.239	6099	754.7	0.124	2010	1007.2	0.501	3564	883.8	0.248
0.03	9910	408.3	0.041	3120	656.3	0.21	5495	566	0.103	1575	755.4	0.48	3032	662.8	0.219
0.02	9102	272.2	0.03	2508	437.6	0.174	4784	377.4	0.079	1125	503.6	0.448	2457	441.9	0.18
0.01	7954	136.1	0.017	1745	218.8	0.125	**3896**	**188.7**	**0.048**	638	251.8	0.395	1697	220.9	0.13
0.009	7810	122.5	0.016	1646	196.9	0.12	3759	169.8	0.045	588	226.6	0.385	1596	198.8	0.125
0.008	7648	108.9	0.014	1562	175	0.112	3628	150.9	0.042	530	201.4	0.38	1506	176.8	0.117
0.007	7455	95.3	0.013	1468	153.1	0.104	3496	132.1	0.038	472	176.3	0.374	1411	154.7	0.11
0.006	7219	81.7	0.011	1372	131.3	0.096	3338	113.2	0.034	412	151.1	0.367	1304	132.6	0.102
0.005	7005	68.0	0.01	1245	109.4	0.088	3178	94.3	0.03	346	125.9	0.364	1195	110.5	0.092
0.004	6748	54.4	0.008	1103	87.5	0.079	3029	75.5	0.025	274	100.7	0.368	1065	88.4	0.083
0.003	6403	40.8	0.006	993	65.6	0.066	2757	56.6	0.021	200	75.5	0.378	919	66.3	0.072
0.002	5976	27.2	0.005	**834**	**43.8**	**0.053**	2470	37.7	0.015	142	50.4	0.355	**778**	**44.2**	**0.057**
0.001	5330	13.6	0.003	625	21.9	0.035	2087	18.9	0.009	61	25.2	0.413	602	22.1	0.037
9e-04	5245	12.2	0.002	593	19.7	0.033	2040	17	0.008	53	22.7	0.428	579	19.9	0.034
8e-04	5135	10.9	0.002	563	17.5	0.031	1975	15.1	0.008	48	20.1	0.419	551	17.7	0.032
7e-04	5026	9.5	0.002	543	15.3	0.028	1911	13.2	0.007	46	17.6	0.383	526	15.5	0.029
6e-04	4902	8.2	0.002	510	13.1	0.026	1853	11.3	0.006	38	15.1	0.397	501	13.3	0.027
5e-04	4764	6.8	0.001	466	10.9	0.023	1766	9.4	0.005	33	12.6	0.382	453	11	0.024
4e-04	4598	5.4	0.001	427	8.8	0.021	1663	7.5	0.005	24	10.1	0.421	407	8.8	0.022
3e-04	4375	4.1	0.001	397	6.6	0.017	1545	5.7	0.004	17	7.6	0.447	364	6.6	0.018
2e-04	4112	2.7	0.001	339	4.4	0.013	1402	3.8	0.003	10	5	0.5	294	4.4	0.015
1e-04	3683	1.4	0	275	2.2	0.008	1206	1.9	0.002	4	2.5	0.625	237	2.2	0.009

For a p-value cutoff p, the expected number of false discoveries (FD) is $\pi 0Np$ (N is the total number of transcripts), the number of discoveries (D) was the number of transcripts with p-values < p, and FDR is calculated as FD/D.
